# Choices and options in the care procurement process of bundled payment contracts: A literature-based overview from a payer’s perspective

**DOI:** 10.1371/journal.pone.0346366

**Published:** 2026-06-15

**Authors:** Sander Steenhuis, Jeroen van der Wolk, Eric van der Hijden

**Affiliations:** 1 Department of Health Sciences, Health Economics Section, Talma Institute, VU University Amsterdam, Amsterdam, the Netherlands; 2 Mentaal Care Group, Hilversum, the Netherlands; 3 Department of Health Sciences, Health Economics Section, Talma Institute, VU University Amsterdam, Amsterdam, the Netherlands; 4 Zilveren Kruis, Care Procurement Department, Leusden, the Netherlands; Universidade de Lisboa Instituto Superior de Ciencias Sociais e Politicas, PORTUGAL

## Abstract

**Context:**

While the potential of bundled payment models to facilitate value-based care is hard to overestimate, the complexity of designing an effective and feasible care procurement contract forms a formidable barrier to their use. The objective of this study is to identify, structure, and interpret the key design choices and options during the care procurement process of bundled payment contracts using a payer’s perspective.

**Methods:**

Two reviewers independently conducted an iterative qualitative content analysis of a selectively sampled set of articles from a prior scoping review. First, they extracted and clustered all text excerpts about design choices or options relevant to a bundled payment procurement process. Where available, they assessed the potential impact of choosing that option. Second, the choices and options were analytically organized into six phases of a care procurement process. Third, the resulting framework was evaluated in an external expert panel session.

**Findings:**

33 design choices with 110 unique options were identified and structured within a framework. An example of a design choice is to ‘determine the method to select care and cost to include in the bundle,’ in which case 4 options were identified: 1. based on historic claims data, 2. based on best-practices and/or local care pathways, 3. based on evidence-based clinical guidelines, and 4. based on the provider type or specialism that is willing to collaborate.

**Conclusions:**

This study helps understand how design choices for payers to purchase value-based care with bundled payment contracts are context-dependent (e.g., in a single-payer system options are different than in a multi-payer system), interchangeable (i.e., different choices can achieve similar effects) and/or interrelated (i.e., choosing one option can have a positive or negative impact on the options in other procurement phases). The framework assists payers (and providers) in developing an effective and feasible bundled payment contract.

## Introduction

The majority of current provider payment models contain a perverse financial incentive for providers to increase the volume, rather than the value, of care. At the same time, they deter providers from investing in innovations, enhancing integration and changing their care delivery towards achieving higher quality outcomes for their patients by not reimbursing them [1,2]. In fact, providers could even feel financially penalized for these sorts of efforts since the time and human resources spent on innovation are not spent on treating more patients (i.e., by increasing volume) and could therefore reduce their income [3].

For decades many health systems around the world have aimed to replace these failing provider payment methods with a promising Alternative Payment Model (APM) called bundled payments [4,5]. Bundled payments are designed to reward providers for more efficient and better integrated care delivery while at the same time improving the outcomes for patients and lowering their costs. On the one hand bundled providers can retain (part of) the savings if they manage to keep their costs lower than the predetermined price in the bundled payment contract with the payer (i.e., if quality standards are maintained or improved). On the other hand these contracts can also introduce financial risk for providers by having them share in the losses if the costs of their care exceed the negotiated bundle price [1,2].

Bundled payment contracts are quite different from traditional volume-based contracts. They contain various new design choices and require a different type of collaboration between payer and provider(s) [6–8]. Designing and negotiating bundled payment contracts typically requires a series of interrelated technical and governance decisions, including defining the episode (scope, services, time window, etc.), setting attribution rules, and determining how prices account for patient risk profiles [9–11,12]. In addition, parties often need to agree on risk-mitigation arrangements (e.g., outlier policies, risk corridors or caps) and on how financial incentives are distributed across participating providers. These contract design challenges are repeatedly identified as key barriers to implementation and scaling of bundled payment initiatives [3,13,8]. Also, the detailed terms of the few existing bundled payment contracts are usually confidential and all the information that has been published in the last 15 + years is highly fragmented within a large mass of scientific and grey literature. This makes it difficult for payers and providers who are just starting out with bundled payment contracts to identify and translate all the available knowledge and experiences into an effective and feasible care procurement strategy. A clear overview of the choices and options involved in the design and implementation of such contracts would help but is currently lacking. As a consequence, bundled payments are often seen as too complex to implement and the adoption of these promising value-based contracts is slow.

Although a recently published scoping review [7] already provides valuable insights on this specific topic, its lessons are not easily applicable by payers who want to design and implement a bundled payment contract in practice. Accordingly, the aim of this follow-up study is to identify, structure, and describe the various choices and options for payers in the procurement process of bundled payment contracts, based on the literature and the experiences of innovators and early adopters.

## Methods

### Study sample

We performed a qualitative content analysis (QCA) on a subset of articles that were included in the final study sample of a scoping review, which was published in The Milbank Quarterly in January 2020 [7]. In this initial study the aim was to unravel and understand better the complexity in the design and implementation process of bundled payment contracts compared to traditional fee-for-service contracts. The authors were able to identify 53 key elements related to this process from the literature, and then to develop a framework in which they arranged those elements according to the different phases of a care procurement process and those involved in the process at various levels of the health care system [7]. Although informative, the downside of that framework is that the identified elements are described in general terms and are not straightforward to be put into practice either by payers nor providers.

In the current study, two reviewers (*[initials omitted for peer review process]*) leveraged the sample (containing 147 articles) of the initial study to identify independently all text excerpts referring to design choices or options relevant to a bundled payment procurement process (using a payer’s perspective) and extracted them to Microsoft Excel. When, during the iterative process in which they compared and discussed their extracted text excerpts, choices, and options, *[initials omitted for peer review process]* could not reach a consensus on whether to include them in the final selection, a third reviewer (*[initials omitted for peer review process]*) made the final decision. To establish our final study sample, selective sampling was used by starting the analysis with the article that contained the highest number of identified key elements from the initial study. We then selected the article with the second most identified key elements and continued this process until we reached the point at which no text excerpts describing new choices or options were identified. We assumed that articles rich in key elements were more likely to yield a higher number of relevant text excerpts on design options. Next, the described choices and options were extracted from those text excerpts and entered into separate columns in the Excel sheet. If an article also described the impact, lessons learned and/or experiences gained from choosing (or not choosing) a specific option, then that description was extracted as well and entered in an additional column. Appendix A in S1 Appendix contains the original text excerpts extracted from the included articles. Each excerpt is assigned a unique reference number. These reference numbers are retained throughout the analysis and are used consistently in Appendix B in S2 Appendix to indicate the textual basis for each reported impact statement. As such, every impact description in Appendix B in S2 Appendix can be directly traced back to one or more original excerpts in Appendix A in S1 Appendix via the corresponding reference number(s), enabling transparency and reproducibility of the coding and synthesis process.

### Qualitative content analysis

To extract as many bundled payment contract design choices and options as possible from the final study sample, the two reviewers (*[initials omitted for peer review process]*) analyzed the text excerpts by using the broadly used (inductive) qualitative content analysis process of Elo and Kyngäs [14]. This QCA process consisted of five phases: 1. Open coding, in which we coded all the relevant text excerpts containing descriptions of design choices or options (and, where available, their impact) in the sample. 2. Developing a coding sheet, in which all open codes were collected, and design choices and options (and, where available, a description of their potential impact) were extracted from the text excerpts into separate columns. 3. Grouping, in which we merged the design options that were similar or related into one option through constant comparison. 4. Categorization, in which we categorized the merged design options into broader higher order design choices through interpretation. Phases 1–4 were simultaneously executed in an iterative process with multiple consensus rounds, requiring the two reviewers (*[initials omitted for peer review process]*) to engage with each grouping or categorization decision reflexively, and repeat steps when necessary. Saturation was not assessed at the level of individual text excerpts (steps 1 and 2), but at the level of grouping and categorizing the design choices (steps 3 and 4). Articles were iteratively added until no new design choices emerged and the categorization of existing choices remained unchanged. Interpretation was explicitly applied during the grouping and categorization phases of the qualitative content analysis, particularly when merging similar design options and when positioning higher-order design choices within the procurement framework. Where impacts or relationships were not literally described in the source articles, logical deductions were made based on the purpose of the design choice and its role in the procurement process. Eventually, the QCA process ended with phase 5. Abstraction in which we clustered the design choices into logical main categories (i.e., procurement phases) to create a coherent framework.

### The bundled payment procurement framework

During the fifth phase of the QCA process, in order to cluster the design choices in a way that is not only generic and recognizable to payers but also operationally applicable as a useful structure to first explore their options and then develop their local bundled payment implementation strategy, we constructed a framework with six common phases for a generic care procurement process (as is currently also used in fee-for-service contracting). This framework is based on existing frameworks for health care procurement using a payer’s perspective [15–17], and extends the framework we constructed for the initial study [7] by including a more strategic overarching preparation phase with several fundamental design choices for the payer to consider in order to explore and determine the initial situation before the actual procurement process begins. After this preparation phase the process generally starts with a (more tactical) precontractual phase in which: 1. purchasing needs are specified (e.g., defining the care services to be included in the bundle), 2. providers are selected and then, 3. contracted. This is then followed by a (more operational) postcontractual phase in which the patients are, 4. identified and allocated to the bundle, 5. the care is delivered and monitored and eventually, 6. ends with the actual payment for care and the evaluation of the contract. More detailed information about the specific phases of the framework can be obtained from the previous (open access) publication in The Milbank Quarterly [7].

We decided to position each design choice in the procurement phase to where it has the most impact on the subsequent phases in the procurement process. For example, the design choice involving opting either for a contract with a ‘voluntary or mandatory nature’ is positioned in the second phase (i.e., the ‘select providers’ phase) of the framework because that decision has a significant impact on the available options in many of the design choices further into the procurement process (e.g., when choosing a ‘governance model’ or a ‘claims-processing procedure’), but not necessarily on the design choices earlier in the procurement process (e.g., when specifying which ‘care and costs to include in the bundle’). This impact-based positioning of design choices in the most appropriate phases of the framework was based on the literature where possible, and on the researchers’ own interpretation if the impact was not literally mentioned in the article but could logically be deduced from it (after a consensus round). Additionally, the positioning was validated during the expert panel session.

### Expert panel session

At the end of the qualitative content analysis process in which the two researchers (*[initials omitted for peer review process]*) had ordered the identified design choices from the final study sample in the framework, we organized an expert panel session. The goals of this two-hour session with 19 experienced care procurement professionals from four different payer organizations were to assess externally and independently the recognizability and completeness of (1) the identified design options and choices (Tables 3–9 in the results section), and (2) their positioning in the framework (Tables 1 and 2 in the results section). The expert panel consisted of nineteen care procurement experts from the four largest Dutch health insurance companies (which together account for over 90% of the Dutch health insurance market) and represented a range of roles in strategic purchasing, contract design, and the implementation of bundled payment initiatives. More detailed information about (the methodology applied during) the expert panel session can be obtained from Appendix C in S3 Appendix.

**Table 1:** Framework with 33 choices in the design and implementation of bundled payment contracts (part 1: preliminary and precontracting phase).

BUNDLED PAYMENTS AS PROCUREMENT STRATEGY	PROCUREMENT PROCESS		
Preliminary Phase	Precontracting Phase		
Fundamental Choices	1. Specify the Bundle	2. Select Provider(s)	3. Negotiate, adjust and sign contract
1. Transition strategy from FFS to full bundled payment	5. Type of care	13. Mandatory or voluntary	17. Level of contract flexibility
2. Alignment with other payers	6. Patient group/ population	14. Governance model	18. Duration of the contract
3. Payment strategy	7. Length of the bundle	15. Completeness of providers	19. Method for price calculation
4. Trust, collaboration and commitment	8. Method to select care and costs	16. Criteria to select providers	20. Price negotiation method
	9. Insurance risk mitigations for providers		21. Price level
	10. Method for accountability on quality		22. Method for case mix adjustment
	11. Set of quality indicators		23. Type of stop-loss provisions
	12. Type of performance targets		24. Type of risk sharing

**Table 2:** Framework with 33 choices in the design and implementation of bundled payment contracts (part 2: postcontracting phase).

PROCUREMENT PROCESS		
Postcontracting Phase		
4. Identify and Include patients	5. Deliver and Monitor Specified Bundle	6. Payment and (Financial) Results
25. Incentives for patients	27. Claims-processing procedure for payer	31. Support for payment distribution
26. Criteria to identify patients	28. Moment of price adjustment	32. Potential side effects
	29. Digital infrastructure for data	33. Effects on care and costs and how to proceed
	30. Support for redesign of care delivery	

**Table 3:** Choices and options in the preliminary phase of bundled payment procurement.

FUNDAMENTAL CHOICES			
NO.	DESIGN CHOICE	DESIGN OPTIONS	REFS
1	Determine the transition strategy from FFS to full bundled payment	A. Stick with just limited changes in the FFS-based DRG model	[3,11,18,19,20,21]
		B. Stepped-model approach based on included care	[11,18,22,25,26,27,21]
		C. Stepped-model approach based on the distribution of risk	[9,10,22,28,26,27,29,30,31]
		D. Immediate transition from FFS to full bundled payment (no stepped model)	[26]
2	Determine the strategy to align the new payment method with other payers	A. Establish alignment agreements with other payers	[3,25,27,12]
		B. Do not establish alignment agreements with other payers	[3,8,11,32]
3	Determine the payment strategy to incentivize providers	A. Use a retrospective payment strategy	[8,23]
		B. Use a prospective payment strategy	[8]
		C. Offer both retrospective and prospective payment strategies	[24]
4	Determine the strategies to acquire trust, collaboration and commitment among all involved stakeholders	A. Acquire strong commitment and clear support from senior management (on payer and provider side)	[10,24,25]
		B. Develop a clear communication strategy to stimulate providers to participate in the contract	[10,25,18,33]
		C. Involve care professionals in the design of the contract	[10,18,34,19,27,29]
		D. Align gain sharing methods for care professionals in the bundle	[3,23,35,21]
		E. Define clear goals and a definition of success that is aligned among all stakeholders	[10,18,24,25,26,31]
		F. Allow external investments from third parties	[8,24,25,28]

**Table 4:** Choices and options in the ‘specify the bundle’ phase of bundled payment procurement.

PHASE 1: SPECIFY THE BUNDLE			
NO.	DESIGN CHOICE	DESIGN OPTIONS	REFS
5	Determine the type of care for the bundle	A. Procedure based	[10,11,24,27,21]
		B. Condition based	[10,24,21,30]
		C. Population based	[23,30]
6	Determine the patient group or population for the bundle	A. Choose a patient group that is relatively homogeneous, high volume and with low comorbidity rates	[9,18,35,19,33,29]
		B. Choose a patient group that is relatively heterogeneous, with large cost variations and in which multiple providers are involved	[20,29]
7	Determine the length of the bundle	A. Based on a clinically logic pre- and/or post-episode period	[22,29,12]
		B. Based on a fixed time period (e.g., a year)	[23,19]
8	Determine the method to select care and costs for in- or exclusion from the bundle	A. Based on historic claims	[12]
		B. Based on best practices and/or care pathways	[9,11,27,30]
		C. Based on evidence-based clinical guidelines	[10,11]
		D. Based on the provider type or specialism that is willing to collaborate	[24]
9	Determine insurance risk mitigations for providers in the bundle	A. Exclude rare high-costs, high-risk patients and/or complex complications from the bundle	[3,22,33]
		B. Allow providers to select a portfolio of (higher volume) bundles	[22,24]
		C. Do not mitigate insurance risk for providers	[3,10,34,32]
10	Determine the method to hold providers accountable for the quality of care in the bundle	A. Payment is not tied to quality indicators	[9]
		B. Payment is tied to quality indicators (that providers can control)	[3,9,27]
		C. Payment is tied to quality indicators on a multi-provider level	[3]
		D. Use a neutral third-party, provider advisory council, or community organization to define, collect and analyze quality data	[3,24]
11	Determine the set of quality indicators for the bundle	A. Measure and monitor a broad set of quality indicators	[3,8,24,19,31,12]
		B. Measure and monitor a narrow set of quality indicators	[8,31]
12	Determine the type of targets to measure performance of providers in the bundle	A. Absolute performance targets	[31]
		B. Relative performance targets	[8,31]
		C. Both absolute and relative performance targets	[31]

**Table 5:** Choices and options in the ‘select provider(s)’ phase of bundled payment procurement.

PHASE 2: SELECT PROVIDER(S)			
NO.	DESIGN CHOICE	DESIGN OPTIONS	REFS
13	Determine the mandatory or voluntary nature of participating in the contract	A. Mandatory participation	[11]
		B. Voluntary participation	[3,11,27]
14	Determine the completeness of the selected providers in relation to the full cycle of care/patient journey	A. Bundle services of only one provider	[35,33]
		B. Bundle services of more than one providers (but not the full patient journey)	[11,23,35,27,33]
		C. Bundle the full cycle of care (full patient journey)	[9,23,35]
15	Determine the governance model to contract participating providers	A. Contract with one provider who can provide all care included in the bundle (without subcontracting)	[9,21]
		B. Contract with one provider as main contracting party who subcontracts with other providers	[9,23,24,29,21]
		C. Contract with multiple providers separately	[24,21]
		D. Contract with multiple providers separately using a virtual bundle	[18,32]
		E. Contract with a third party intermediate/ convening organization	[22]
16	Determine the criteria to select eligible providers for the contract	A. Select providers based on formal characteristics (e.g., patient volume, interoperability and/or quality profile)	[22,24,28,19]
		B. Select providers based on informal characteristics (e.g., motivation, commitment and/or experience)	[24,34]
		C. Select providers based on cost-saving opportunities	[24]
		D. Select providers based on the degree of integration in their (local) system	[30]
		E. Select (patients of) providers with retrospective statistical attribution rules	[3,11,12]

**Table 6:** Choices and options in the ‘negotiate, adjust and sign contract’ phase of bundled payment procurement.

PHASE 3: NEGOTIATE, ADJUST AND SIGN CONTRACT			
NO.	DESIGN CHOICE	DESIGN OPTIONS	REFS
17	Determine the level of flexibility of the contract terms	A. Develop model contracts in which terms can be negotiated postcontractual if unexpected events occur	[3]
		B. Develop model contracts with (pre-contractual) context-dependent terms based on market conditions, organizational form and/or ownership	[8,26,20]
		C. Develop model contracts with (pre-contractual) context-dependent terms based on the providers’ patient population	[26]
		D. Develop model contracts in which all terms are (pre-contractual) negotiable except for the bundle definition	[24,33]
18	Determine the duration of the contract	A. Yearly contract	[19]
		B. Multi-year contract	[3,19]
19	Determine the method for calculating the price of the contract	A. Based on historic service use and cost patterns (no benchmark with other providers)	[11]
		B. Based on internal cost benchmarks between providers (i.e., data of one payer)	[11]
		C. Based on external cost benchmarks between providers (i.e., data of all payers)	[11]
		D. Based on a blend of the provider’s historic spending and regional/national average spending	[22]
		E. Based on the care pathway and/or guideline-based standards	[11,30]
		F. Based on Time driven activity‐based costing (TDABC)	[8,19]
20	Determine the price negotiation method	A. Negotiate a price based on the individual bundle items	[9]
		B. Only negotiate on the total bundle price	[9]
21	Determine the price level for the bundled payment contract	A. Set a relatively low price (compared to the sum of historic FFS prices)	[3,25,20,27,33]
		B. Set a relatively high price (compared to the sum of historic FFS prices)	[3,8]
		C. Develop multiple price categories within the same bundle (e.g., based on the severity of the patient’s condition)	[3,23]
22	Determine the method for case mix adjustment	A. No risk adjustment	[3,9,26,32,33]
		B. Use relatively simple risk adjustment systems	[22,27]
		C. Use relatively complex risk adjustment systems	[3,32,33]
23	Determine the type of stop-loss provisions	A. Stop-loss provisions at the patient level	[9]
		B. Stop-loss provisions at the level of a single provider	[3,12]
		C. Stop-loss provisions at the level of multiple providers	[3,22]
		D. No stop-loss provisions	[3,33]
24	Determine the type of risk sharing	A. Only upside risk	[31,12]
		B. Only downside risk	[3,8,31]
		C. Upside and downside risk	[8,10,31,12]

**Table 7:** Choices and options in the ‘identify and include patients’ phase of bundled payment procurement.

PHASE 4: IDENTIFY AND INCLUDE PATIENTS			
NO.	DESIGN CHOICE	DESIGN OPTIONS	REFS
25	Determine the incentives for patients to use bundled care	A. Create benefit design changes for patients who use bundled providers	[9,11,25,27,33,21]
		B. Provide transparency in total bundle costs for patients	[3,9,32,19]
		C. Engage patients with patient satisfaction information	[24]
		D. Designate the bundled providers as centers of excellence	[11,33]
		E. Educate and create awareness by referring providers (e.g. GPs) on the advantages of bundled care for their patients	[21]
		F. Accept that not all patients can be incentivized to receive care from bundled providers	[25]
26	Determine the criteria for patient inclusion and the process for providers to be able to identify them in the data	A. Attribute patients prospectively (on the provider side) by using clinical identification criteria	[11,22,21]
		B. Attribute patients retrospectively (on the payer side) by using historic claims data	[11]

**Table 8:** Choices and options in the ‘deliver and monitor specified bundle’ phase of bundled payment procurement.

PHASE 5: DELIVER AND MONITOR SPECIFIED BUNDLE			
NO.	DESIGN CHOICE	DESIGN OPTIONS	REFS
27	Determine the payer claims-processing procedure for care related to the bundle	A. Use a manual claims reconciliation process based on the existing FFS infrastructure	[24,33]
		B. Use an automatic claims reconciliation process based on the existing FFS infrastructure	[10,25]
		C. Adopt a completely new bundled payments claims-processing procedure	[24]
		D. Use unique identification codes for patients which moves with them (and links the provided care) across different providers	[29,12]
28	Determine the moment of bundle price adjustment	A. Update bundle prices quarterly	[22]
		B. Update bundle prices annually	[22]
		C. No update of bundle prices	[12]
29	Determine the digital infrastructure to share and analyze data	A. Create one central multi-stakeholder database	[3,23,25,18,35]
		B. Integrate the existing digital infrastructure of providers	[9,22,24,25,28]
30	Determine the type of involvement in the redesign of care delivery	A. Support the exchange of patient data between providers to improve care coordination	[10]
		B. Support providers in developing standardized care protocols and processes	[22,35]
		C. Support providers by facilitating the involvement of neutral third-party coordinating organizations	[22,24,25]
		D. No support for providers to develop integrated care networks	[3,24]

**Table 9:** Choices and options in the ‘payment and (financial) results’ phase of bundled payment procurement.

PHASE 6: PAYMENT AND (FINANCIAL) RESULTS			
NO.	DESIGN CHOICE	DESIGN OPTIONS	REFS
31	Determine the type of involvement in the distribution of the payment and/or savings/losses among bundled providers	A. Support providers with the distribution of payment	[10,27]
		B. Involve a third party claims adjudicator to distribute payment	[24,33]
		C. No support: providers make their own arrangements to distribute payment	[9,26]
32	Determine the potential side effects of the contract	A. Evaluate the impact on provider market conditions	[9,25,21]
		B. Evaluate the impact on payer market conditions	[25]
		C. Evaluate undesired “gaming” effects	[26,21]
		D. Evaluate potential “crowd-out” effects of financial incentives	[8]
33	Determine the effect of the contract on care and costs and how to proceed with the contract	A. Do not extend the contract	[24]
		B. Extend the contract (with adjustments if needed)	[3]

## Results

### Study retrieval

During the selective sampling process of the initial study sample we reached our saturation point at 24 articles, which became the final study sample [3,8,9–12,18–35]. By this time we had identified 409 relevant text excerpts from this sample describing one or more design choices or options (and, where available, their impact) relevant to the procurement process of a bundled payment contract. At this point, additional articles did not yield new design choices nor materially alter the structure of the emerging framework, which confirmed analytical saturation at a final sample of 24 articles. After completing the QCA process and expert panel session, this eventually resulted in a list of 110 unique options, categorized into 33 design choices.

### Design choices and options in bundled payment procurement

Tables 1 and 2 show the 33 design choices related to the design and implementation of a bundled payment contract, categorized in the six phases of a health care procurement process. The framework presents a structured overview of all design choices we identified from the literature, and shows during which phase of the procurement process they have the biggest impact (i.e., according to the literature, the authors’ interpretation of the text excerpts and/or the feedback from the expert panel session). As Tables 1 and 2 show, each procurement phase is impacted by multiple design choices. The positioning of design choices within procurement phases was based on the literature where explicitly described, and on the researchers’ interpretation where the impact on subsequent phases could be logically deduced but was not explicitly stated.

Tables 3–9 show an overview of the 33 design choices with, in total, the 110 unique design options we identified. For more detail see Appendices A and B, containing the 409 original text excerpts we extracted from the final study sample (Appendix A in S1 Appendix) and the descriptions of the (positive and/or negative) impact of each design option (Appendix B in S2 Appendix). These short impact-descriptions in Appendix B in S2 Appendix are all based on the text excerpts we identified in our final study sample and are always linked to the original text excerpts in Appendix A in S1 Appendix with a reference number (e.g., ‘2.02.1’ or ‘15.02.3’). In most cases these descriptions are direct quotations from the articles in our final study sample. In some cases we summarized and/or edited an original text excerpt to maintain consistency in terminology and improve the readability of this paper. Some overlap between design choices is inherent to the nature of bundled payment procurement. Several choices address similar underlying challenges, such as risk mitigation, but from different procedural or analytical perspectives within the procurement process. Rather than enforcing artificial separation, we deliberately retained this overlap to reflect real-world contracting practice.

Next we describe the framework and its design choices and options (Tables 3–9), following the care procurement phases as introduced in Tables 1 and 2. Due to the restricted length of this article, we limit our descriptions of design choices to one choice for each procurement phase that is frequently identified and accepts as typical for bundled payment procurement, as opposed to traditional (fee-for-service) procurement. For those choices we also describe relevant examples of the potential (positive or negative) impact of some of the design options. For full details on all literature-based potential impact-descriptions we refer to Appendix B in S2 Appendix.

Concerning the interpretability of the results section, when we refer to a “payer” we mean any organization that enters into contracts with and subsequently pays care providers for the care they deliver. Depending on the particular country or health system, this could be a private insurance company, a public funding body or a (local or national) government. When we refer to a “provider,” we mean a “provider organization” (which can contract with a payer, such as a hospital or primary care clinic), rather than an individual care professional.

In some of the design choices in Table 3–9 the options are Mutually Exclusive and Collectively Exhaustive (MECE format), in which case choosing one option automatically excludes the others and together the options contain all relevant possibilities within that choice (e.g., design choice 13 concerning the mandatory or voluntary participation of providers). Some other design choices contain non-MECE options in which the options do not necessarily exclude each other and together may not contain all relevant possibilities within the choice (e.g., design choice 4 concerning trust, collaboration and commitment). Most choices, however, are in a ME-format in which choosing one option automatically excludes the others but taken together the identified options may not contain all relevant possibilities within the choice (e.g., design choice 28 concerning the moment of bundle price adjustment which obviously has many options, but only two of them were described in our study sample) [36].

In a few cases there can be overlaps between the options of different design choices. In such cases, however, the options relate to a similar topic or issue in bundled payment procurement, but from a different perspective. An example is the overlap between the options of design choice 5 about the ‘type of care’ with the options of design choice 6 about the ‘patient group or population.’ Although these options may overlap (e.g., option 5A with 6A and option 5C with 6B), we have identified both perspectives in the literature and have therefore decided to position them as separate choices.

### Preliminary phase: Fundamental choices

We identified 4 design choices which are so fundamental and/or overarching for introducing bundled payments as a procurement strategy, that they usually take place before the actual procurement process starts (Table 3).

One of the fundamental choices a payer has to make in the preliminary phase is ‘how to transition from its current traditional (fee-for-service) model to full bundled payments’ (design choice 1 in Table 3). We identified 4 design options related to this choice. The first option (A) is to ‘stick with limited changes in the FFS-based DRG model’ by simply expanding the scope of a DRG payment (e.g., one integrated payment for 3 or 4 DRG’s related to the same condition within one provider). Although this ‘more limited version of an episode-based payment could eventually become a building block for a full-fledged bundled payment system’ (4.04a.1) [11], most related text excerpts we identified in the literature about the potential impact of this option describe a ‘negative’ impact (see choice 1 in appendix B in S2 Appendix). For example, this option ‘falls far short of value-based bundled payment principles’ (17.02.2) and the ‘effects on outcomes may be smaller’ (11.06.2) [18,19].

Options B and C in the design choice about ‘determining the transition strategy’ (design choice 1) are both about a ‘stepped-model approach’ in which the impact and complexity of the contract is phased in over time. In option B this is done by gradually expanding the care services included in the bundle, and in option C by gradually introducing more risk for providers (e.g., by starting out with an ‘upside only’ payment strategy). For both options we mainly found ‘positive’ descriptions of their potential impact. For instance, a stepped model approach could ‘reduce the threshold for providers to participate’ (5.01.1) [22]. The fourth option (D) is an ‘immediate transition from fee-for-service to full bundled payment’ (i.e., in which most care related to the full patient journey is included in the bundle and providers take on most of the performance risk). On the positive side this option may ‘introduce less challenges for evaluators’ (such as the Agency for Healthcare Research and Quality) (14.06.2a), but on the negative side it may also introduce more challenges ‘given providers’ reported difficulty adjusting administrative staffing, finances, and provider relationships to the new reimbursement regime’ (14.06.2b) [26].

### Precontractual, phase 1: Specify the bundle

In this phase, in which purchasing needs are specified and the care bundle is designed, we identified 8 design choices (Table 4).

One of the most frequently identified design choices in our study sample is about ‘determining the method to select care and costs for in- or exclusion from the bundle’ (design choice 8 in Table 4), for which we found 4 different design options. In option A care and costs are selected ‘based on historic claims’ of care utilization, which is currently the most frequently used approach in bundled payment contracts [37]. Then, while selecting care and cost ‘based on best practices and/or care pathways’ (option B) or ‘based on evidence-based clinical guidelines’ (option C) may seem rather similar at first, there are in fact several differences. The main one is that ‘services recommended in evidence-based clinical guidelines often constitute only a portion of the cost of an episode of care, which would create an underestimation of the actual cost of the episode’ (2.01.2) [10]. In contrast, when a bundle is based on standardized and clinically logic care pathways then ‘providers may be better able to understand and reasonably predict the full range of services that a patient might need during the course of a typical episode’ (4.03a.1). Also, ‘payers may then be more confident about linking financial incentives to those episodes with the expectation that providers will respond appropriately by enhancing care coordination’ (4.02.1) [11]. A fourth option is to select care and costs for the bundle ‘based on the provider type or specialism that is willing to collaborate’ (option D). ‘Not including the services of a certain provider type or specialism if they are not (willing to be) at the table, improves the process of team building and contracting discussions and creates a more open and flexible approach that is essential to successfully negotiating a bundled payment contract’ (7.05.1) [24].

### Precontractual, phase 2: Select provider(s)

In this phase, in which the eligibility of providers is determined, we identified 4 design choices (Table 5).

An example of a design choice during this phase is to ‘determine the governance model to contract participating providers’ (design choice 15 in Table 5) (i.e., which is only relevant in case the included care, as determined in phase 1, involves more than one provider). Within this choice, the first option (A) is a ‘contract with one provider who can provide all care included in the bundle’ (without the need to subcontract other providers). ‘In this situation, the provider controls every aspect of the care episode within the bundle and thus can most effectively manage costs and maximize profit’ (1.01.1a). ‘However, in reality most local and regional health care situations do not have a single source provider but are composed of multiple providers for each and every part of the episode of care’ (1.01.1b) [9]. The second option (B) is a ‘contract with one provider as main contracting party who subcontracts with other providers.’ From a payer’s perspective, such a governance model may reduce ‘challenges concerning the administrative and contractual arrangements required to implement and distribute a single payment across multiple independent provider organizations’ (21.04.1) [29]. However, ‘providers generally have limited infrastructure for paying for services delivered by downstream providers’ (6.13.1) [23]. Also, the ‘main providers may dominate the collaboration and act in their own interests, which may not be congruent with the others’ (22.07.2) [21]. The third option (C) is to ‘contract with multiple providers separately.’ On the one hand ‘the collaborating providers would only have to agree on a contract about how to distribute surpluses or pay back deficits, while a flow of core funding for each entity would be assured,’ but on the other hand this ‘approach of maintaining separate payment streams may to some extent undermine the bundled payment contract’s goal of true collaboration between providers’ (22.09.3a;b) [21]. A variation on this third governance model could be to ‘contract with multiple providers separately using a virtual bundle’ (option D). In this case there would be no contract between the separate providers, but each provider’s individual payment and/or savings would be based on their contribution to the joint performance of all the ‘virtually bundled’ providers. While the argument that such a model undermines the goal of a bundled payment contract still applies here, it may (for instance when there is yet no collaboration between providers) be better than ‘waiting until an organizational structure is in place that can accept a bundled payment and divide it in a way that the individual providers find acceptable’ [32]. The fifth option (E) within this choice is to ‘contract with a third party convener,’ which is usually an independently functioning intermediate organization (i.e., that does not deliver care itself). Such an organization can ‘provide the analytic and risk management services providers often lack the time and infrastructure for, and contract directly with the payer on behalf of the bundled providers’ (5.06.1) [22]. In this case there is no longer a direct financial interaction between the payer and the provider for care included in the bundle [33].

### Precontractual, phase 3: Negotiate, adjust and sign the contract

After the bundled care is specified and the eligibility of providers is determined, the pre-contractual phase typically ends with negotiations in which the payer and provider(s) aim for consensus on the precise terms of the contract. For this phase, we identified 8 design choices (Table 6).

An example of a design choice during this phase of the process is to ‘determine the method for case mix adjustment’ (design choice 22 in Table 6). Within this choice, the first option (A) we identified is: ‘no risk adjustment.’ An advantage of choosing this option could be that ‘a patient’s baseline risk score tends to increase as soon as they become part of a risk-adjusted payment system (because the provider now has an incentive to do complete coding of diagnoses), and this can cause overall spending to increase rather than decrease’ (3.04.1) [3]. However, the majority of text excerpts in our final study sample describe the disadvantages of this option. For example, ‘without risk-adjusting the bundled payment, providers may have concerns about the unprotected financial risk that they would incur if they operated on higher risk patients’ (19.03.2) [33] which gives them ‘a strong and undesirable incentive to avoid patients who have multiple or expensive-to-treat conditions’ (16.01.2) [32]. Additionally, ‘in the absence of robust risk adjustment, providers may game the system by changing coding practices to maximize reimbursement for the bundle (upcoding) or by moving services in time or location to qualify for separate reimbursement (unbundling)’ (14.03.1) [26]. Then, in order to make a distinction between risk adjustment systems, the literature-based impact descriptions were split into ‘relatively simple’ (option B) or ‘relatively complex’ (option C). Although these terms are rather arbitrary, in this study relatively simple risk adjustment systems can be considered as less accurate in their adjustments (i.e., limited compensations for variations in costs) but easier to implement (e.g., based on less case mix variables but also less expensive). Relatively complex risk adjustment systems can be considered as more accurate in their adjustments (i.e., strive for the most optimal compensation for variations in costs) but harder to implement (e.g., based on more case mix variables but also more expensive). For example, a relatively simple risk adjustment system could control for variables such as age and sex based on payer-data, while a relatively complex risk adjustment system could also control for more specific and clinically relevant patient data like kind of employment (e.g., heavy labor) and Body Mass Index, based on clinical data only available to the provider. While even a relatively simple risk adjustment system, ‘including some adjustment for patient severity reduces the risk of financial losses for the providers when they are taking care of sicker patients’ (18.04.1) [27], ‘risk adjustment based on complete clinical data on the patient’s past and current patient health conditions (not just on data recorded to support recent claims for payment to a particular health plan), can decrease the effect of an artificial increase in risk scores’ (3.04.2) [3]. Also, ‘more accurate risk adjustment could be used to limit risk without decreasing volume (as excluding higher-risk patients from the bundled payment would)’ (19.05.5) [33].

### Post-contractual, phase 4: Identify and include patients

After the contract is signed, patients eligible to be included in the contract must be identified and allocated during phase 4, for which we identified 2 design choices (Table 7).

An example of a design choice during this phase of the process is to ‘determine the criteria for patient inclusion and the process for providers to be able to identify them in the data’ (design choice 26 in Table 7). Within this choice, the first option (A) is to ‘attribute patients prospectively (on the provider side) by using clinical identification criteria.’ In this case, care professionals decide for each new patient whether to include him or her in the bundle based on specific clinical trigger events, risk stratifications and/or inclusion criteria that were determined during the specification phase of the contract. One study reported that in this process ‘inconsistencies in provider and payer data can lead to differences of 20 percent or more in the number of patients the provider identifies internally and the number identified in the payer data’ (5.10.1) [22]. The second option (B) is to ‘attribute patients retrospectively (on the payer side) by using historic claims data.’ Compared to option A, ‘limiting attribution to claims data would be easier for payers to implement and lower the risk that providers would engage in favorable selection of less-costly patients’ (4.03a.7a;b). However, it would also result in ‘less flexibility for payers to tailor attribution to specific clinical scenarios’ (4.03a.7.1a). In addition, ‘relying on claims data for attribution may also not reflect actual care relationships accurately, which might result in the contract having less face validity with providers’ (4.03a.7.2) [11].

### Post-contractual, phase 5: Deliver and monitor the specified bundle

After a patient is identified and allocated to the bundled care, (the monitoring of) the actual care delivery process starts, for which we identified 4 design choices (Table 8).

An example of a design choice during this phase of the process is to ‘determine the digital infrastructure to share and analyze data’ (design choice 29 in Table 8). As shown, we identified 2 design options within this choice. The first option (A) is to ‘create a new central multi-stakeholder database’ where all relevant data on claims, performance and quality metrics is accessible to all providers (and the payer) involved in the bundled contract. Such a database could facilitate implementation because it ‘enables stakeholders to perform claims-based analyses and track performance metrics across organizations and over time’ (9.01.1) [25]. However, as other text excerpts point out, ‘even if providers have access to claims data, most would not have the analytic capacity to assemble and analyze large claims databases, particularly if the data come from multiple payers. Also, there could be privacy concerns about giving providers patient-identifiable data about all services from other providers in order to find and combine multiple claims records for their own patients’ (3.08.2;3) [3]. Option (B) is to ‘integrate the existing digital infrastructure of bundled providers’ (rather than developing a new central database). While the practical organization of option B is profoundly different from option A, we identified comparable descriptions about the potential impact of choosing this option: on the one hand ‘transparent data sharing is important to obtain provider buy-in, build trust, and motivate transformation’ (7.06.1a) [24]. On the other hand ‘sharing clinical and claims data among providers can raise considerations of privacy, confidentiality, control over the data and antitrust law’ (9.05.1) [25].

### Post-contractual, phase 6: Payment and (financial) results

During the final phase of the care procurement process the contract effects are evaluated and payments are made. Related to this phase, we identified 3 design choices (Table 9).

An example of a design choice during this phase of the process is to ‘determine the potential side effects of the contract’ (design choice 32 in Table 9). As shown, we identified 4 design options within this choice and for each of the options we only identified ‘positive’ potential impact descriptions. The first option (A) is to ‘evaluate the impact on provider market conditions.’ Since ‘larger providers will likely be able to bundle care more effectively, monitoring market shares of providers could help to reduce the risk for monopolies’ (1.10.1) and prevent an ‘increased bargaining power that could raise prices higher than they would be if those providers were negotiating separately’ (22.13.1) [9,21]. The second option (B) is to ‘evaluate the impact on payer market conditions,’ which is also important because ‘a lack of significant competition for payers can dampen private insurers’ incentive for payment innovation’ (9.02.1) [25]. The third option (C) is to ‘evaluate undesired “gaming” effects,’ since there have been cases in which care professionals tried deliberately to “game” the payment model by influencing the process of care delivery to optimize their own personal (financial) benefits. Examples of this are: ‘the underuse of effective services within the bundle, avoidance of high-risk patients, and an inexplicable increase in the number of bundles reimbursed’ (14.01.1) [26]. The fourth option (D) is to ‘evaluate potential “crowd-out” effects’ because extrinsic motivators such as ‘financial incentives tend to “crowd-out” intrinsic motivation and may reduce providers’ commitment to professional norms and their inherent motivation to act ﬁrst and foremost in patients’ interest. Therefore, nonﬁnancial incentives must be considered in parallel, for example: reputation or brand, intrinsic motivation, and altruism’ (10.06.1) [8].

## Discussion

### Main findings

Over the last two decades, the innovators and early adopters in bundled payment procurement made many choices to design and implement their pilots, initiatives and contracts. The goal of this study was to reduce the complexity in bundled payment procurement by identifying those choices, make them more explicit, structure them, and then share them with payers (and other stakeholders) who are considering to use bundled payment as a procurement strategy. Through qualitative in-depth content analyses, 33 design choices (with, in total, 110 unique options) were considered relevant and structured in the six phases of a procurement process. We believe this comprehensive overview can enable payers (and providers) to change their thinking about bundled payment contracts as too complex to implement, to seeing them as, although still complicated, a feasible procurement strategy that holds significant potential to facilitate the delivery of value-based care for patients, if the right design choices are made.

### Limitations

This paper describes the results of a qualitative follow-up analysis based on the search strategy and sample of a previous study [7] and included an iterative process with two independent reviewers and multiple consensus rounds in order to select, group and categorize design choices and options. Despite the rigorous process, another search strategy and/or different reviewers may have resulted in some other design choices and options. Therefore, we do not claim this overview of choices and options to be complete, nor do we claim that the manner in which we positioned them according to the procurement phases is the only way to structure them. It is a unique explorative framework on which other researchers can build. Because this study aimed to synthesize and structure fragmented descriptions of bundled payment design choices rather than to perform a purely descriptive or linguistic analysis, interpretive synthesis was an inherent part of the methodology. While this strengthens the framework’s practical relevance, it also implies that some choices and options may be described, grouped, or labeled differently, particularly for choices and options that can reasonably be articulated in multiple ways or that depend on the researchers’ professional perspectives and prior experience.

It should be noted that the search strategy of the scoping review only includes generic key words (e.g., ‘bundled payment’ and ‘implementation’), rather than specific key words related to the particular design choices and options that were identified (e.g., ‘stop-loss provision’ or ‘risk mitigation’). Although an even more in-depth and richer analysis of design options could have been carried out, this would have required an additional literature search for each design choice separately, which was considered beyond the scope of this study.

Concerning the literature-based descriptions of the potential impact per design option in Appendix B in S2 Appendix, it should be noted that we aimed to preserve the original text excerpts from the final study sample as much as possible. However, in some descriptions of the potential impact we had to make minor adjustments in order to turn them into short and clear stand-alone pieces of text with a consistent writing style and terminology. In most cases it was clear whether an impact description was (potentially) positive (+) or negative (-) (see column 2 and 3 in Appendix B in S2 Appendix). In the cases in which this was unclear two researchers (*[initials omitted for peer review process]*) labeled the impact description as positive or negative based on their own interpretation, which was then checked by a third researcher (*[initials omitted for peer review process]*). Although these descriptions do provide some insight into the potential (positive or negative) impact of choosing a certain design option, they are far from firm conclusions. In the future a full systematic review, with an accurate quality assessment of included studies, may be appropriate in order to draw conclusions as to what the optimal (combinations of) design options are for an effective bundled payment contract (within a specific context).

The expert panel consisted of care procurement professionals who operate within the Dutch multi-payer health system. Although this reflects the context in which bundled payment contracting often emerges in a bottom-up manner, it may introduce contextual bias and limit the direct transferability of the findings to single-payer or more centrally standardized contracting environments.

The framework is based on published scientific and grey literature. Detailed contractual arrangements are almost never publicly available and may therefore not be captured. While the framework was designed to be generic and recognizable across health systems, its practical applicability and relevance may vary depending on factors like institutional arrangements, payer competition, and governance structures.

The study intentionally adopts a multi-payer perspective, in which payers typically develop contracts in a bottom-up manner and deliberately differentiate contract elements. This may limit transferring some of the findings to contexts with fundamentally different institutional arrangements, such as single-payer systems or more centrally standardized contracting environments

### Results in perspective

The objective of this study was to develop a generic procurement framework which would be recognizable to most payers in both the US and internationally in order to facilitate the design and implementation of bundled payment contracts on a larger scale. We therefore consulted literature from different health systems and analyzed a wide variety of bundled payment initiatives. The study and restructuring of the totality of this literature provided us with 3 main insights on how such a generic framework can be used for the development of a bundled payment procurement strategy.

**The first main insight is that the results of this study confirmed the obvious: namely that the design choices for payers to purchase value-based care with bundled payment contracts is to a large extent context-dependent.** A good example of this are the fundamentally different contexts of a single-payer versus a multi-payer health care system. In a single-payer system (or a multi-payer system with insufficient competition between payers) there may be a lack of incentives to innovate and experiment with new payment models [25]. In a system with multiple (competing) payers this lack of incentives may be less, but this also raises other challenges. For instance, for large scale adoption of bundled payment contracts in a multi-payer system, it may be necessary to make ‘(non-competitive) alignment agreements’ (design choice 2 in Table 1) between the competing payers in order to:

1. Prevent a significant increase in the administrative burden for providers if different payers purchase similar bundles of care in the same regions using different definitions, claim codes, quality indicators, etc.,
2. Tackle complicated issues regarding clients who ‘leak’ out off the bundle because, in a multi-payer system, they can usually switch between payers (e.g., in the Netherlands each year more than 7% of the population switches to another insurer [38]) or they can choose to go to a provider who is not engaged under the bundled payment contract. In a single-payer system the target group of a contract is much more stable, which makes it much easier to identify patients by merging all systems with patient data based on a single identification number per patient (e.g., in Ontario, Canada) [27,29].
3. Decrease the organizational and administrative costs for payers. Payers will benefit most from bundled payment contracts in regions where they have the highest market share. In order to contain administrative costs they would ideally also use these same contracts in regions where they have a lower market share, which would be difficult if the local market leader already contracts providers with a similar bundle of care that contains different definitions and standards,
4. Increase efficiency between payers by stimulating a certain degree of non-competitive uniformity within the contracts. To maintain adequate market conditions when working towards a more standardized type of contract (with for example national standards that clearly define which specific claim codes should be included in the bundle), the contractual agreements between payers and providers on bundle prices and shared savings should remain fully competitive. In this way the assumed advantages of regulated competition between payers can largely be preserved, while the implementation of bundled payments can benefit from shared investments in (for example) data infrastructure and reaching consensus on quality outcomes and patient experience measures. Some payers may even be inclined to collaborate more in order to increase local market share and make the participation in bundled payment contracts more attractive to providers (which is important because if providers only have a limited number of patients who are reimbursed under the bundled payment contract, it will be difficult for them to make the intended changes in care delivery). Nevertheless, reaching consensus in such collaborations can be complicated due to a history of conflicting interests between multiple competitive payers and is often in conflict with local anti-trust legislation [3,25,33],
5. Prevent potential free-rider behavior in which some payers take unfair advantage of the investments of others [3].

The abovementioned example illustrates how a different context (i.e., a multi-payer instead of a single-payer system) can result in very different operational challenges and design choices relevant to bundled payment procurement. An ‘off-the-shelf’ bundled payment implementation approach does not exist. Even when predefined bundle definitions (such as the ‘episode catalogue’ that is currently being developed by the PACES Center [39] or the ‘bundle definitions’ that were recently published by the Dutch Healthcare Authority (NZa) [40]) are used, there will always be a need to tailor bundled payment contracts to local circumstances and preferences [41].

**The second main insight is that our results illustrate how design choices in bundled payment contracting are sometimes interchangeable:** while they are different, some aim to achieve a similar effect. For example, throughout the precontractual phase of the framework there are multiple choices that relate to the topic of ‘provider risk mitigation,’ because there are different ways to achieve it. First, risk mitigation strategies can be applied in phase 1 when specifying the bundle. For instance, by choosing for a ‘patient group that is relatively homogeneous, high volume and with low comorbidity rates’ (design choice 6, option A), or by choosing prospectively to ‘exclude rare high costs, high-risk patients and/or complex complications from the bundle’ (design choice 9, option A). Both choices mitigate provider financial risk because an uncomplicated patient group generally reduces the risk of unexpected high costs [9]. Secondly, risk mitigation strategies can be applied in phase 2 when selecting providers for the bundled payment contract. For instance, by ‘bundling services of only one provider’ (design choice 14, option A) (because that provider won’t be held accountable for the costs of other providers), or by ‘determining provider eligibility criteria like minimum patient volumes and/or the degree of experience with bearing performance risk’ (design choice 16, options A and B). Thirdly, risk mitigation strategies can be applied in phase 3 when negotiating and adjusting the contract. For instance, by ‘setting a relatively high price’ for the bundle (design choice 21, option B), or by ‘using risk adjustment systems’ (design choice 22, option B or C), or by ‘including stop-loss provisions’ (design choice 23, option A, B and/or C), or by choosing for a one-sided risk arrangement and having providers ‘only share in upside risk’ (design choice 24, option A). The choices that are made in these various examples related to provider risk mitigations can either strengthen or weaken each other, and in some cases they are interchangeable. For example, specifying a relatively high-volume and uncomplicated patient group in phase 1 can make additional risk adjustment in phase 3 redundant [22].

**The third main insight is that our results also illustrate how the design options for payers to purchase value-based care with bundled payment contracts are interrelated:** choosing one option can reduce the options in other design choices and in other phases of the procurement process. For example, choosing to bundle care services of only one provider instead of those involved in the full patient journey (design choice 14, option A) will ‘eliminate’ options B (a contract with a main provider who subcontracts other providers), option C and option D (both contracts with multiple providers separately) in design choice 15 about determining the governance model (because, when choosing option A in design choice 14, there is only one provider involved in the bundled care). Another (less obvious) example is that choosing for a prospective payment strategy in which the bundle price is paid before the care has been delivered (design choice 3, option B) in the preliminary phase of the process, could ‘eliminate’ options A (using a manual claims reconciliation process) and B (using an automatic claims reconciliation process) in design choice 27 about determining the claims-processing procedure (because, when choosing option B in design choice 3, reconciliation based on the existing fee-for-service infrastructure may no longer be needed to process claims).

### Implications for policy and further research

Although payers often have a tendency to make contracts as complete as possible [42], for these relatively new bundled payment contracts it might often be preferable to leave some room for leniency in case unexpected events occur (design choice 17). The implementation of an innovative contract that is too rigid could, within the complex dynamics of a health care system, come at the expense of the trust of providers [43]. Therefore it may be advisable to begin with a few small scale pilots in which the financial risk for providers is very limited (or even, at first, absent), in which most contract terms are freely negotiable and where there is time to experiment and learn. This gives providers the opportunity to optimize care processes and become confident in using the new payment model, while for payers it will be an opportunity to see what the provider(s) view as challenges. Although such a more open and flexible approach to procurement may be labor-intensive and time-consuming in the short term, it could result in more valuable provider-customized contracts in the long run. Additionally, this can also establish and strengthen the payer-provider trust relationship that is necessary for more extensive forms of health system and payment reform, such as the introduction of regional accountable care organizations.

In addition, in order to increase the framework’s practical applicability, payers should always approach it with a clear vision, scope and goal, because such an attitude will help determine which options will be most relevant to their implementation strategy. To illustrate this point further we briefly describe a general four-step implementation strategy. For example, step 1: start out with a few small pilot contracts in which care services are bundled for one (relatively straightforward) medical condition within just one provider organization (e.g., bundling the relevant DRG’s in a hospital), then step 2: scale up the successful contracts by also including care services of other providers involved in the patient journey (e.g., primary care providers), then step 3: expand with new contracts to other (less straightforward) medical conditions to, eventually, step 4: have bundled payments as the health systems’ dominant payment model. During the first step (i.e., small pilot contracts), for instance, the implementers may choose option B (i.e., a ‘stepped-model approach based on included care’) in design choice 1 (because the pilot includes the care of just one provider), option B (i.e., ‘no alignment agreement with other payers’) in design choice 2 (because alignment agreements with other payers are not yet relevant), and option A (i.e., ‘a retrospective payment strategy’) in design choice 3 (because a retrospective payment strategy is easier to implement), etcetera. During the fourth step (i.e., when bundles became the ‘dominant payment model’), for new bundled payment contracts, the implementers may choose option D (i.e., ‘immediate transition to a full bundle (no stepped-model)’) in design choice 1 (because they may want to abandon the underlying fee-for-service infrastructure completely), option A (i.e., ‘establish an alignment agreement with other payers’) in design choice 2 (because, due to the larger scale, alignment agreements with other payers could become relevant), and option B (i.e., ‘a prospective payment strategy’) in design choice 3 (because a prospective payment strategy may strengthen financial incentives for providers [8]), etcetera. This example indicates that making choices in the design and implementation process of bundled payment contracts becomes more straightforward and logical when the implementers have a clear picture of the goal(s) of the contract.

Further research should focus on identifying, clustering, and structuring the choices and options in the design and implementation process of bundled payment contracts from a provider’s perspective. It should also consider the effect of specific implementation strategies (i.e., specific combinations of design options) on the delivery and organization of health care, the satisfaction of care professionals and patients, transaction costs, and potential side effects (like changing market conditions and ‘gaming’). Future research could also apply and refine this framework using actual contract data and/or explore how procurement strategies differ across health systems with alternative governance and payment arrangements.

Bundled payment contracts introduce a shift in financial risk from payers to providers that will affect the health care system in both its formal aspects (systems, processes, laws, regulations, etc.) as well as in its informal aspects (organizational culture, autonomy, routines, attitudes, etc.). Bundling payments should thus rather not be approached as just another care procurement strategy but as part of a much bigger transition towards a sustainable and value-based health care system. This may eventually also require important political choices that transcend the level of the individual payer (e.g., developing an all-payer database that is safely accessible and user friendly to all relevant stakeholders).

## Conclusion

Our framework provides a structured overview of 33 literature-based design choices and their options (and, where available, the potential impact of choosing between those options) in the procurement process of a bundled payment contract using a payer’s perspective. A better understanding of what these choices and options are can enable payers (and providers) to design and implement this promising payment model in their own setting, so that it can support value-based care delivery for patients.

### Highlights

- While many studies describe individual design choices in the care procurement process of bundled payment contracts, information is fragmented and a structured overview of choices using a payer’s perspective, is missing.
- Through qualitative content analyses of the literature, we extracted 33 design choices with in total 110 policy options and structured them in a comprehensive overview that enables payers to start experimenting with bundled payment contracts.
- This study illustrates how choices in the design and implementation of bundled payment contracts are context-dependent, interchangeable, and interrelated. Therefore, a clear overview of all design choices and options can reduce complexity during the procurement process.

## Supporting information

S1 Appendix Appendix A. (DOCX)

S2 Appendix Appendix B. (DOCX)

S3 Appendix Appendix C. (DOCX)
