# Supplementary material for: Choices and options in the care procurement process of bundled payment contracts: A literature-based overview from a payer’s perspective
Source: PLoS One. 2026 Jun 15;21(6):e0346366. doi: 10.1371/journal.pone.0346366 (PMC13268200; doi:10.1371/journal.pone.0346366)
Supplement: S1 Appendix — (DOCX) [file pone.0346366.s001.docx]

| **Appendix A. References to the original text excerpts** | |
| --- | --- |
|  | |
| **ARTICLE 1: Bushnell, 2015 ^1^** | |
| **Selected Text Excerpt** | **Reference No.** |
| "In the ideal setting, a single source provider will be able to manage all aspects of an episode of care from start to finish. Such a single source would likely be a major health system, with employed primary care physicians and surgeons, hospitals, physical therapy (including inpatient, home health, and outpatient), durable medical equipment, and pharmacy services. In this situation, the health system controls every aspect of the care episode under the bundle and thus can most effectively manage costs and maximize profit. However, in reality most local and regional health care situations do not have a single source provider but are composed of multiple providers for each and every part of the episode of care. In these situations, significant negotiations must occur to get a bundled payment system off the ground. The various participants must determine who does what and, most importantly, who gets paid what. Typically, bundled payments result in a single check from the payer to a designated recipient. Negotiations must determine who that recipient will be and how the money will be distributed downstream to the other participants. This payment determination process presents a major opportunity for both conflict and collaboration, and it may be the single most critical step in the whole process." | 1.01.1 |
|  | 1.01.2 |
|  | 1.01.3 |
| "The number and types of patients who can participate in a bundled system must also be defined. ln some cases, providers may refuse to offer a bundled pricing deal to patients with various comorbidities or other factors that place them at high risk of postoperative complications. Because complications dramatically increase the costs of care and that cost is transferred to the provider in a bundled arrangement, providers will likely not try to include high-risk patients. Patient whose care involves bundled pricing arrangements will likely undergo risk stratification by the provider in the process of setting prices." | 1.02.1 |
|  | 1.02.2 |
| "Although health care episodes are obviously not automobiles, a system based on bundled pricing still requires the creation of protocols or care pathways that direct the provision of services and products. These protocols must be predictive: they follow the same steps and produce the same outcome every time, just like an assembly line. Creating agreement about the contents of a care pathway or protocol is perhaps one of the most difficult parts of developing a bundled pricing system, especially in orthopedic surgery. The orthopedic' clinical literature lacks high-level evidence in support of many common practices, leading to a great deal of variation by surgeon, procedure, and location. The American Academy of Orthopedic Surgeons has created numerous Clinical Practice Guidelines in an attempt to help direct care pathways, but the Guidelines have been widely criticized as confusing and ineffective. Therefore, participants in a bundling agreement must work at a local contractual level to standardize implants, medications, timing, therapy protocols, and other aspects of the clinical experience to make episodes of care more predictable . Without a predictable protocol, pricing becomes impossible." | 1.03.1 |
| "One big challenge for predicting resource use is the rare but real risk of major complications after major orthopedic procedures, wherein the patient may suffer cardiac, pulmonary, or neurological events that require treatment in expensive critical care or intensive care units. Although their occurrence may be low, especially in larger centers, the cost of such complications must be factored into the equation." | 1.04.1 |
| "As the overall health care system moves toward a bundling model, providers must take on several duties traditionally performed by claims administrators - a major issue not only for digital infrastructure but also in terms of the required personnel with appropriate skillsets and experience. A period of dual payment methods (ie, traditional vs bundled) may exist for several years before the new methods are fully integrated, so additional staffing and support may be needed temporarily to handle the double-duty volume." | 1.04a.1 |
|  | 1.04a.2 |
| "However, by grouping all services under a single bundled price, the risk is shifted from the payer to the providers. If costs exceed the bundled price, then the loss belongs to the provider, not to the payer. Such a transfer of risk forces the providers to become much more cognizant of ways to decrease costs and improve efficiency of the overall service in addition to their individual component. Traditional relationships have often made providers feel powerless at times in the game of reimbursement, but new pricing models will result in them becoming much more involved and interactive players in the game. For example, in a bundled arrangement for a total knee arthroplasty, the therapist will care about medication prices, and the brace shop owner will pay attention to average length of hospital stay." | 1.05.1 |
| "Under a fee-for-service system, surgeons are financially rewarded for higher volume, regardless of clinical outcomes. Although quality outcomes tend to drive volumes up for any given provider, this may not always hold true. With no tracking of outcomes or ECV, surgeons can build substantial wealth providing loosely indicated and/or poorly performed procedures. However, with a sharing of risk, physicians are immediately incentivized to provide higher quality at lower cost or suffer financial consequences. Although volume remains a profit driver, it is only quality volume that will pay off under bundling." | 1.06.1 |
|  | 1.06.2 |
| "As bundled payment arrangements increase in prevalence, payers’ incentives will also shift somewhat. Whereas payers traditionally sought out the best price for each individual product, bundled pricing obviously changes that search to one for the best total price. In some cases, the total bundled price may not beat the total of the à la carte prices, at least in the early days of bundling. However, payers will also analyze their hidden costs and downstream costs, such as the time and human resource costs of multiple per-item negotiations with multiple providers vs the cost of a single negotiation with a bundled payment provider. Such savings from alterations of internal logistics and administrative costs will incentivize payers toward bundled pricing for its own sake." | 1.07.1 |
|  | 1.07.2 |
| "Insurance companies may offer discounted premiums or lower deductibles for patients who establish medical relationships with participating providers or institutions. Copayments may be lower at a bundled facility or with a bundled provider. In some cases, patients may even be offered actual cash payments as inducement for choosing to have a surgery done with providers participating in a bundling arrangement." | 1.08.1 |
| "In a traditional market setting, bundled pricing tends to benefit the seller. [...] However, health care markets do not always play by traditional rules. Bundled pricing will decrease the line-item transparency that currently exists, but this may actually drive prices down for the buyer. Payers, not patients, are usually the main driver of price determination under the current model of health care finance. The prices of individual items, now painstakingly analyzed by payers, will no longer matter once cost-containment responsibilities are shifted to the provider. The payer will compare the bundled cost for an episode to its current costs for the episode and then make a payment decision, leaving the line-by-line breakdown to the providers. However, the effect on the consumer may be just the opposite; bundled payments will likely result in increased cost transparency for the patient. Currently, the patient usually has no idea about the actual costs of his or her care. Although information about individual prices may be available now, the consumer has little reason to use that information. Explanation of benefits (EOB) documents are at best limited and confusing, and many patients do not even read them. Because the EOBs may originate from different providers over vastly different time frames, they have little ability to convey meaningful information about total cost to a patient. The cost of the proverbial forest is thus lost in the poorly managed transparency of the trees. Indeed, the average American health care consumer probably focuses only on his or her individual deductible, copay, and out-of-pocket expenses rather than the actual total cost of his or her care. However, in a bundled payment model, it will be relatively easy for a patient to obtain information about the single bundled price for his or her care. Increased transparency of the total cost, especially if linked somehow to the patient’s share of that cost, has the potential to significantly change patient behavior." | 1.09.1 |
|  | 1.09.2 |
|  | 1.09.3 |
|  | 1.09.4 |
| "Larger hospitals and health systems will likely be able to bundle services and products more effectively and therefore provide them at a total cost that is lower than the incremental cost of all  parts of the bundle. This competitive advantage will likely result in increased clinical volumes for the larger entities. This could be seen as legally problematic because it deprives smaller entities of the volume needed to maintain economy of scale for their nonbundled product, thus eliminating the check on the market power of the larger entity. Such high-volume bundling may deter competitors from even entering the market in the first place. In many markets, such practices could be interpreted as essentially monopolistic and harmful to consumers." | 1.10.1 |

| **ARTICLE 2: Hussey et al., 2011 ^2^** | |
| --- | --- |
| **Selected Text Excerpt** | **Reference No.** |
| "The payment rates for these bundles are called “evidence-informed case rates.” PROMETHEUS has defined twenty-one bundles that include chronic medical conditions such as diabetes, acute medical conditions such as acute myocardial infarction, and procedures such as hip replacement. Several factors complicate this approach to payment. First, recommended services are only a portion of the cost of an episode of care. For example, a congestive heart failure episode may include recommended prescription drugs and diagnostic tests as well as other services, such as hospitalization for a preventable exacerbation of the condition." | 2.01.1 |
| "PROMETHEUS distinguishes two key sources of variation in the total cost of health care: probability risk and technical risk. Probability risk, the classic form of insurance risk, is caused by the likelihood that a random event will occur to a patient as a result of his or her genes, health status, and any external event not controllable by the provider, such as whether or not the patient contracts a virus or breaks a leg. Technical risk is related to “care production.” It is controllable by the provider and is responsive to the provider’s clinical skills and the systems of care he or she uses. Conceptually, the PROMETHEUS model is designed with the goal of transferring financial responsibility for events related to technical risk to providers, because that is what is in their control, while insurers retain responsibility for probability risk, because that is a traditional function of insurance. Failure to control technical risk leads to what PROMETHEUS calls “potentially avoidable complications,” which means that a provider is expected to be able to avoid them with adequate health systems, planning, and training. Examples of potentially avoidable complications in patients admitted to a hospital for acute myocardial infarction include urinary tract infections, blood vessel inflammation (phlebitis), and strokes under certain conditions. For patients with chronic conditions, potentially avoidable complications include any hospitalization related to the chronic condition. PROMETHEUS developed a methodology by which pilot sites can set case rates on the basis of historical service use and cost patterns, particularly the cost of potentially avoidable complications. The price-setting methodology is based on the concept of a “warranty” that the costs of potentially avoidable complications will not exceed an agreed-upon amount." | 2.02.1 |
|  | 2.02.2 |
|  | 2.02.3 |
| "A bundled payment system requires a definition of the services that are included in and excluded from a bundle. Operationalizing the bundled payment system requires modifying insurers’ claims-processing procedures in order to identify services that will be subject to bundled payment rather than fee-for- service payment and modifying payments to providers accordingly. The Health Care Incentives Improvement Institute developed the PROMETHEUS Engine, an accounting tool that analyzes each insurance claim submitted for reimbursement by a participating provider; determines whether the service covered by the claim is part of a bundle; and, if it is, adds the payment amount to a running total budget for the case rate. In addition, the institute developed analytic software that pilot sites could use to analyze their historical claims data for cost and utilization patterns in bundles of care." | 2.03.1 |
| "Bundled payment systems may use different incentive structures with varying levels of financial risk for providers and payers. The challenge is reaching agreement between payers and providers on an incentive structure that results in an acceptable and manageable division of financial risk while rewarding providers for redesigning care. The Health Care Incentives Improvement Institute has suggested a variety of payment method options to the pilot sites that may be phased in to increase providers’ risk over time." | 2.04.1 |
| "Bundled payment often requires new arrangements for assigning accountability because an episode of care may involve several providers, including physicians and hospitals. First, methods are needed to identify patients who have begun an episode subject to bundled payment and to “attribute” responsibility for their episode of care to a provider organization. The providers furnishing services included in the bundle then need to collaborate to manage patient care. Finally, the provider organizations receiving the bundled payment must agree to a mechanism to share the payment." | 2.05.1 |
| "A key challenge is getting providers to buy in to the bundling methodology and then motivating them to make the changes in delivery needed to reduce costs and improve quality. Providers are likely to be wary about changes perceived as designed to cut costs without regard to improving the quality of care. They often associate bundled payment with unpopular previous payment methods such as capitation, under which a provider receives payment based on the number of patients he or she is responsible for, usually in a health maintenance organization setting, whether or not those patients receive any services." | 2.06.1 |
| Pilot sites have had difficulty applying case-rate definitions to their own data, particularly in real time. | 2.09a.1 |
| "Interviewees at all three sites also reported that they viewed bundled payment as a way to focus on the best way to provide high-quality, low-cost care—a primary goal of these organizations. In addition, all three sites intended to use their participation in PROMETHEUS as a strategy to increase market share. Interviewees at two sites stressed that strong organizational commitment, including clear support from senior management, is a prerequisite to successful implementation of PROMETHEUS." | 2.07.1 |
|  | 2.07.2 |
| "The decision to focus on procedures was described as a trade-off between ease of implementation and potential for cost savings. Bundled payment was viewed as easier to implement for procedures than for chronic medical conditions because procedures follow a more clearly defined clinical pathway and involve fewer providers. On the other hand, bundled payment for chronic medical conditions was viewed as offering stronger potential for improvements in care delivery because potentially avoidable complications were found to be much more common and costly in chronic condition bundles compared to procedures." | 2.08.1 |
|  | 2.08.2 |
| "Bundled payment is complex and must build on existing complex health care systems. As implemented, PROMETHEUS builds on fee-for- service claims infrastructure and thus adds to the complexity of existing payment systems. Services that are part of a clinical episode, and thus subject to bundled payment, are identified using the information about the patient’s diagnoses and services that providers report on fee-for-service insurance claims. The same information is also used to classify each service as either typical care or a potentially avoidable complication. The decision rules that determine whether specific services are part of a bundle and, if so, whether they constitute typical care or potentially avoidable complications are complex and depend on the quality of the information that providers include on claims, which are not designed with the needs of a bundled payment system in mind." | 2.09.1 |
|  | 2.09.2 |
| "Difficulties also arose because participants in the pilots encountered what was, in effect, a critical language barrier. Part of the complexity of PROMETHEUS is that it contains its own lexicon of terms such as “typical costs,” “potentially avoidable complications,” and “ECRs” (evidence- informed case rates) that are not commonly understood. Mastering the language takes time, and pilot sites found that different stakeholders interpret terms in different ways. Interestingly, what seems like a mere semantic concern contributed greatly to implementation delays." | 2.11.1 |
| "An additional challenge has been modifying complex insurance claims processing procedures to identify services that are part of a bundle. A key technical issue is that claims are often modified or rejected during adjudication and then may be resubmitted. The case-rate budget must track these iterations and include only final, approved claims." | 2.10.1 |
| "Interviewees at all three pilot sites explained that the language used in PROMETHEUS is largely oriented toward broad conceptual categories, whereas physicians are accustomed to thinking in terms of specific, concrete cases. One result was that it was difficult for physicians to translate retrospective analyses of typical and potentially avoidable costs from program data reports into their everyday clinical experience. In addition, interviewees at two sites reported that physicians had disagreed with some of the potentially avoidable complication designations. Although these designations could be changed, interviewees reported a more general semantic issue related to potentially avoidable complications: Physicians tend to view the tag “potentially avoidable” as meaning “always preventable” and implying a failure on their part. This contributed to doubt among physicians at the pilot sites about the validity of “potentially avoidable complication” designations. Moreover, several interviewees at one pilot site expressed strong skepticism about a suggestion made in PROMETHEUS promotional materials that case rates should be based on the assumption that providers could reduce service designated in PROMETHEUS bundles as “potentially avoidable complications” by 50 percent. They noted that the evidence to back up this claim was thin or unavailable. Interviewees speculated that “potentially avoidable complication” designations are likely to come under increasing scrutiny as more pilots are implemented and the designations are vetted by a larger and more varied set of health care providers." | 2.11a.1 |
| "In the shared savings model, each provider’s actual patient care costs are periodically compared retrospectively to the agreed-upon threshold. This comparison determines whether the provider is eligible for a shared savings bonus payment—if actual costs were lower than the threshold and, if so, how much. However, shared savings has turned out to be more difficult to implement than expected. Interviewees at two sites reported that neither payers nor providers were eager to set aside funds from which to make the bonus payments. In addition, some payers did not accept the idea that they should share any savings. They expressed skepticism that the “savings” represented real long-term improvement and not natural variation in costs." | 2.12.1 |
| "All three sites are using electronic health records as a crucial component of their strategies for care redesign. However, the sites have found that their record systems lack key capabilities that would enable more effective care redesign. One commonly cited barrier was the lack of comprehensive data exchange between outpatient and inpatient settings, even within a single health system." | 2.13.1 |
| "The findings do not provide support for discarding bundled payment in favor of alternative payment methods. Many of the lessons learned from the pilot sites—such as the difficulty of adding complexity to an already complex system, agreeing to specific payment incentives, and redesigning care before large shifts in payment— are likely to apply to other payment and delivery reforms, including accountable care organizations and patient-centered medical homes." | 2.14.1 |

| **ARTICLE 3: Miller, 2012 ^3^** | |
| --- | --- |
| **Selected Text Excerpt** | **Reference No.** |
| "If better coordination of a patient’s care can avoid an emergency room visit or hospital admission, the hospital will lose all of the revenue for that visit or admission, but it will still have to cover the costs of having the emergency room or hospital bed available. Giving the hospital a bonus or shared savings payment for lower admission rates can still penalize the hospital, since the portion of the lost revenues offset through the shared savings payment may be less than the fixed costs the hospital must continue to cover. Having two or more providers participating in a shared savings arrangement creates a version of the prisoner’s dilemma: if provider #1 makes a good faith effort to reduce unnecessary services but provider #2 does not, provider #2 would “win” by maintaining its own fee revenues while also potentially receiving part of the savings generated by provider #1. If provider #2 increases its volume of services, it would receive more revenue and also thwart the opportunity for provider #1 to receive any shared savings to offset the revenue it lost." | 3.01.1 |
| "Where these approaches have been used, both providers and payers have benefited. For example, in the Medicare Acute Care Episode (ACE) Demonstration, which “bundles” physician and hospital payments (i.e., it makes a single payment to both providers, rather than separate payments to each), Medicare has saved money, physicians have received higher payments, hospitals have been able to reduce their costs and improve their operating margins, and patients have received better care. The positive results from this program led the CMS Innovation Center to create its Bundled Payments Initiative, which will both allow additional providers to participate in the bundling approach used in the ACE Demonstration and allow providers to accept full episode payments for a variety of conditions.11 This win-win-win approach – lower spending for payers, better care for patients, and better margins for providers – is only feasible with the types of significant payment re-forms described above, not with minor tweaks to the fee-for-service payment system." | 3.01a.1 |
| "If payment reforms are designed properly, there will be no need to mandate them; many providers will voluntarily accept a payment system that gives them the flexibility to deliver the best care to their patients and rewards them for high-quality care at an affordable cost without putting them at risk for costs they cannot control." | 3.02.1 |
| "There are two key ways to structure payments so that they give providers only the types of financial risk they can manage: 1. Separating Insurance Risk and Performance Risk. First, a payment system should be structured so the payer retains the “insurance risk” (i.e., the risk of whether a patient will develop an expensive health condition) and the provider accepts the “performance risk” (i.e., the risk of higher costs from delivering unnecessary ser-vices, delivering services inefficiently, or commit-ting errors in diagnosis or treatment of a particular condition).17 Many of the problems with man-aged care in the 1990s arose because traditional capitation payment systems transferred both insurance risk and performance risk to providers, causing bankruptcies when providers took on care of many sick patients without any increase in payment. 2. Focusing on Costs That a Provider Can Control. Second, a payment system should give a healthcare provider accountability for the types of services and costs that the provider can control or significantly influence, but not for services and costs over which the provider has little or no influence. For example, primary care physicians are in a much better position to determine the appropriateness of services they prescribe than health plans are, so building accountability for utilization of prescribed services into physician payment is better than trying to control utilization through prior authorization and utilization review programs operated by health plans. On the other hand, a payment reform system that only gives primary care physicians a bonus if there are reductions in the total cost of all services their patients receive from all providers goes too far in shifting accountability, since primary care physicians do not control all of the factors that drive the total cost of care for their patients. (For example, assume that a primary care physician is able to significantly reduce the rate at which his or her chronic disease patients are admitted to the hospital for exacerbations of their chronic condition; if the subset of patients who are still admitted to the hospital develop serious infections or complications, total costs might increase, even though the primary care physician had been successful in controlling the aspect of utilization that he or she could influence.)" | 3.03.1 |
|  | 3.03.2 |
| "Some payers have raised concerns about using risk adjustment as part of a payment system because a patient’s risk score tends to increase as soon as they become part of a risk-adjusted payment system, and this can cause overall spending to increase rather than decrease.20 This happens because, under fee-for-service payment, the diagnosis codes used for risk adjustment are only recorded when a related claim for treatment is filed; as a result, many health conditions are not recorded in health plans’ claims data systems (particularly if patients have recently changed health plans). However, under a risk-adjusted payment system, the provider has an incentive to do complete coding of diagnoses, not just to ensure accurate payment, but to ensure that all of the patient’s health conditions are being managed in a comprehensive and coordinated way. Rather than eliminating risk adjustment entirely to avoid this artificial increase in risk scores (which could thereby discourage providers from taking on sicker patients), risk adjustment systems should be modified so that both the baseline risk score and current risk score are changed when a patient’s pre-existing condition is identified and documented. Broader use of electronic health records will help to address this problem by enabling risk adjustment to be based on complete clinical data on the patient’s past and current patient health conditions, not just on data recorded to support recent claims for payment to a particular health plan." | 3.04.1 |
|  | 3.04.2 |
|  | 3.04.3 |
| "Current risk adjustment systems also need to be improved so they do not penalize providers for keeping their patients well. A patient’s risk score is typically based on the health problems that a patient has today, not on how those problems have changed as a result of the health provider’s care. So, for example, if a physician helps a patient lose weight or stop smoking, the patient’s risk score would decrease, and as a result, under a risk-adjusted payment system, the physician would receive a lower payment than if the patient had remained unhealthy, thereby penalizing the physician for a successful health improvement effort. Improved risk adjustment systems that capture such changes over time will be needed, particularly if more providers and payers sign multi-year contracts to manage healthcare cost and quality" | 3.05.1 |
| "Current risk adjustment systems also need to be improved so they do not penalize providers for keeping their patients well. A patient’s risk score is typically based on the health problems that a patient has today, not on how those problems have changed as a result of the health provider’s care. So, for example, if a physician helps a patient lose weight or stop smoking, the patient’s risk score would decrease, and as a result, under a risk-adjusted payment system, the physician would receive a lower payment than if the patient had remained unhealthy, thereby penalizing the physician for a successful health improvement effort. Improved risk adjustment systems that capture such changes over time will be needed, particularly if more providers and payers sign multi-year contracts to manage healthcare cost and quality." | 3.05.2 |
| "At best, risk adjustment is only a partial solution; no formula could ever be 100% accurate in predicting legitimate variations in costs, simply because of the myriad factors that can affect patient costs and outcomes. To adequately protect both providers and patients, risk adjustment should be supplemented with risk limits, such as: Outlier payments to cover unusually high costs for specific patients. “Risk corridors” that require payers to provide additional payments to providers when the total cost of treating a group of patients significantly exceeds the agreed-to payment level. The sizes and cost-sharing parameters for these risk corridors could vary from provider to provider, since larger providers will be better able to manage variation in costs, and the parameters could also be changed over time as providers become more experienced in managing costs. In some cases, it is clear that certain kinds of costs cannot reasonably be controlled by a provider, and rather than using risk adjustment formulas or other complex calculations to adjust for this, these costs (or the situations that lead to them) should simply be excluded from accountability altogether. For example, the costs associated with patients who are seriously injured in accidents could simply be excluded entirely from a global payment model for a small group of physicians, and be paid for separately on an episode-of-care basis or under traditional fee-for-service." ... "One of the arguments made in favor of the retrospective statistical attribution system is that it may reduce the possibility that physicians will “cherry-pick” their patients, i.e., avoid patients who are sick or likely to require expensive ser-vices. However, if the payment system includes appropriate types of risk adjustment, risk limits, risk exclusions, and provisions for adjustments as described under Barrier #2, physicians would actually have an incentive to care for sicker patients, because the opportunities to generate savings would be greater." | 3.06.1 |
|  | 3.06.3 |
|  | 3.06.2 |
|  | 3.06.3 |
| "Multi-year contracts between payers and providers pro-vide a better opportunity for providers to make changes in care delivery that take time to implement and to reap returns on investments in preventive care and infrastructure, and they give payers greater ability to control the trend in health-care costs. However, the longer the contract, the greater the potential for unexpected events to occur, the greater the difficulty of building appropriate protections into a contract to deal with those unexpected events, and the greater the reluctance providers and payers will have to sign. A solution to this is simply to acknowledge that unexpected events may occur and to provide for opportunities to make adjustments in the contract to deal with them. Of course, the party which is disadvantaged by the unexpected event will be more interested in making an adjustment than the party which benefits from it, so the contract could pro-vide for having a neutral arbitrator resolve any disagreements." | 3.07.1 |
|  | 3.07.2 |
|  | 3.07.3 |
|  | 3.07.4 |
|  | 3.07.5 |
| "Clearly, if payment systems are changed to reward value rather than volume, the compensation of in-dividual physicians and other providers will also need to be changed to align with the structure of the new payment system, rather than with fee-for-service payment. Rather than primarily basing compensation on “productivity,” physicians will need to be compensated based on factors such as quality, teamwork, and overall cost-effectiveness that will determine the provider organization’s success under the new payment system." | 3.02a.1 |
| "Most of the literature on payment reform has focused on how to change the method of payment, but there has been relatively little attention to how to set an appropriate payment amount (i.e., the price). Regardless of how good the payment method is, if the payment amount is too low, providers will be unable to deliver quality care, and if the payment amount is too high, there will be no savings for purchasers/payers and little incentive for providers to reduce costs." | 3.03a.1 |
|  | 3.03a.2 |
| "Even if providers have access to claims data, however, most would not have the analytic capacity to assemble and analyze large claims databases, particularly if the data come from multiple payers. Also, there could be privacy concerns about giving providers patient-identifiable data about all services from other providers in order to find and combine multiple claims records for their own patients. The best solution is for all payers to contribute their data to a multi-payer database managed by a multi-stakeholder Regional Health Improvement Collaborative that can help providers analyze the data while protecting patient privacy." | 3.08.1 |
|  | 3.08.2 |
|  | 3.08.3 |
| "Using these retrospective statistical attribution rules to assign patients to providers means that neither the provider nor the patient knows they are part of the new payment system until after the care is delivered, potentially a year a more later. If providers and payers only find out retrospectively that they are in a new payment system, it will be difficult for them to work together prospectively to change care and prevent un-necessary costs from occurring." | 3.09.1 |
| "..in various medical home programs, the primary care provider receives an additional, non-visit-based payment for each patient who is attributed to the PCP using these retrospective statistical attribution rules. However, if a patient does not make a billable visit to their PCP during the specified time period, the patient will not be attributed to that PCP (or any other physician), which means there will be no change in payment to support better care for that patient. This is not a small problem. In one study of Medicare patients using these types of attribution rules, 15% of the patients could not be attributed to a primary care provider, and 6% could not be attributed to any physician.41 The unattributed percentage will likely be much greater for patients on commercial insurance, because when a patient switches insurance, even if they have a consistent PCP, it may take months or years for the new payer’s claims data to justify attributing the patient to that PCP. If there are multiple insurance companies in a particular market, and if they are competing aggressively for business, it is likely that a large percentage of a PCP’s patients will not be attributed to them by the payers, because the payers will not have a sufficiently long claims history on those patients to determine who their “real” PCP is. This is only part of the problem, however. The types of care changes the non-visit payment is designed to encourage can actually cause the physician to stop receiving the new payment. This is because attribution rules are typically based on the number of visits a patient had to primary care providers. Physicians who redesign their practices to reduce the emphasis on office visits for healthy patients in favor of phone calls and emails, while providing longer office visits for more complex patients, will be harmed financially under this system, since they will not only lose fee revenue by having fewer office visits, but they may also not receive any additional payment for the patients who do not have the recent office visits that are required to trigger the attribution calculation. In programs with a shared savings component, a provider is expected to reduce the costs of care for the patients who are attributed to that provider. However, if the attribution rules assign patients to the provider whose care the provider cannot influence, the provider can be inappropriately penalized if costs for those patients increase (or inappropriately rewarded if costs decrease). If the attribution rules fail to assign a patient even though the provider was responsible for improving the efficiency of care for that patient, the provider would fail to receive the bonus payment that they deserved." | 3.04a.1 |
| "“Last-dollar” cost-sharing for expensive services offered by multiple providers. A growing number of studies have found that some providers are paid five to ten times as much as others to deliver procedures such as cardiac and orthopedic surgeries and labor and delivery.46 Yet even with high copayments, co-insurance, or deductibles, most patients will pay the same amount regardless of whether they choose the most expensive or least expensive provider of a high-cost service, which in turn gives little incentive to the providers to lower their prices.47 Instead of requiring consumers to pay a portion of the “first dollar” that the provider charges for each individual service (through a co-payment, co-insurance, or deductible), consumers could be charged all or part of the “last dollar,” i.e., the difference in total prices between higher-cost and lower-cost providers.48 Some employers and health plans are beginning to implement this through “reference pricing” of services, i.e., defining the maximum amount the health plan will pay for a service based on the existence of one or more quality providers who will deliver the service for that price, and then requiring the patient to pay the difference in price if they choose a higher-priced provider.49 Episode-of-care payment and global payment systems will facilitate this approach by defining a true “total price” for services, avoiding the possibility that a provider might offer an unusually low price for the basic procedure to win the business but then make up the loss in revenue by delivering additional services." | 3.10.1 |
| "However, just as more spending in healthcare doesn’t necessarily result in better quality care, merely tying payment to a large number of quality measures doesn’t necessarily result in better quality care, either. Requiring healthcare providers to measure, report, and improve on a large number of quality measures can actually be a deterrent to provider participation in new payment models, particularly if the quality measures demand changes that go far beyond the re-sources and flexibility provided in the payment system. For example, the regulations that were initially proposed for the Medicare Shared Savings Program were widely criticized for including 65 different measures of quality, despite providing no change in the underlying fee for service structure to support better care; in response, CMS reduced the list to 33 measures in the final regulations. Similar to the discussion under Barrier #2 regarding costs that providers can and cannot control, physicians and hospitals will likely resist participating in a payment model which holds them accountable for aspects of quality they cannot control. For example, many hospitals have objected to having Medicare and other payers impose financial penalties on them for high rates of readmissions for chronic disease patients when there is considerable evidence that such readmissions result from poor primary care in the community as much or more than poor care in the hospital. Physicians will be reluctant to take accountability for quality measures that require patient adherence to care plans if the patients’ health benefit structures make it difficult for them to adhere.." | 3.06a.1 |
|  | 306a.2 |
| Similar to the discussion under Barrier #2 regarding costs that providers can and cannot control, physicians and hospitals will likely resist participating in a payment model which holds them accountable for aspects of quality they cannot control. For example, many hospitals have objected to having Medicare and other payers impose financial penalties on them for high rates of readmissions for chronic disease patients when there is considerable evidence that such readmissions result from poor primary care in the community as much or more than poor care in the hospital. Physicians will be reluctant to take accountability for quality measures that require patient adherence to care plans if the patients’ health benefit structures make it difficult for them to adhere, as discussed in more detail under Barrier #5. | 311.1 |
| "..quality measures for individual physicians tend to be unreliable because of the small numbers of patients involved, but as individual physicians join together to manage new payment models, measures can be calculated and reported for all of the participating physicians." | 3.12.1 |
| "True payment reforms will require more significant effort by payers. However, in markets with multiple payers, there is an incentive for each individual health insurer to be a “free rider,” i.e., to avoid the costs of implementing payment reforms while retaining all of the savings generated by providers in response to payment reforms implemented by other health plans. (Since physicians who improve the way they deliver care will do so for all of their patients, care improvements stimulated by one health plan will benefit the members of other plans, too.)." | 3.13.1 |
| "However, even if all payers move away from fee-for-service, the most likely outcome is that they will do so in different ways. If each payer designs payment reforms on its own, each payer may choose to include different services in an episode or global payment, use different measures of quality, use different systems for risk adjustment, etc. At best, these differences will cause providers to face significantly higher administrative costs; at worst, they will create conflicting incentives that could impede improvements in care or deter providers from participating at all. There are several reasons why it is challenging for commercial health plans to implement common payment reforms: 1. Concerns about antitrust law violations make it difficult for health plans to discuss or reach agreement on a common approach to payment. 2. Many health plans pay for patients located in multiple geographic markets, and they find it more efficient to use the same payment system in all of their markets, even if that results in lack of alignment with other payers in any particular market. 3. Since employers, state Medicaid agencies, and other healthcare purchasers typically demand that health plans compete for their insurance business, health plans may fear that employers will penalize them for not being “innovative” if they simply use the same payment models as other payers." | 3.07a.1 |
| "..even if all payers move away from fee-for-service, the most likely outcome is that they will do so in different ways. If each payer designs payment reforms on its own, each payer may choose to include different services in an episode or global payment, use different measures of quality, use different systems for risk adjustment, etc. At best, these differences will cause providers to face significantly higher administrative costs; at worst, they will create conflicting incentives that could impede improvements in care or deter providers from participating at all. There are several reasons why it is challenging for commercial health plans to implement common payment reforms: 1. Concerns about antitrust law violations make it difficult for health plans to discuss or reach agreement on a common approach to payment. 2. Many health plans pay for patients located in multiple geographic markets, and they find it more efficient to use the same payment system in all of their markets, even if that results in lack of alignment with other payers in any particular market. 3. Since employers, state Medicaid agencies, and other healthcare purchasers typically demand that health plans compete for their insurance business, health plans may fear that employers will penalize them for not being “innovative” if they simply use the same payment models as other payers." | 3.08a.1 |
| Employers and other purchasers should commit to having payment reforms aligned within regions, and support reasonable variations across regions. The savings from better healthcare will (or at least should) go back to the ultimate purchasers of care – employers, state Medicaid agencies, and individual patients – and so they must take a lead role in demanding that their health plans implement appropriately designed payment systems in an aligned way. Once providers show purchasers the opportunities for savings from improved care and the need for payment changes to support that, employers will have a strong incentive to demand rapid action to implement payment reforms. If health plans refuse to implement new payment systems or if they do so in an unaligned way, purchasers should switch to health plans which are willing to align. Since the biggest employers in most com-munities are hospitals and health systems, these providers can lead the way by using new payment models for their own employees; indeed, this would give health systems greater ability to ensure that payment structures are designed in a way that avoids the overly negative impacts on hospitals described in Barrier #8. National employers need to recognize that different payment changes may be needed in different regions, given the differences in delivery structures and the differences in the factors driving healthcare costs across regions; while a uniform national approach might be desirable for such employers, resisting participation in desirable payment reforms simply because they are not being implemented in all regions will deny employers savings in the regions that are willing to make changes and thereby also slow the pace of reform nationally. | 3.09a.1 |
| "A new payment system will fail if it bankrupts hospitals or forces them to find ways to increase utilization simply to stay afloat. Both payers and hospitals will need to make changes in order to ensure that payment reforms can successfully support high quality, affordable hospital care:  1. Hospitals must reduce their costs. Hospitals will need to aggressively look for ways to reduce their fixed costs and to improve efficiencies using the kinds of techniques that systems like Thedacare,66 Virginia Mason,67 and Intermountain Healthcare68 have pioneered. Hospitals can benefit from technical assistance in redesigning the way they deliver care, such as through the types of programs operated by the Pittsburgh Regional Health Initiative69 and the Iowa Healthcare Collaborative.70 Bundled payments which align incentives for both physicians and hospitals can help hospitals reduce their costs, as described in more detail under Barrier #1, particularly if the legal issues described under Barrier #3 are addressed. In contrast, consolidating hospitals in order to reduce costs should be a last resort. Although in theory, consolidating hospitals can reduce duplication and increase efficiencies, in practice it has resulted in higher prices, particularly when neighboring hospitals are consolidated.71 If payment reforms are to be successful, they should encourage greater competition among hospitals, not less. 2. Medicare and health plans may need to pay more for some hospital services. Payers may need to increase the amount they pay per admission or procedure to reflect the fact that a hospital’s unit costs will be higher with lower volumes. For example, the cost of an individual surgery will depend on the number of surgeries done, since some of the cost of surgery is variable (e.g., the cost of a joint implant) and some is fixed (e.g., the cost of the surgery suite), so if the number of surgeries decreases, the cost per surgery will likely increase, at least in the short run. It is important to recognize that total spending can still be reduced, even with higher payments per admission, if admissions are being reduced. However, hospitals will need to be more transparent about their cost structures. If a hospital seeks to increase prices following a reduction in utilization, purchasers and payers will need to know if that is a legitimate recalculation of the average cost of care in response to a lower level of utilization after all possible efficiencies have been implemented, or merely a monopolistic effort to replace lost revenue. | 3.14.1 |
|  | 3.14.2 |
| "However, many laws and policies make it difficult for small providers to work together without consolidating. For example, under current antitrust law, if two physicians try to contract jointly with a payer using a single price, they are viewed as having committed a per se violation of prohibitions on price fixing. Yet if those two physicians abandon their independent practices and join the payroll of a hospital which has a single price for the services delivered by all of its physicians, there would be no antitrust violation at all.  Rather than promoting competition, current antitrust policies may unintentionally encourage the creation of large providers at a time when there is growing evidence that large health systems are a major reason why healthcare costs are increasing. One study found that in more concentrated hospital markets, prices were 13%-25% higher for a range of cardiac and orthopedic procedures." | 3.15.1 |
| "Although state governments will be playing an increasingly more central role in healthcare reform in the future, partly as a result of the programs in the Affordable Care Act, they cannot be effective substitutes for the roles that multistakeholder Regional Health Improvement Collaboratives play. The regulatory powers and financial resources of state governments give them some unique strengths, such as the ability to mandate the submission of quality and cost data by providers and payers and the ability to provide anti-trust safe harbors to help establish multi-payer payment reforms and help independent providers coordinate their services. However, it is difficult for state governments to support multi-year healthcare transformation efforts when changes in state administrations and changes in fiscal priorities occur, and it is difficult for states to balance regulatory enforcement powers with programs to facilitate provider improvement. In contrast, the independence and stakeholder governance of Regional Health Improvement Collaboratives provide them with greater ability to support multi-year transformation efforts and to do so in a way that can be adapted to the unique needs of individual geographic regions. Consequently, the greatest success in healthcare transformation will likely come from strong partnerships between state governments and Regional Health Improvement Collaboratives." | 3.16.1 |

| **ARTICLE 4: Pham et al., 2010 ^4^** | |
| --- | --- |
| **Selected Text Excerpt** | **Reference No.** |
| "Research+B95:C124ers also have developed episode-grouping methods for a limited number of conditions as part of the PROMETHEUS Payment model, which is using episodebased payments in several pilot sites. PROMETHEUS relies heavily on accepted clinical practice guidelines for care of a specific condition, such as a heart attack, and “builds up” the expected costs of recommended services. A substantial advantage is the clinical face validity that comes from defining “good care” and specifying the attendant services, but maintenance over time as standards of care change could require resource-intensive updates to the calculations." ... " The groupers’ underlying algorithms also are not well understood by policy makers and providers given their proprietary nature." | 4.01a.1 |
|  | 4.01b.1 |
|  | 4.01c.1 |
| "Ideally, sound clinical logic should also underlie episode definitions. That is, when actual patterns of service delivery are closely associated with the severity and progression of a disease, then claims or encounter data can capture care for a specific condition—with the input of experienced clinicians— and payers can more confidently link financial incentives to those episodes with the expectation that providers could improve care for those episodes." | 4.02.1 |
| "Policy makers also must consider how easily or appropriately episodes can be attributed to providers for payment and accountability purposes. Some types of episodes may involve too much variation in the types of providers involved in treating different aspects of the underlying conditions. For example, it is more difficult to predict the relative involvement of primary care physicians, different subspecialists, hospitals and post-acute care facilities in an episode of heart attack than in an episode of cataract care. In the less predictable cases, it may be difficult to assign clear responsibility for the episode to a small enough number of providers to keep payment approaches simple and transparent." | 4.03.1 |
| "From a provider’s vantage point, the method for selecting and specifying episodes would also take into account how understandable episodes are to providers. That is, providers should be able to recognize when an episode has begun and understand and reasonably predict the full range of services that a patient might need during the course of a typical episode of that type. These might include both services delivered by that provider and services from other providers. Such transparency and predictability would help ensure that providers can respond appropriately to episode-based payment incentives by enhancing care coordination." | 4.03a.1 |
|  | 4.03a.1.1 |
| "There are several ways that payers could calculate and update base payment rates for episodes of care. In general, payers would establish a different base payment rate for each particular episode type—pneumonia vs. hip fracture, for example—reflecting the varying costs incurred by providers in treating these conditions. Payment rates based on historical costs. Payers could opt to use historical data on service use and costs per service for particular conditions. For example, commercial episode groupers calculate efficiency scores based on comparisons of a provider’s actual per-episode costs with expected costs based on costs for the median provider—the provider whose performance is in the middle of the cost spectrum across peers, representing the 50th percentile. Alternatively, the base rate might be set at lower or higher points along the prevailing cost distribution. Selecting a higher benchmark or base rate, such as the 75th percentile, would encourage more providers to participate—a potentially important advantage in the early phase of a voluntary episode-based payment program. In contrast, payers might opt for a lower benchmark or base rate, such as the 25th percentile, if they believe there is significant room for improved efficiency. The main advantage of using historical costs is operational ease and the potential for payers to address a broad range of episode types. The main disadvantage is that relative benchmarks do not reflect the ideal patient care for a given episode and, therefore, lack clinical face validity with providers. Payment rates based on external cost benchmarks. A related decision is whether to use internal or external cost benchmarks to establish payment rates. Payments based on internal data—for example, data covering the patients enrolled in a given episode-based payment program with a specific payer and the participating providers in that program—have the advantage of reflecting the particular health care needs of the population and local medical standards. Payments based on external data—such as data from a population that includes patients not enrolled with that payer or its episode-based payment program—avoid grading providers on the curve by holding them to higher standards. Other advantages of this approach include providing the same fixed standard rather than comparing providers to one another, thereby minimizing the chances that payments would fall to ever-lower levels over time as the average efficiency performance of participating providers improve. Payment rates based on guideline-based standards. Another approach, similar in some respects to external cost benchmarks, is to establish payment rates in a bottom up fashion, based on identifying what a particular type of episode, such as a hip fracture, would cost if providers strictly delivered only recommended care. These normative approaches largely ignore actual or typical patterns of service utilization and instead focus on establishing payment rates based on the costs associated with best practices or well-accepted clinical guidelines. Depending on the circumstances, these rates might be higher or lower than empirically based rates. The PROMETHEUS Payment model is one well developed example, where “evidence-informed case rates” are set based on the resources required to provide recommended care outlined in well-accepted clinical guidelines.15 The main advantage of this approach is that it sets a high and clinically valid standard for efficiency performance, which would maximize face validity from providers’ perspectives and reassure patients that efficiency improvements would not jeopardize their receiving appropriate care." | 4.03a.2 |
|  | 4.03a.2.1 |
|  | 4.03a.3 |
|  | 4.03a.4 |
|  | 4.03a.4.1 |
|  | 4.03a.5 |
| "The main disadvantage of retrospectively identifying providers is that responsibility for improving coordination and efficiency could be diffused among many providers that are not completely aware of who else is providing care to the patient and, thus, would likely result in only modest care delivery improvements. For example, an orthopedic surgeon would certainly know what hospital her hip fracture patient is in, but a primary care physician might not be aware of which neurologist her low back-pain patient self-referred to. In the first example, if the payer supported the orthopedic surgeon with historical data on costs per episode, the surgeon could make more-informed choices about directing the patient to a particular hospital and rehabilitation facility. But in many cases, payers will not be able to determine which providers are responsible for a given episode until long after the relevant services have been delivered." | 4.03a.6 |
| "Limiting attribution to claims data would be easier for payers to implement and lower the risk that providers would engage in favorable selection of less-costly patients but would offer less flexibility for payers to tailor attribution to specific clinical scenarios or to pay providers prospectively. Relying on claims data for attribution may also not reflect actual care relationships accurately, which might result in the episode-based payment program having less face validity with patients and providers. Alternatively, payers could gather additional data, such as providers identifying which patients they treat, or patients identifying which provider they perceive as the responsible provider. This approach may be particularly appropriate if attribution is done prospectively." | 4.03a.7 |
|  | 4.03a.7.1 |
|  | 4.03a.7.2 |
|  | 4.03a.8 |
| "As payers offer episode-based payments for a growing number of episode types, providers may naturally coalesce to assume joint responsibility for related types of episodes. For example, if payers developed episode-based payments for different types of cardiac care episodes, such as cardiac arrhythmias, heart attacks and CABG surgeries, groups of cardiologists, cardiac surgeons, hospitals and general internists could collaborate in applying to receive bundled payments for those episodes. Once episode-based payments become available for a wide range of episodes, payers could consider converting to capitated payments for each accountable provider group to assume responsibility for all the care of a specified patient population." | 4.04.1 |
| "Expanding the scope of DRG payment for inpatient care is essentially a more limited version of episode-based payment and could become a building block for a full-fledged episode-based payment system. For example, consider a hip-replacement episode—bundling of post-acute care would incorporate payment for rehabilitation and associated services into the inpatient DRG payment. An episode-based payment for hip replacement would include these services as well as the surgeon’s and other physicians’ services provided to the patient for that episode. The broader episode payment would need to supersede the inpatient bundle. Initially, a full episode-based payment approach likely would apply to a more limited set of episodes than an expanded-DRG approach. Although it would be operationally challenging, both systems could run simultaneously with episode types included in the episode-based payment approach excluded from the expanded-DRG approach." | 4.04a.1 |
| "In early stages of an episode-based payment program, payers could focus on a small number of types of episodes that are relatively straightforward to define, measure and attribute and have the greatest potential for cost savings. Later program stages could expand to target more complex episodes and constellations of providers." | 4.05.1 |
|  | 4.05.2 |
| "Mandatory participation in an episode-based payment program suggests that payers will identify the responsible provider(s) and tell them either prospectively or retrospectively which patients and episodes they are responsible for, since it would be far less feasible to ask all providers to identify their own patients in a broad program. Mandatory participation also would provide payers with more comprehensive data on per-episode costs to support the development of bundled payments for later phases. The main disadvantage of mandatory participation is that it essentially limits payers to applying fee-for-service payments, because most providers currently do not work in organizations capable of accepting the financial risk associated with bundled payments. Moreover, the fragmented  and competitive nature of current provider markets poses substantial barriers to effective collaboration for many providers.24 In a voluntary program, payers could allow the smaller number of participating providers to identify their own patients prospectively, which may increase provider buy in but also increase the risks of “cream-skimming” and “lemon dropping” if providers try to assume responsibility for less-costly patients and avoid more costly ones. Payers could mitigate this risk by involving patients in confirming who their primary provider is. Payers could also face some fiscal risk, since providers that volunteer would probably already have lower-than average costs per episode than non-volunteers." | 4.05a.1 |
|  | 4.05c.1 |
|  | 4.05a.2 |
|  | 4.05a.3 |
| "Policy makers, however, unnecessarily limit the potential impact of payment reforms by making them invisible to patients and may even risk a backlash. One way to engage patients would be to provide incentives for them to favor more efficient and higher quality providers. This way, high-performing providers would not only get higher payments, but they would get additional patients as well. Loss of patients could be an even more powerful prod to low performers than payment penalties. Patients who are not constructively engaged in this manner might actually turn against reforms by defending the inefficiency of their favorite providers. Although Medicare’s first demonstration of bundled payments, which took place in the 1990s, had no beneficiary incentives, the ACE demonstration, which began in 2009, will share 50 percent of any savings with beneficiaries up to a maximum of the annual Medicare Part B premium, currently $1,157 for most beneficiaries. Consumer incentives can take the form of allowing patients to pay less when choosing a more efficient provider. For example, the most efficient providers for a type of episode might be identified as the reference group with whom consumers would have the lowest cost-sharing burden. Patients using the least efficient providers would pay the most. Private insurers have some experience in providing patient incentives through high-performance networks.26 Within each specialty, private insurers designate practices within their networks as high performing. Some employers provide explicit patient cost-sharing differences depending on whether a high-performing provider is used. The approach has been limited by each insurer using a somewhat different approach to identifying the most efficient practices and by their inability to pool data with each other and with Medicare. The approach might also work better if insurers varied payment rates according to a practice’s performance. Nevertheless, this existing model could be modified to engage consumers in broader provider payment reform." | 4.06.1 |
|  | 4.06.2 |
|  | 4.06.3 |
|  | 4.06.2 |
| **ARTICLE 5: Mechanic, 2016 ^5^** | |
| **Selected Text Excerpt** | **Reference No.** |
| "Both BPCI and CJR episodes of care are based on Medicare-severity-DRG (MS-DRG) “families.” BPCI includes 48 episode types, which include up to 15 MSDRGs each (see Appendix A). For example, renal failure episodes include three MS-DRGs (renal failure with major complication or comorbidity (MCC), renal failure with complication or comorbidity (CC) and renal failure without CC or MCC); CABG episodes have six. BPCI includes four distinct models: ■ Model 1 is based on episodes that encompass Parts A and B spending during a hospitalization and it essentially continues the 2009 CMS physician-hospital collaboration demonstration program. Participation has included only roughly a dozen New Jersey hospitals. Model 1 will not be continued past its original sunset date of March 31, 2016. ■ Model 2 combines spending for acute hospital care and post-acute care. Participants can select episodes lasting 30, 60 or 90 days after patients are discharged from the hospital. Model 2 has 631 participants in the risk-bearing phase. ■ Model 3 focuses on post-acute care episodes that begin with the start of post-acute services as long as care begins within 30 days of a hospital discharge. Model 3 participants can select 30, 60 or 90-day episodes. Model 3 has 871 participants in the risk-bearing phase. ■ Model 4 is a prospective bundled payment for Parts A and B during hospitalizations plus the cost of readmissions. CMS makes a prospective payment to participating hospitals that then must pay the physicians. Similar to Model 1, less than a dozen hospitals participate in Model 4." | 5.01.1 |
|  | 5.01.2 |
| "CMS will calculate CJR target prices as a blend of each hospital’s historical spending per episode and the regional average spending in the hospital’s census division. Initially, target prices will be based on two-thirds hospital-specific spending and one-third regional spending. In year 3 the prices will be one-third hospital specific and two-thirds regional. In years 4 and 5, target prices will be set at regional average episode spending levels. In both programs, CMS adjusts the prices for geographic wage index differences to create standardized payment amounts and removes indirect medical education, disproportionate share and other special payments, which will continue to be paid outside of the bundle. Episode prices are set for each MS-DRG bundle, and in CJR CMS sets separate target prices for hip fracture patients. Neither initiative uses additional risk adjustment." | 5.02.1 |
|  | 5.02.2 |
|  | 5.02.3 |
| "Both programs have stop-loss limits, which give hospitals additional financial protection, as well as stop-gain thresholds. BPCI participant gains or losses are capped at 20 percent of the aggregate target amount across each participant’s bundles. CJR hospitals are limited to gains of five percent of the aggregate target amount in the first two program years, 10 percent in the 3rd program year, and 20 percent in the last two years. CJR hospitals do not have downside risk in the first program year. In years 2 and 3, repayments for losses will be limited to 10 percent of the aggregate target amount respectively. In years 4 and 5 repayments will be capped at 20 percent. CMS proposes much lower limits on repayments from sole community hospitals, rural referral centers, rural hospitals and Medicare-dependent hospitals." | 5.03.1 |
| "CJR hospitals are not required to make any repayments to CMS in the program’s first year. In years 2 and 3, CMS will calculate hospitals’ repayment responsibility based on target prices with a 2 percent discount. Hospitals can also reduce this discount based on their quality score. A majority of hospitals will likely achieve sufficient quality for a 1 percent discount and repayment discounts will drop to 0.5 percent for top performers. The repayment discount rises to 3 percent in years 4 and 5 but it is reduced for hospitals that reach quality thresholds as shown below." | 5.13.1 |
|  | 5.13.2 |
| "CMS updates BPCI episode prices quarterly based on the actual national average change in Medicare spending for each of the 48 bundles. Therefore, neither CMS nor the hospitals know what the update factors will be until six months after the end of each quarter. This method of updating BPCI prices has been a source of substantial uncertainty for participants. In contrast, the CJR will update target prices using the annual Medicare payment system updates thus hospitals will know their target prices before the start of each performance period."..."The data, calculation methodologies and exclusions used by CMS to calculate its program’s performance have been opaque. This reduces the hospital’s confidence that gain or loss calculations are accurate and erodes confidence in BPCI generally. “We view BPCI as a tool to make our patients healthier” a senior CPH executive said. “It helps you look beyond day to day – one treatment at a time thinking to how to improve the health of your population and community. Our participation has been valuable, but unless CMS makes changes to the program, it will be difficult for small and rural hospitals to participate successfully.”" | 5.04.1 |
|  | 5.04.2 |
| "Under BPCI and CJR, CMS monitors post-episode spending for 30 days to identify any systematic increase in post-episode spending. The monitoring is intended to ensure bundling participants do not shift services out of the episode time period to reduce episode spending. If CMS determines that post-episode spending is systematically higher than it was during the baseline period, bundlers must return excess spending to Medicare." | 5.05.1 |
| "The vast majority of participants entered the program through third-party awardee-conveners that provide analytic and risk management services, and contract directly with CMS on behalf of the providers. Several conveners help manage the majority of episodes in BPCI with a single group, Remedy Partners, accounting for about half of the BPCI episodes (Exhibit 7)." | 5.06.1 |
| "One of the most challenging aspects of any bundled payment program is random variation in the average cost of treating a cohort of patients in a particular clinical episode. This occurs because of the substantial variation in the severity of patients (and number of cost outliers) over a given period of time. The problem is exacerbated because most providers have a relatively low case volume in any particular bundle. For example, if a hospital has 50 cases with average episode spending of $15,000 in year 1 and the exact same cases in the year 2 except that one patient became a $100,000 outlier case, the average per-episode cost would rise by 11.3 percent. If the hospital has the exact same distribution of patients over two years but with no outliers in year 1 and two $100,000 outliers in year 2, the average per-episode cost would increase by nearly 23 percent (Exhibit 10). If year 1 was used to establish a target price and year 2 was the performance period, the hospital would incur a large loss while if the outliers occurred in year 1 and not in year 2 the hospital would incur a large gain. Higher case volume substantially mitigates random variation. While a $100,000 outlier would add $1,700 to the average cost of the hypothetical hospital with 50 cases it would add only $170 per episode for a provider with 500 cases. In general, the average costs of surgical episodes vary less from period to period than the average episode costs of hospitalized patients with chronic medical conditions.  Bundling participants need strategies to address the risk of random variation. One approach is to reduce variation by selecting a portfolio of bundles to increase volume. Another is to purchase reinsurance or, under BPCI, contract with facilitator-conveners that are willing to share risk. (Facilitator-conveners cannot contract with Medicare in CJR but can contract with providers to supply reinsurance services.) BPCI conducts quarterly payment reconciliation, and, since quarterly volume is low, quarterly performance varies substantially. Rather than focus too much on quarterly results, participants should be aware of random variation, develop methods to reduce it and maintain a longer-term perspective that focuses on performance improvement."..."..."Also, as a smaller hospital, its volume and cost numbers vary substantially from quarter to quarter. The amounts reported as being owed to CMS or due to the hospital in a given quarter have changed significantly as CMS has updated the results. From a management and governance perspective in a small organization, knowing with certainty whether it has made $25,000 for a quarter or lost $50,000 is significant." | 5.07.1 |
|  | 5.07.2 |
|  | 5.07.3 |
| "Establish data analytic and information sharing capabilities. Bundlers have access to a vast array of new data that can help them evaluate the full spectrum of care that their patients receive and identify areas for performance improvement. Bundlers need to analyze the data quickly and transmit information to their physician and post-acute care partners. They need to develop systems that allow them to share patient data with care partners on a real-time basis. They also need to develop approaches to help determine the best post-acute care setting for individual patients upon discharge.  Track patients across the continuum of care. Hospitals and post-acute care providers historically did not routinely follow patients after they were discharged from the inpatient setting. With bundling, providers need personnel and systems that follow patients throughout the recovery process and respond rapidly if patients suffer complications or show signs of decompensation, such as failure of the heart to maintain adequate blood circulation." | 5.08.1 |
| "Redesign care. Participants in bundling need to develop standard care protocols and longitudinal care plans, establish new staff roles such as nurse navigators, develop systems where physicians or advance-practice clinicians round regularly in post-acute facilities, and establish processes to ensure smooth transitions between care settings that include “warm-handoffs” between providers." | 5.09.1 |
| "CPH faced several challenges in BPCI. One was identifying BPCI patients correctly. It has encountered differences of 20 percent or more in the number of CHF and total joint patients it identifies internally and the number identified in the CMS data." | 5.10.1 |
| "Dignity Health has hired new staff and reorganized its inpatient care coordination program in preparation for BPCI. The Dignity Health care coordinators ensure that patients make a smooth transition from the hospital to the home or to an appropriate post-acute provider. They work closely with naviHealth staff to coordinate postacute services for the BPCI patients. The hospitals have a database of community-based services, such as meals on wheels, transportation, home care, education and other supports as a resource for care coordinators and their patients." | 5.11.1 |

| **ARTICLE 6: Averill et al., 2009 ^6^** | |
| --- | --- |
| **Selected Text Excerpt** | **Reference No.** |
| "Episode payment would encompass the services delivered not only during the hospitalization itself but also during the prehospitalization and posthospitalization delivery of care (eg, outpatient, ancillary, physician office). One approach to defining episodes would be to isolate the prehospitalization and posthospitalization services that were associated with the reason for hospitalization (eg, all services related to the treatment of diabetes). Although the identification of services related to the reason for hospitalization during the prehospitalization and posthospitalization period for a relatively healthy individual can be done with a reasonable degree of accuracy (eg, a pregnancy episode encompassing delivery along with pre- and postpartum care, or a cholecystectomy in an otherwise healthy individual), such episodes of care constitute a small proportion of healthcare expenditures, especially for Medicare patients. Indeed, approximately 10% of Medicare beneficiaries consume 63% of Medicare expenditures (Kaiser Family Foundation, 2008)." | 6.01.1 |
| "Because the high-utilizing population is characterized by multiple comorbid conditions, it can be extremely difficult to accurately attribute the prehospitalization and posthospitalization services to the specific disease that was the reason for hospitalization. For example, for a patient who has congestive heart failure, diabetes, and renal failure and is hospitalized for uncontrolled diabetes, there is considerable uncertainty in identifying precisely which services are related to the diabetes care rather than the care of the heart failure or renal failure (eg, a posthospitalization emergency department visit for syncope could be related to the heart failure rather than diabetes). Further, since comorbid diseases interact and do not behave independently of each other, any attempt to isolate only those services that relate to the illness that was the reason for hospitalization will not be accurate for patients with multiple comorbid conditions (Hughes et al., 2004). As a result, the definition of an episode needs to be patient centered rather than disease centered. In other words, while a specific illness may have initiated (triggered) an episode, the focus of the payment for the episode needs to be on the total services provided to a patient and not limited to the services associated with the specific reason for the hospitalization. Because a patient-centered episode encompasses all services rendered to a patient during the episode, an episode payment system must include not only are cognition of the acute problem(s) that precipitated patient’s hospitalization but also the patient’s overall burden of chronic illnesses." | 6.02.1 |
| "There are 5 components of a patient centered episode: • Episode trigger: The hospitalization that precipitates the episode (eg, a hospitalization for coronary bypass surgery). • Episode acuity: The acuteness of the patient’s conditions at the time of the episode trigger hospitalization (ie, the severity of illness of the patient during the hospitalization). • Episode window: The number of days of prehospitalization and posthospitalization that are encompassed by the episode. • Episode service scope: The services included in the episode (eg, physician office visits, pharmaceutical usage). • Chronic disease burden: The extent of the patient’s comorbid chronic diseases at the beginning of the episode." | 6.03.1 |
| "A patient-centered episode would accumulate all services rendered within the episode scope that occurred before, during, and after the episode trigger hospitalization (ie, within the episode window). The expected utilization of services during an episode depends on the acuity of the patient at the time of the episode trigger hospitalization. For example, the use of services during a cardiac bypass surgery episode will be dramatically different for a critically ill patient admitted for emergency cardiac bypass surgery as opposed to a stable patient admitted for elective cardiac bypass surgery." | 6.04.1 |
| "Since most episodes will be between these 2 extremes, both the patient’s acuity at the time of the episode trigger hospitalization and the chronic disease burden of the patient at the beginning of the episode window must be simultaneously taken into account to understand and predict the expected utilization of services and costs incurred during the episode." | 6.05.1 |
| "In an episode system, CRGs predict episode resource use at the beginning of the episode on the basis of the patient’s prior diagnostic and service profile. Since CRGs predict subsequent resource use, they function in a manner similar to a risk adjustment system in a capitated payment system. COMPUTING EPISODE PAYMENT WEIGHTS. Essentially, every combination of an episode trigger, episode window, and episode service scope defines a unique type of episode. With a categorical episode unit of payment such as CRGs, this diversity is manageable because the process of establishing the projected episode payment amount is straightforward and simply involves computing the historical average resource use of patients in each CRG for each unique type of episode. The calculation of the payment weights for DRGs only requires 1 year of historical data." | 6.06.1 |
| "As a result, the CRG payment weights are essentially predictive, risk-adjusted payments expressed in terms of clinically meaningful prospective payment categories." | 6.07.1 |
| "These steps can be repeated for each unique episode cohort (ie, combination of episode trigger, episode window, and episode scope). As is done in IPPS, additional hospital-specific payment adjustments can be made for factors such as wage rates, teaching adjustments, and disproportionate share." | 6.08.1 |
| "However, if the episode encompassed an extended time period (6 months), it would essentially be a limited form of capitation with all the associated insurance risks for providers." | 6.10.1 |
| "The effectiveness of an episode-based approach to cost control will in large part be dependent on the participation and cooperation of physicians. Unlike managed care organizations, hospitals are currently prohibited by Medicare from establishing physician financial incentive programs such as gainsharing arrangements. If hospitals are to effectively managed under an episode-based payment system, such prohibitions must be removed." | 6.11.1 |
| "There is also a temporal issue related to the services delivered during the prehospitalization time included in the episode window. The hospital will be responsible for the payment of those services but will not be aware of that responsibility until the patient is actually admitted." | 6.12.1 |
| "Since the episode revolves around a hospitalization, the hospital will receive the episode payment and be responsible for paying for all services in the episode scope that are not directly delivered by the hospital. This in essence means that the hospital must act as a payer with all the claims, processing infrastructure necessary to receive and pay bills. In general, hospitals have infrastructure in place for billing services but have limited infrastructure for paying for services delivered by other providers. Thus, a significant expansion of hospital claims processing capabilities will be necessary for hospitals to manage under a full episode payment system." | 6.13.1 |
| "Further, hospitals have limited experience in negotiating prices for services delivered by physicians and other providers." | 6.14.1 |
| "As a result, full episode payment including pre- and posthospital care would need to be limited to scheduled admissions such as a total knee replacement." | 6.15.1 |
| "The chronic illness burden (the CRG) of the patient will need to be determined at the beginning of the episode, which will require that the diagnoses are reported from all sites of service before the beginning of the episode. This is not a problem for Medicare since it has the complete claims history of the patient. However, the hospital does not have access to that claims history." | 6.16.1 |
| The simplest form of a hospital-based episode would be to limit the episode scope of services to hospital care and include in the episode payment for the trigger hospitalization any subsequent hospital readmissions that occur within the episode window. | 6.18.1 |
| "A hospital-based episode of care prospective payment system can be phased in over time by gradually expanding the services included in the episode service scope and expanding the length of the episode window." | 6.17.1 |
|  | 6.17.2 |

| **ARTICLE 7: Bailit et al., 2013 ^7^** | |
| --- | --- |
| **Selected Text Excerpt** | **Reference No.** |
| "First, p+B172:C198ayment reform requires the adoption of new operating platforms that are not endogenous to health plans. That’s a big hurdle because most of the plan CFOs will look at the cost of adopting these new platforms as an incremental cost when they should, instead, look at it simply as a cost of doing business in a the new world of value based healthcare." | 7.01.1 |
| "Second, non-integrated providers can band together to accept financial risk and improve their collective performance. Remedy Partners has facilitated that process for hundreds of physicians and hospitals across the country. Their analytic support and push to clinical integration assures that the financial integration will work. This is an essential lesson to payers everywhere. They keep looking for integrated systems that can take full risk, when in fact efficiencies are far better optimized by integrating service lines and taking performance risk instead of insurance risk. Again, the CFOs of many provider organizations will get this wrong, for the same reasons the health plan CFOs will get it wrong." | 7.02.1 |
| "Two dyads originally reported as having operationalized payment, concluded their pilots and elected not to move forward with bundled payments. Both dyads reported success with their bundled payment pilots, though neither reported statistically significant financial gains. In both cases, the dyads defined success in terms of provider initiating efforts at innovative care redesign." | 7.03.1 |
|  | 7.03.2 |
| "Since the original study, the number of operating inpatient procedural conditions among the 19 studied dyads has increased 122 percent (from 9 to 20) and the number of operating outpatient procedural conditions has increased 200 percent (from 1 to 3). Chronic medical conditions are considered to have the potential for the greatest savings,2 and bundled payments for these medical conditions have increased 300 percent (from 1 to 4)." | 7.10.1 |
| "Both health plans report very strong and committed leadership support for their bundled payment initiatives. The plans’ leadership identified bundled payments as an essential strategy to promote broader-based and long-term delivery system transformation. Leaders at each of the plans view bundled payments as creating a competitive advantage through strengthening provider alliances and lowering costs. In other words, BCBSNC and Horizon consider bundled payments as a wave of the future, not a side endeavor. Similarly, both plans see bundled payments as a vehicle to achieving clinical integration among multiple providers. Horizon considers bundled payments as one of three integrated transformation strategies it is pursuing; the other two being the patient-centered medical home (PCMH) and the development of ACOs. Within that strategic framework, Horizon believes bundling payments enables specialists to improve and streamline care, activities essential for the successful development and execution of population based health programs such as PCMHs and ACOs. BCBSNC emphasizes the transformational outcomes of bundled payments as being strategically important. In addition to clinical integration, the plan’s desirable outcomes for bundling payments include: 1) providing a transparent methodology to compare performance across providers, 2) better collaboration among all participants—including the payer—within the bundled payment, and 3) shifting performance risk to providers while BCBSNC retains the insurance risk. BCBSNC and Horizon’s staff commitment to develop and implement the reimbursement strategy offer further evidence of plan leadership support for bundling payments."..."When asked to identify factors that have been key to success so far, the plans commonly identified three factors: 1. Commitment to the initiative by top leaders; 2. Adequate resources for program design and administration and provider contracting, and 3. An open mind to new ideas." | 7.04.1a |
|  | 7.04.1b |
| "Both BCBSNC and Horizon started bundled payments with knee and hip replacements because these are well-defined, high-volume procedures with opportunity for savings through care redesign."..."As a first step, both plans start a search for bundled payment partners by identifying high-volume providers. Horizon vets practices internally, examining their quality profile, contractual relationship with the plan, and level of practice sophistication." | 7.11.1 |
|  | 7.11.2 |
|  | 7.11.3 |
| "Horizon seeks partners who are looking to the future, understand that they must transform their practices to be successful, and have physician champions to lead the change. BCBSNC describes the high-volume practices it seeks as those that are willing to think outside the box, have leadership that understands the importance of practice transformation to reducing costs, and are early adopters of innovation. These practices also see themselves as “destination providers”—practices that patients view to be leaders in the field. Both plans evaluate practices by using the ECR Analytics software to identify potentially avoidable cost savings opportunities. While high levels of potentially avoidable costs indicate higher potential for savings, Horizon notes that practices with a low level of potentially avoidable costs can be good partners too because they believe there may be cost savings opportunity in the typical portion of the bundles as well." | 7.12.1 |
|  | 7.12.2 |
| " BCBSNC is flexible with its negotiations depending on which providers participate in the bundled payment. For example, if anesthesiologists are at the table, the plan includes their services in the bundled payment; if anesthesiologists are not at the table, the plan develops a financial model that excludes their services. As a result, BCBSNC emphasizes the need for flexibility regarding the included provider services, the financial model, the budget amount and the contracting structure. For example, depending on the local dynamics, BCBSNC either contracts with a single provider that subcontracts with downstream providers or the plan develops contracts with each participating provider separately.  BCBSNC describes the contracting discussions as part of the process of team building. All parties need to be open to others’ points of view during contractual discussions. These discussions provide an opportunity for providers to see situations differently, and an opportunity for the plan to see what the providers view as challenges. BCBSNC believes an open and flexible approach is essential to successfully negotiating a bundled payment contract. Depending on the motivation and time commitment of the partnering providers, the bundled payment contracting process can take as little as four months or as long as two years. Horizon’s approach to contracting is also very collaborative. The plan reviews historical data with prospective partners and develops budgets by using the ECR Analytics, and risk adjusts at the member level. Because of the successful execution of the hip and knee replacement program, Horizon is expanding its bundled payment program and preparing to scale the model. The plan expects that ‘onboarding’ a new practice will occur within 12 weeks of initial discussions." | 7.05.1 |
|  | 7.05.2 |
|  | 7.05.3 |
|  | 7.05.4 |
| Both plans emphasize the importance of transparent data sharing to obtain provider buy-in, build trust, and motivate transformation. Data sharing is essential during the contracting process to demonstrate to the providers the logic and fairness of the bundle definition, the appropriateness of the budget and the opportunities for cost savings. Plan representatives report that physicians are often surprised at the total cost of care and find the data to be eye-opening. Both plans hold monthly meetings with providers to keep them abreast of claims data for contracted bundles. The data sharing has helped providers begin to build a better understanding of care provided by downstream providers and the cost of those services. Plans indicated that these monthly sessions offer participants the opportunity to discuss new practice improvement ideas and ways to better coordinate with other providers. BCBSNC’s monthly meetings focus on analyzing where care was rendered, and looking for leakage (services provided by providers not part of the bundle). The plan shares data to providers at an aggregate level and not at the CPT code level." | 7.06.1 |
| "Use of quality data. BCBSNC and Horizon each built quality data into their bundled payment program. Horizon used a provider advisory council composed of participating physicians to identify and define performance measures that the plan collects and reports back to the practices. The data  collected is a “toll gate” meaning that it must be reported before any savings that results from the bundle is distributed to the providers. The data includes functional analyses relating to hip and knee replacements, such as range-of-motion capabilities in a specified number of days after surgery. The plan is also analyzing the incidence of “Never Events” and readmissions, and monitoring the occurrence of pulmonary embolisms. If Never Events or readmissions occur, the plan and providers conduct a major review to determine what could be done in the future to avoid such an event. Horizon reports zero Never Events and very few readmissions in 1,000 surgeries. Recently, Horizon added patient experience measures into its quality assessment, allowing the plan  to monitor how well the patients perceive their care. Plan representatives report that patient  satisfaction levels for patients whose case is subject to a bundle are higher than for those who are not. | 7.07.1 |
|  | 7.07.2 |
| "The manual reconciliation process is quite time consuming. BCBSNC originally chose to administer its bundles manually because it needed to demonstrate that a bundled payment was a win/win strategy for itself and providers before moving to automate the process. Now that both plans are ready to make bundled payments a viable permanent reimbursement strategy, both have decided to engage a claims adjudicator to automate the reconciliation process. The claims adjudicator, TriZetto7(in both cases), is able to re-price claims and assign them to the bundle using definitions set forth by the plans. In addition, TriZetto can implement bundled payments that are either prospectively paid or retrospectively reconciled. BCBSNC is currently working with TriZetto and HCI3 to ensure that TriZetto has the appropriate PROMETHEUS Payment algorithms and the health plan’s provider contracts. The plans described the process of readying TriZetto as time consuming and involving months of work."..."BCBSNC sees the automation of the reconciliation process and offering both prospective and retrospective bundled payment methodologies as critical to success." | 7.08.1 |
|  | 7.08.2 |
| "BCBSNC sees the automation of the reconciliation process and offering both prospective and retrospective bundled payment methodologies as critical to success." | 7.14.1 |
| "Finally, both plans view patient engagement as key. Patient agreement to use the participating providers assures more coordinated and efficient care, two goals driving both of these plans adoption of bundled payments. Horizon collects patient satisfaction information and is reporting high satisfaction rates. Both plans are still in the early stages of addressing the challenge of patient engagement." | 7.15.1 |
| "First, Medicare typically spends as much or more in the 90 days post-hospitalization than it does for the hospitalization itself. The episode-initiating organizations are hospitals—which control half or less than half of the cost of a total bundle. Under BPCI, hospitals are at substantial risk for the care  provided-post discharge. Without an integrated network of post-acute care providers or sophisticated coordination among providers, hospitals could face losses under the program’s financial arrangements." | 7.16.1 |
| "Third, Medicare built 48 different episodes of care, and Brandeis found that each hospital may not always have a statistically significant number of patients within each bundle, which can lead to wide variation in patient-severity, and therefore cost. This lack of statistically valid groupings can lead to significant financial risk for providers year-over-year. Providers can mitigate this risk by limiting the number of bundles it chooses to join. However, since CMS released the final Bundle Payment Pricing data set just prior to the publication of this study, many providers have been unable to make those decisions. In addition, CMS’ reliance on DRGs as the determinant for classifying the bundle, places providers at significant risk simply due to the heterogeneity of diagnoses included in a DRG, especially the procedural ones." | 7.09.1 |

| **ARTICLE 8: Chee et al., 2016 ^8^** | |
| --- | --- |
| **Selected Text Excerpt** | **Reference No.** |
| Excluded. This review specifically focusses on pay-for-performance models, rather than bundled payment models. | n/a |

| **ARTICLE 9: Conrad et al., 2014 ^9^** | |
| --- | --- |
| **Selected Text Excerpt** | **Reference No.** |
| "NH’s small size and strong civic culture encourage collaboration, and there is growing consensus among stakeholders on the need to control health care spending. The availability of an all-payer claims database and a partnership with the University of New Hampshire’s Center for Health Analytics and other entities have enabled stakeholders to perform claims-based analyses and track performance metrics across organizations and over time. The CHI’s leadership and expertise in facilitation have reinforced relationships and goodwill. The physicians’ input and buy-in through their participation in the project’s clinical subcommittee have laid the groundwork for NH’s payment reform, as has experience with this state’s medical home pilot, which the CHI coordinated as well." | 9.01.1 |
| Many payment reform efforts require significant changes in the care delivery and support systems, for instance, in the data- and claims- processing infrastructure, electronic health records (EHRs), and staff roles. But providers’ limited ability to meet infrastructure demands for payment reform has made them resistant and hindered its implementation. Some providers thus favor reforming the delivery system through collaborative processes and changes in payment to support the proposed system redesign.27 Payment reform efforts likewise face challenges in setting the “right” level of payment, namely, payment that rewards the appropriate delivery of care and discourages inappropriate care. Particularly when facing the costs associated with system redesign, providers might perceive the expected benefits of new payments as insufficient." | 9.01.2 |
|  | 9.01.3 |
| "The lack of significant competition in the NH health insurance market has dampened private  insurers’ incentive for payment innovation. Similarly, the hospitals’ and health systems’ firm  market control has discouraged independent medical groups and hospitals from experimenting with different models of payment innovation." | 9.02.1 |
|  | 9.02.2 |
| "The existence of a statewide APCD and a robust capacity for managing, encrypting, and opening access to data are important enablers. Providers have invested much in the ACO model."…"The previous vendor’s inability to provide sufficient reporting and analytic support has limited the APCD’s practical utility. The lack of electronic health records (EHRs) in many small practices has inhibited the efficient input of data into the database, and some provider organizations have been reluctant to share their data with employers." | 9.03.1 |
| "Collaboration among stakeholders becomes more and more difficult as the possible financial and organizational consequences of payment reform increase. In contrast to quality improvement, in which “win-win” alliances form more easily, changing payment levels and methodologies inevitably reduces income and adds financial risk for some parties (eg, certain provider systems and specialists) even as it raises income and reduces risk for the “counterparties” to those contractual arrangements (eg, plan sponsors, consumers, and PCPs)." | 9.04.1 |
| "The sharing of clinical and claims data among employers, health plans, and providers raises considerations of privacy, confidentiality, control over the data, and antitrust law. In particular, concerns about health plans’ willingness to share data and provider organizations’ development of their own potentially redundant data systems must be addressed." | 9.05.1 |
| "About 70% of the region’s medical practices are small (5 or fewer physicians), which need EHR capability and interoperability that will allow them to track patients across care settings. Furthermore, even though overcoming legal and privacy issues in data sharing will be a challenge, the practices are putting together a third-party nondisclosure agreement to address these considerations." | 9.06.1 |
| "...and (3) virtual provider teams (payments for results go to all providers and clinics caring for the patient’s condition). The POP program initially targets 3 chronic conditions with the highest  costs: congestive heart failure (CHF), chronic obstructive pulmonary disease (COPD), and diabetes. For each condition, the program defines eligible provider specialties, identifies a patient’s providers through claims or referrals, computes the program’s payment, and distributes it to the virtual team. The program’s payments are projected to be either budget neutral or potentially cost saving. On January 1, 2013, the POP began tracking claims for these 3 conditions in each coordinated care organization (CCO), with the payment of incentives starting after July 1, 2013. The CCO is a network of all health care providers agreeing to collaborate in their local areas for persons with health care coverage through the Oregon Health Plan (Medicaid)." | 9.10.1 |
| "External government forces are a key facilitator. The Oregon legislature and federal government require CCOs to have alternative payment methods based on quality and reduced cost growth, which has created a receptive environment in the Salem area for implementing the POP. Another external facilitator is funding from the RWJF, which has raised local awareness and credibility of the project. Local facilitators include a history of collaboration and innovation, as well as the leadership of the PCF, PH Tech, and WVP Health Authority. PH Tech has invested substantial resources to develop the POP computer software and routinely collects much of the medical claims data for operating the POP." | 9.11.1 |
|  | 9.11.2 |
| "After an early meeting, the senior executives delegated the decision making to other, lower-level, authorities in their organizations. Along with setbacks to the project’s momentum due to a lengthy implementation process, the organizational executives’ commitment dissipated when challenges arose. In addition, the providers’ and payers’ priorities fundamentally diverged. The providers saw the reform as improving shared decision making by providers and patients, whereas the payers stressed the potential for cost savings and changes in incentives." | 9.12.1 |
|  | 9.12.2 |
| "Oregon has a history of collaboration, innovation, and open communication, and its health system reforms create a receptive environment for local innovation. Trust also is instrumental: Quality Corp is regarded by the health care community as a credible data analyzer and provider of information about health system performance. Finally, the initiative is being implemented through each plan’s existing contracts with each medical group. Physician leadership in the medical groups is necessary for the successful implementation of collaborative care through nurse care managers."..."One challenge to implementation is data timeliness and accuracy. Utilization data are reported quarterly to medical groups, but reporting emergency department (ED) visits in real time would aid in managing care transitions." | 9.13.1 |
| "Although most high-risk patients want care management, not all patients want to participate. In addition, despite being defined as being high risk by their health plans and medical groups, some patients believe they are healthy." | 9.14.1 |
| "After the pilot started, changes in the HCA project leadership created some discontinuity in convening stakeholders and maintaining collaboration. Market competition also is a factor: Health plans in Washington State have a history of competing rather than collabo- rating in the marketplace. Another challenge is funding: Although the RWJF provided funds to support project management in the pilot’s early stages, the funds were insufficient to support the staffing necessary for this large, complex undertaking." | 9.15.1 |
| "In addition to these themes, driven by the nature of the particular payment innovation being  employed, other common themes emerged in this study. For example, the presence of a neutral  convening organization that functions as an honest broker of collaboration among parties with  different interests is a powerful facilitator of payment innovation. The role of the convener is fundamental to finding common ground among competitors, managing stakeholders’ expectations, and reconciling competing priorities. The continued infusion of outside investment—financial and human capital, in particular—catalyzes and reinforces payment innovation, and the convening organization often plays a leading role in securing external resources (eg, grants), which act to maintain early and midterm momentum." | 9.16.1 |
|  | 9.16.2 |
| "Another overarching theme is the ubiquity of information- and data- related barriers. The  prevalence of disparate EHR systems that are not interoperable or cannot adapt to one another, FFS-based billing and transaction systems incompatible with new modes of payment (eg, reference pricing), and difficulties with patient attribution all impede the advance of value-based payment. The absence of “real time” data—for example, the instantaneous notification of primary care offices when their patients present in EDs—compromises delivery systems’ efforts to manage utilization and costs." | 9.17.1 |
|  | 9.17.2 |
| "True value-based payment innovation, which transcends shared savings and modest care coordination payments, requires a “burning bridge” that precludes staying with the status quo of FFS. To the extent that multistakeholder coalitions tend toward sustaining the status quo rather than engaging in disruptive innovation,42 these efforts are most likely a transitional stage toward independent provider-payer combinations of integrated care delivery and value-based payment. Managing such competition will demand more transparent public pricing, a common public performance measurement of price and quality, and antitrust vigilance." | 9.18.1 |
| "Similarly, on the demand side, health plan cost sharing could be designed to encourage the selection of higher-value provider organizations. The ACOs being adopted in New Hampshire, Maine, and south- western Pennsylvania illustrate this prospective payment model, with variants of shared savings, bundled payments, and capitated, global payment arrangements involving a mix of public and private payers (CMS, state employers, and private insurers)." | 9.19.1 |
| "Large, vertically linked organizations could increase the participating providers’ pricing power relative to that of private insurers and self-insured employers, and—when the providers’ market shares substantially increase—these arrangements should be subject to scrutiny by the Federal Trade Commission and state attorneys general to ensure that better clinical quality and reduced costs, rather than higher prices, are the market outcome." | 9.20.1 |
| "Innovations such as value-based benefit design and reference pricing will encourage purchasers and consumers to demand value by increasing their economic stake in health care decisions." | 9.21.1 |

| **ARTICLE 10: Conrad, 2015 ^10^** | |
| --- | --- |
| **Selected Text Excerpt** | **Reference No.** |
| "I assume that the desired provider behavior is “value” :maximum health beneﬁt for the least total opportunity cost (including provider production costs, administrative costs, and patient opportunity costs)." | 10.01.1 |
| "The time lag between ultimate health outcomes and multiple inﬂuences (e.g., care from different providers, patient response, and comorbidities) beyond provider control implies that compensating providers on outcomes is potentially unreliable and unfair. This inherent measurement problem works against purely outcome-based compensation."..."As long as measurement of process quality and outcomes is unbiased in its design, and conducted with sufﬁciently large samples to produce relatively precise estimates of performance, prospects for value-based payment seem positive. This suggests that a mixed payment model—based on measures of process quality, health outcomes, and transaction price measures— has the potential to improve the tradeoff between the higher relative measurement costs of paying on the basis of value and the potential patient health beneﬁts of doing so." | 10.01.2 |
|  | 10.01.3 |
| "Optimal payment models will also be context dependent: baseline market conditions and structural relationships between purchasers, plans, provider organizations, and patients will vary geographically and over time, thus requiring a different mix of incentive size and structure." | 10.02.1 |
| "Behavioral economics suggests that a penalty of given size for failing to advance health beneﬁt will more strongly encourage providers to deliver improved health than an equally large reward for advancing health beneﬁt." | 10.03.1 |
| "A prospectively ﬁxed payment can be expected to elicit stronger behavioral response than a retrospective payment based on parameters not speciﬁed in advance. To illustrate this, it is sufﬁcient to acknowledge that individual providers are predominantly risk-averse. Hence, to induce them to participate in risk-based contracts, the level of expected net income must exceed income under FFS payment, to compensate the individual provider for bearing actuarial risk." | 10.04.1 |
| "A prospectively ﬁxed payment can be expected to elicit stronger behavioral response than a retrospective payment based on parameters not speciﬁed in advance. To illustrate this, it is sufﬁcient to acknowledgethatindividualprovidersarepredominantlyrisk-averse.Hence, to induce them to participate in risk-based contracts, the level of expected net income must exceed income under FFS payment, to compensate the individual provider for bearing actuarial risk.." | 10.04.2 |
| "Peers’ performance is not controllable, whereas the provider’s own behavior is directly under his or her inﬂuence. This lack of control weakens incentive effects." | 10.05.1 |
| "The main emphasis in this paper is on extrinsic ﬁnancial incentives, but nonﬁnancial incentives must be considered in parallel, for example, reputation or brand, intrinsic motivation, and altruism. Will the use of ﬁnancial incentives as extrinsic motivators tend to “crowd-out” intrinsic motivation? Put another way, will use of strong ﬁnancial incentives tend to reduce providers’ commitment to professional norms and their inherent motivation to act ﬁrst and foremost in patients’ interest" | 10.06.1 |
| "Optimal provider payment incentives are shaped by nuances of ownership (i.e., for-proﬁt vs. not-for-proﬁt for hospitals) and provider organizational form (e.g., integrated medical group vs. independent practice association [IPA]). Ownership is an important ﬁnancial incentive, in that owners capture their share of organization net income and are motivated to pursue maximum proﬁt through cost minimization and strategic pricing. The implication for payers is that not-for-proﬁt providers will have weaker incentives than for proﬁts to hold out for above-competitive prices, which will affect the level of contracted prices, but not necessarily the method of provider payment. A study by Lynk (1995)found that while mergers between private for-proﬁt hospitals led to increased prices, those between not-for-proﬁts were followed by reduced prices. Dranove (1988) outlines the theory and certain empirical support behind the argument that, other that, other things equal, not-for-proﬁts will price at lower levels than for-proﬁts." | 10.07.1 |
| "Organizational form inﬂuences incentive design. Gutermanet al.(2009) present the case for aligning payment form with organizational form: taking into account the capacity of the organization to accept actuarial risk (variation in health beyond the control of the provider organization and individual provider)and inducing the individual provider and organization to minimize performance risk (variation in health due to provider errors: undertreatment, overtreatment, or mistreatment). Table 1 illustrates my extension of this reasoning. The basic idea is that small independent medical practices are poorly equipped to bear signiﬁcant actuarial risk for random variation in health status. Consequently, FFS arrangements will be most feasible for them, potentially tied to adjunct pay-for-performance (P4P) incentives based on measures of clinical quality and patient experience, but not to health outcomes or total cost per patient over time—reasoning that the smaller sample size would imply greater random variation in average cost and health outcomes." | 10.07.2 |
| "Either government or an otherwise neutral organization is required to design and enforce these price and quality transparency mechanisms. Only government or a quasi-governmental public–private partnership underpinned by organizational public commitments can monitor and regulate provision of “public goods,” such as common investments in public health information infrastructure; agreements on quality, outcomes, and patient experience measurement; and value-based payment methods (not levels of payment)." | 10.08.1 |
| "Over time, less efﬁcient providers will cease production of services paid below their marginal costs. The administrative and informational costs of designing FFS payments with this efﬁciency property are one practical argument against basing a value-based system on FFS." | 10.09.1 |
| "• Public policy should invest in serious cost accounting for both hospitals and medical groups to better measure the production costs of different services. Such activity-based, fully allocated cost systems exist in the private sector for hospitals, and a revised, updated resource-based relative value (RBRVS) schedule could be applied as a baseline for costing units of professional services, for example, for physicians and medical groups. • Policy makers should deploy comparative effectiveness research to determine the marginal health beneﬁts of different services. That comparative effectiveness research is directly applicable to VBID and should be deployed beyond the current emphasis on pharmaceuticals. Setting unit prices close to the unit cost of services solves the problem of “demand inducement.”" | 10.09.2 |
| " Shared savings incentives also are weakened by the delay in retrospective determination of cost savings." | 10.10.1 |
| "The answer to the “crowd-out” question depends on whether patient health beneﬁt and provider net income substitute for or complement one another in the provider’s objective function. Payment level and method are major determinants of net income, so payment according to value would tend to drive patient health beneﬁt and provider net income in the same direction. Thus, rather than inducing crowd-out, value-based incentives would tend to make patient health beneﬁt and provider net income complementary objectives." | 10.06.2 |
| "Finally, targeted public and private investment in technologies and systems required to integrate information and achieve interoperability among disparate health information systems is an absolute requisite for enabling value-based payment incentives to be translated into action along the continuum of patient care. Current stages of meaningful use in electronic health record incentives and certiﬁcation provide a useful policy implementation framework for information support needed to drive value-based payment: data capture and sharing, advancing clinical processes, and improving outcomes. Real-time, clinically and managerially actionable data at these three levels of utility would create a technical platform for value-based payment and care. Without fundamental change in health information technology that uniﬁes clinical and economic data for use at the point of care, value-based payment will be limited to marginal adjustments to a FFS, encounter-driven healthcare system." | 10.11.1 |

| **ARTICLE 11: Conrad et al., 2016 ^11^** | |
| --- | --- |
| **Selected Text Excerpt** | **Reference No.** |
| "System readiness is a preadoption stage when payers, health plans, health care organizations, and providers become aware of the payment reform model(s) and learn how the model works and about its potential consequences. Based on this knowledge, health organizations may abandon payment reform or move toward a state of readiness for reform with buy-in from most stakeholders, widespread perceptions that FFS payment is not working, protected time and resources for reform, and capacity to evaluate its impacts. Greater readiness increases the likelihood of the health organization to adopt, implement, and sustain payment reform." | 11.01.1 |
| "Internal factors, such as the type and structure of the organization, also play a role. For instance, hospitals have less interest in primary care payment reform and instead move toward improving surgical and other hospital services through other VBP models. In the case of multi-stake holder coalitions engaged in payment reform, each member of the coalition has its own internal considerations. For example, a health plan within a coalition may have executive leadership supporting payment reform; a medical group member may have financial, clinical, and other factors that affect the member’s role and participation in the coalition, such as the different types of payment in the medical group and the group’s market share. Each member’s relationships with other members of the coalition influence his or her choices of reform objectives and payment models. Organizations consider context as they develop one or more objectives that define the specific expected outcomes of VBP reform, such as reducing costs and improving quality of care, patient experience, and health. The existence of multiple and potentially competing interests among the multiple stakeholders inevitably will shape the definition of objectives, strength of collaboration, and the pace and fidelity of implementation. The objective(s), in turn, directly affect the conceptual design of the VBP reform strategy. Payment design decisions include choices regarding the unit of payment (e.g., PMPM or per episode of care), the breadth and nature of services and care settings and providers subject to the VBP, the supporting health plan benefit design and patient incentives, and alignment of the payment incentives with delivery system design." | 11.02.1 |
| "Implementation includes the redesign of new clinical care processes and information systems, measurement and reporting protocols, and other components of delivery and payment reform. Applying the concept of a “tipping point” (Gladwell, 2002), organizations and providers may change their care processes only if payment reform changes financial incentives for a sufficient percentage of their patients." | 11.01.1 |
| " In general, the fewer and less imposing the barriers, the more likely the payment and delivery reforms will be assimilated by the organization(s). Implementation is inherently nonlinear, with setbacks and unexpected events, and in the end the reforms may or may not be successfully routinized (Greenhalgh et al., 2004; Rogers, 2003). Moreover, routinizing payment reform is no guarantee that the changes will be sustained over the long run" | 11.03.1 |
| "There have been impediments to VBP. For example, New Hampshire providers have expressed some reluctance to adopt capitation or provider risk-bearing payment models due to mixed experience with capitation, especially in the 1990s. Employers’ preference for payment based on actual claims, which document services received by members for the premiums paid, coupled with providers’ familiarity with FFS payment, also have stymied VBP—especially among self-insured purchasers. Creating VBP arrangements between physicians and hospitals in areas where provider organizations typically are not part of integrated delivery systems is exceedingly difficult. Due to population and provider distribution in rural areas, it can be difficult to craft contractual arrangements between PCPs, specialists, and hospitals." | 11.04.1 |
| " A local multi-stakeholder committee, Salem Area Community Health Information Exchange, is slowly developing a common information system. The birth of Salem’s CCO may accelerate development of a community-wide data warehouse and electronic medical record. The project’s objective was to improve the quality and efficiency of health care in Marion and Polk counties by paying a virtual provider team when a minimum percentage of patients achieve all clinical targets for a specific condition. Physicians Choice Foundation is implementing the project mainly in collaboration with the local independent physicians association, WVP Health Authority and its subsidiary, MarionPolk Community (Medicaid) Health Plan, and Performance Health Technology, which is leading design and development of payment strategy and provides claims administration for 60,000 persons receiving care through MVP. POP has developed a payment reform approach that blends the concepts of P4P with value-based health insurance, or paying extra for health services that generate better clinical results based on scientific evidence, ultimately to improve quality and efficiency of care." | 11.05.1 |
|  | 11.05.2 |
| "... and POP’s complexity and consequent difficulty in explaining the program to medical practices." | 11.04.1 |
| "The principal barriers to implementation are the challenging logistics of assembling many players across California at the same table, the difficulty in implementing a unified hospital and physician bundled payment due to differing perspectives and the state prohibition of employment of physicians by hospitals, patient expectations of “the perfect baby” militating in favor of C-section delivery, competing time and energy demands on providers from rapid movement toward increased adoption of electronic health record systems, and the difficulty of scaling maternity care practice improvement, given competing demands on CMQCC leadership and the supporting clinical experts." | 11.05.2 |
| "Tremendous statewide effort is required to overcome the sheer inertia of large systems, and fatigue is arising among stakeholders from the pace and scope of reform. Much uncertainty exists about federal waivers, and what the final payment model will look like. Furthermore, health care organizations and providers have little experience bearing financial risk and viewing a service as an expense (in population-based payment) rather than revenue (in FFS), which slows planning for payment reform. Fear of change and instances of lack of trust have inhibited collaboration among stakeholders. Finally, the achievement of Vermont’s payment objectives will be influenced by whether CMS awards a federal waiver from federally set Medicare and Medicaid payment regulations." | 11.06.1 |
| "Clear and consistent communication about movement toward larger reforms is vital…"…"Replacing volume-based FFS carries great uncertainty for payers, health care organizations and other groups, particularly for those that would bear most of the financial risk under the new payment models." | 11.07.1 |
| "However, effects of VBP reforms on outcomes may be smaller when FFS remains dominant." | 11.06.2 |

| **ARTICLE 12: George et al., 2014 ^12^** | |
| --- | --- |
| **Selected Text Excerpt** | **Reference No.** |
| "The economic principle behind a bundle is to separate technical risk (things clinicians can manage) from probability risks (thing outside their control), and transfer the former to clinicians and the latter to insurers. Perhaps one of the most important factors driving Duke's decision is the inherent variability of CHF; they believe separating technical from probability risks in CHF would be very difﬁcult. Duke expressed concern that efforts aimed at decreasing admissions for CHF might, in a bundled payment, create ﬁnancial incentives that were misaligned with the goals of improving patient care. Namely, a shift in volumes to higher-acuity CHF inpatients might over-emphasize probability risks compared to prior years." | 12.01.1 |
|  | 12.01.2 |
| "The team at Colorado prepared an environmental analysis of payment reform, and for a number of years, was engaged in a number of Colorado state initiatives for state-wide bundling in Medicaid and the private sector. Colorado viewed the model as an expansion of a previous demonstration model – this familiarity with the payment model and evidence of cost savings helped minimize the associated risks." | 12.02.1 |
| "A key component of sustainability is to provide meaningful recognition to such clinician leaders in academic and other settings. In addition, clinicians could perhaps satisfy licensing or board certiﬁcation requirements with direct, meaningful involvement in delivery reform. Such innovative uses of recertiﬁcation might create broader interest in clinical redesign and payment reform." | 12.03.1 |

| **ARTICLE 13: HFMA / Humana, 2015 ^13^** | |
| --- | --- |
| **Selected Text Excerpt** | **Reference No.** |
| "Key among these capabilities are the following: ■ Eligibility verification: The ability to effectively identify patients covered under a value-based payment arrangement ■ Interoperability: The ability to aggregate clinical information across networks and between hospitals and physician practices ■ Business intelligence: The ability to collect, analyze, and model data ■ Real-time data access: The ability to provide mean­ingful data to care providers at the point of service ■ Care standardization: The ability to provide infra­ structure that supports use of data to standardize care processes ■ Assessment of return: The ability to monitor value­ based contracting revenue opportunities versus costs of implementation ■ Chronic care management: The ability to provide systems and processes that support wellness and management of patients with high-volume, high-cost chronic diseases ■ Post-discharge follow-up: The ability to support patients post discharge with systematized follow-up (e.g., home health services, structured patient follow-up protocols) ■ Physician compensation: The ability to compensate physicians in a way that flexes to accommodate both fee-for-service and value-based models." | 13.01.1 |
| "Views on interoperability are of particular significance, as that is not only where organizations currently rank weakest but also where nearly 70 percent of financial executives anticipate their organizations will need to be extremely capable in the near future." | 13.01.2 |
| "To some extent, making the business case for further IT investment is eased with widespread recognition of resulting opportunities. Competencies around data use ranked highest among respondents when it came to factors that would best enable success under value-based payment. In particular, analytical support (business intelligence and actuarial), use of consistent care quality measures, and ability to monitor adherence to medically recommended regimens at the patient level are seen as most likely to support organizations' abilities to take on risk." | 13.02.1 |
| "Contain Initial exposure to financial risk. Providers should be cautious about the degree of risk they are willing to assume, especially if they are relatively new to value­ based payment models. "Starting out with 'upside only' payment models or models that cap potential losses at a certain amount can help limit risk as an organization develops its capabilities," Landman says." | 13.02.2 |

| **ARTICLE 14: Hussey et al., 2012 ^14^** | |
| --- | --- |
| **Selected Text Excerpt** | **Reference No.** |
| "Several types of undesired effects of bundled payment have also been postulated. The most significant potential undesired effects include underuse of effective services within the bundle, avoidance of high-risk patients, and an increase in the number of bundles reimbursed (increasing health spending). Providers under bundled payment may “game” the system by changing coding practices to maximize reimbursement for the bundle (“upcoding”) or by moving services in time or location to qualify for separate reimbursement (“unbundling”)." | 14.01.1 |
| "The effects of most key contextual factors were not addressed by any reviewed studies. Several studies compared differential effects of bundled payment on spending among for-profit and not-for-profit providers. In general, for-profit providers experienced larger declines in utilization under bundled payment than their not-for-profit counterparts (including U.S. and nonU.S. hospitals, skilled nursing facilities, inpatient rehabilitation facilities, and home health agencies)." | 14.01.2 |
| "Instead of eliminating low-value services, bundled payment could lead to underuse of appropriate services, with potential adverse effects on patient outcomes.6 In the absence of robust risk adjustment of bundled payments, providers may select low-risk patients and avoid those with higher risks (and costs)." | 14.02.1 |
| "In the absence of robust risk adjustment of bundled payments, providers may select low-risk patients and avoid those with higher risks (and costs).6 Providers may “game” the system by changing coding practices to maximize reimbursement for the bundle (“upcoding”) or by moving services in time or location to qualify for separate reimbursement (“unbundling”)." | 14.03.1 |
| "Concerns have also been raised about the administrative feasibility of bundled payment programs, particularly in establishing accountability and a mechanism for distributing payment among otherwise independent providers who participate in providing services that are part of a bundle." | 14.04.1 |
| "The study notes hospitals serving relatively few or many patients with low socioeconomic status (SES) responded to the IPPS differently in the year of the reform and afterwards. Specifically, in the year of the reform average length of stay decreased in high SES hospitals and increased in low-SES hospitals. In later years, average length of stay decreased in low-SES hospitals and did not change in high-SES hospitals." | 14.05.1 |
| "Finally, payment systems differed in implementation strategy, with some systems undergoing a lengthy phase-in period and others immediately supplanting the previous payment system."…"While gradual phase-in introduces challenges for evaluators, they may be warranted given providers’ reported difficulty adjusting administrative staffing, finances, and provider relationships to new reimbursement regimes." | 14.06.1 |
|  | 14.06.2 |
|  | 14.06.3 |
| "Policymakers and evaluation researchers must recognize the tension between producing timely, practical evidence and conducting rigorous evaluations. The most rigorous study designs are usually only feasible when policymakers plan for an evaluation experiment in the course of implementation." | 14.07.1 |

| **ARTICLE 15: Matchar et al., 2015 ^15^** | |
| --- | --- |
| **Selected Text Excerpt** | **Reference No.** |
| "One study predicted that bundled payments could lead to annual savings of $15 billion if it was applied to 245 episode types in Medicare and paid at the median of the current Medicare existing service fees.15 Another study estimated that under optimistic scenarios, bundled payment for 6 chronic conditions and 4 acute conditions or procedures requiring hospitalization could reduce national healthcare spending by 5.4% between 2010 and 2019.18 Interestingly, the authors also forecasted that bundling payments applied to hospital-based services only would reduce spending by just 0.1%. This is because Medicare already bundles hospital payments through the DRG mechanism, resulting in limited savings opportunities." | 15.01.1 |
| "The second unsuccessful bundled payment effort was initiated by the Integrated Health Association (IHA) in California.28 The IHA collaborated with the RAND Corporation to implement bundled payment for orthopedic surgery targeting commercially insured Californians <65 years old. The role of IHA was to manage the planning and implementation process, including forming a technical committee consisting of specialist physicians and representatives of health plans and hospitals to define the bundled payment. The bundle for knee replacement and total hip replacement included facility, professional, and medical implement device charges for inpatient stay plus a 90 day postsurgical warranty to cover services related to complications and readmissions; other postacute care was excluded. Despite optimism and intensive efforts by the IHA, the study suffered several setbacks and experienced significant delay in implementation. In particular, parties could not agree on a bundle definition. Further, several plans and hospitals dropped out of the project, and ultimately, only 2 hospitals eventually signed contract with the health plans. The resulting low volume of orthopedic procedures subject to bundling (only 35 cases over 3 years) precluded an evaluation of the impact of the demonstration project on cost and quality for orthopedic procedures." | 15.01.2 |
| "Several factors were posited as contributing to the positive impact of the Texas Institute’s effort. First, the bundle applied to a large volume of homogeneous patients in need of organspecific standardized procedures. The demonstration also benefited from the presence of an extensive patient database, which allowed an accurate assessment of profiling the population to be covered in the bundle and tracking actual costs. Other factors include physician-lead efforts in cooperating with providers and payers and the involvement of physician specialists with an established history of working together to provide patient care." | 15.02.1 |
|  | 15.02.2 |
| "Several care delivery changes were reported for the Geisinger experiment. Surgeons reviewed published guidelines and translated them into verifiable, actionable care processes with unequivocal definitions; 40 elements of care were identified and became the foundation for care process changes that were practical, measurable, and accountable to specific individuals. The experiment also led to better outcomes in hospital care utilization." | 15.03.1 |
| "Another salient feature of stroke is that the opportunity for improving patient outcomes and care efficiency is normally not in the hospital phase (where length of stay has already been driven down and there is generally no high cost procedure to streamline), but in the postacute period. If bundled payment is to make a positive difference for stroke patients, it must include secondary prevention and rehabilitation. Here, coordination is especially crucial." | 15.04.1 |
| "Our review suggests other common features of bundled payment that may be conducive to successful implementation:"…"(2) incentives aligned whereby physicians should not only be paid on the basis of compliance with meeting quality targets but also be given bonus if exceeding ambitious high-quality targets;..." | 15.02.3 |

| **ARTICLE 16: Miller, 2009 ^16^** | |
| --- | --- |
| **Selected Text Excerpt** | **Reference No.** |
| "A key problem with most capitation systems is that the amount of the payment is the same regardless of how sick or how well a provider’s patients are. This gives the provider a strong and undesirable incentive to avoid patients who have multiple or expensive-to-treat conditions, and it puts providers at risk of financial difficulty or bankruptcy if they take on large numbers of such patients." | 16.01.2 |
|  | 16.01.2.1 |
| "The decision about whether separate providers will have their payments “bundled” together into a single payment will depend in part on whether there is an organizational structure in place that can accept a bundled payment and divide it in a way that the individual providers find acceptable.14 Instead of waiting for such organizational mechanisms to emerge, particularly where hospitals and physicians have strained relationships, or limiting new payments to those providers that have them, proposals have emerged for “virtual bundling”—that is, paying providers separately but having each provider’s payments adjusted based on all providers’ joint performance." | 16.02.1 |
| "Physician practices trying to participate in a comprehensive care payment system face a different challenge, because a single payment designed to cover all services a patient needs would mean that the practice would have to be responsible not only for its own costs, but for paying claims to other providers for the costs of hospitalizations, diagnostic services, and so forth. Most providers do not have their own claims payment systems, and even with a good condition-adjustment system, a single high-cost hospitalization could cause financial problems. These problems could be addressed by using a “virtual” comprehensive care payment structure. For example, a physician practice could receive a single condition-adjusted payment for all of the services it provides directly to its patients, but the payer would continue to pay hospitals and other providers separately. (One version of this approach has been termed “comprehensive primary care payment.”)16 If the physician practice also receives a bonus or penalty payment based on the rate at which its patients use other services (such as hospitalizations or imaging services), the physician practice would still have an incentive to control total costs, but without being fully financially responsible for paying all providers. Another intermediate model would be for a physician practice to receive a single payment to cover all outpatient services its patients receive (including from other providers), with bonus/penalty payments based on inpatient service use." | 16.02.3 |
| "This is the method typically used by commercial health plans to determine how much they will pay providers. The outcome, however, depends on the relative size and level of consolidation of payers and providers in a particular regional market. Price setting by the provider; competition for patients based on value. Although this model is used in most other economic sectors, it is used rarely in health care, other than for services where consumers pay all or most of the cost of the service (such as for cosmetic or laser eye surgery). It would likely be easier to use the third option (market competition) under episode-of-care and comprehensive care payment structures than under fee-for service. Under FFS, even if consumers know the price of individual services, they do not know how many services will be used by different providers to treat them, so they cannot easily compare the relative value of different providers. In contrast, a single price for an entire episode of care or for an entire year of care would make such comparisons much easier." | 16.02.5 |
| "The first level of protection for patients is the use of a good condition adjustment system to ensure that sicker patients can receive more services. A growing number of these systems are available. 19 Beyond this, patients can be protected through techniques such as the following: (1) making outlier payments for patients requiring unusually high amounts of care; (2) including rewards or penalties for providers based on the outcomes of their care; (3) requiring that essential services be delivered for payment to be received; and (4) publicly reporting on quality measures, particularly for minority and disadvantaged populations." | 16.02.6 |
| "Aligning incentives across multiple payers. It is difficult for a provider to change the way patient care is provided, particularly when new staff or infrastructure are required, if only some patients are paid for under a new payment system. Moreover, there is the risk that instead of eliminating inefficiencies, providers will shift costs to payers who are still using fee-for-service systems. However, aligning multiple payers is challenging, because antitrust laws and policies at both the federal and state levels limit the ability of multiple payers to discuss and agree on changes in payment systems. To overcome this, state governments and nonprofit regional health improvement collaboratives are playing a growing role in forging consensus on new payment systems among multiple payers." | 16.03.1 |
|  | 16.03.2 |

| **ARTICLE 17: Porter & Kaplan, 2015 ^17^** | |
| --- | --- |
| **Selected Text Excerpt** | **Reference No.** |
| "As implemented in practice, however,the DRG system has serious flaws.  DRGs reimburse specific procedures or inpatient stays, not overall care for patient conditions.  DRGbased systems are often only for hospital payments, with separate payments made to physicians and suppliers, and none of them contingent on patient outcomes." | 17.01.1a |
| Bundled payments are also common for treatments that patients directly pay for, such as in vitro fertilization, plastic surgery, and Lasik eye surgery. In these situations, consumers demand a payment approach similar to how they pay for almost all other services they purchase. These examples provide clear evidence that bundled payments are feasible and effective." | 17.02.1 |
| "Some procedures and services (like  imaging) are extremely well reimbursed.  Others,  particularly patient consultation, patient education, and  services performed by primary care providers, are  reimbursed poorly, if at all.  Providers end up having to  crosssubsidize across their services lines. FFS leads to overinvestment in units that perform generouslyreimbursed services, creating excess capacity  and more pressures for supply induced demand for such  services.  And, it leads to underinvestment in  unreimbursed or poorlyreimbursed services, even those that contribute to better patient outcomes and that avoidmuch higher costs (e.g., emergency admissions and expensive adverse complications or recurrences) later in  the patient’s care cycle." | 17.01.1b |
|  | 17.02.2 |
| "For an acute condition, the bundled payment should cover the costs of all (i) clinical, staff, rehabilitation, and administrative personnel,  (ii) equipment and facilities, (iii) supplies, devices, implants, tests, imaging, and medicines used during the care cycle, and the necessary support services such as a billing, HR and IT, to enable the care to take place."..."For a chronic medical condition, the bundled payment should be time based ($ per month or year)."..."For primary care, bundled payments should also be time based, covering the full set of primary and preventative care services required for defined segments of patients with similar needs." | 17.03.1 |
|  | 17.03.2 |
|  | 17.03.3 |
| "The cost of dealing with complex complications may best be covered in separate bundles, although the chronic care team would be held accountable both clinically and financially for complication incidence, adjusted for patient risk." | 1703.4 |
| Figure 1: | 17.04.1 |
| "Time driven activity‐based costing (TDABC), derived from understanding the care processes used  to treat a condition, gives providers the ability to measure true patient‐level treatment costs (and to  reduce those costs over time).11  TDABC uses a twostep approach for accurately measuring the cost of treating a patient’s medical condition over a complete cycle of care.  First, a team of clinicians and  administrators maps all the clinical and administrative steps used during the care cycle, and identifies the specific resources (personnel, equipment, space, materials and supplies) used at each step as well as the estimated time spent.  Second, finance staff estimate the cost per available minute of each type of resource.  Total treatment cost is calculated by multiplying resource time by resource cost per minute at each step, summing up across all the steps in the care cycle, and adding in the cost of all purchased materials and supplies.12   The TDABC calculation provides the accuracy and transparency required toallow providers to be confident about their costs and, consequently, the margins they can earn from a bundled payment contract." | 17.05.1 |
| "A valuebased bundle should limit providers’ exposure to unexpectedly high costs from catastrophic and outlier cases by including a “stop loss” provision that caps the provider’s liability to a maximum payment, or coinsures for costs beyond a threshold.  By limiting the total exposure of providers to excessive or unforeseen risk, the bundled price need not include a risk premium to protect providers against catastrophic cases.  This is especially important when providers perform an insufficient number of cases to absorb such risk themselves."..."Bundled payments are linked to factors that providers can directly control – successful and comprehensive treatment for the patient’s medical condition – rather than ad hoc care for any medical need that arises.  Payers bear the actuarial risk of the incidence of the medical condition in their covered populations – the proper role for private and public insurance – while providers, appropriately, bear the medical risk and accountability for treating patients with specific medical conditions, with protection for truly catastrophic cases." | 17.04.2 |
| "We believe, however, that five primary reasons explain the current limited use of bundled payments: Inefficient organization of providers; Fragmented services with low volume by condition; Inadequate or absent information on outcomes by medicalcondition; inaccurate measurements of cost by medical condition; and Resistance to change by payers that have perceived benefits from complex to administer fee‐for‐service contracts." | 17.05.1 |
| "Systematic outcome measurement by medical condition, unfortunately, remains rare.  Most clinical teams do not collect information on outcomes nor do they know their actual risk of complications.  Payers also lack comparative outcomes data on which to measure and reimburse providers.  With insufficient valid information on patient outcomes, most bundled reimbursement experiments have contracted not on outcomes but on a narrow set of process and compliance measures.  Or, at best, they rely on generic, easy to measure outcomes, such as patient mortality or readmission rates.  As outcome measurement and reporting begins to grow rapidly, this constraint will be eased.  In fact, the move to bundles can accelerate widespread standardized outcome measurement." | 17.06.1 |
| "On the provider side, creating a bundle requires a team that includes clinical leaders that treat the medical condition, along with quality, and finance, and contracting personnel.  Bundled payments must reflect the processes, outcomes, and costs that clinicians can embrace and take accountability for.  Finance staff and contract administrators lack the specific expertise about medical condition treatments and outcomes tonegotiate by themselves.  Beyond sharing expertise, involving physicians promotes trust and understanding between clinicians and staff, enabling them to work collaboratively on value improvement. Physicians left on the sidelines resist the new payment model because the contracts often do not reflect the realities of their actual care or patient needs. For example, when the County of Stockholm developed its first bundled payment for totaljoint replacements, it formed a working group that included representatives from leading members in thecounty’s orthopedic department community." | 17.07.1 |
| "Bundled payments must account for the significant variations in the outcomes and costs for a condition dueto patient risk factors and comorbidities. Given that providers and payers often lack sufficient data to fully riskadjust bundles today, a practical way forward is to restrict the initial bundles to the large cohorts of patients withsimilar risk profiles for the specific medical condition, such as those below a specified age and those without severe complications or comorbidities. For joint replacement, the initial cohort could be those patients classified as ASA 1 or 2. The remaining patients would continue to be reimbursed by the existing payment mechanism until better data became available to risk‐adjust outcomes and the bundled payment itself." | 17.08.1 |
| "The bundled price and outcome targets established should be maintained for a multi‐year period, such as three years, to create incentives for providers to benefit from innovations that improve outcomes and lowercosts. Annual reductions in price or increases in outcome targets will work against provider acceptance of valuebased bundles, and lead to resistance in negotiations." | 17.09.2 |
|  | 17.09.1 |

| **ARTICLE 18: RAND, 2009 ^18^** | |
| --- | --- |
| **Selected Text Excerpt** | **Reference No.** |
| "Bundled payment is intended to decrease spending via several mechanisms: a eduction in the number of unnecessary physician services during a hospitalization, more judicious use of health care resources during the hospital stay, and a reduction in postdischarge costs, including unnecessary post–acute care services and avoidable readmissions (Medicare Payment Advisory Commission [MedPAC], 2008). If the costs of an episode of care are less than the bundled payment amount, the providers (hospital and physicians) can keep the difference; if the costs of care exceed the bundled payment, the providers bear the financial liability. Bundled payment systems can yield savings for payers if a discounted rate is negotiated at the outset or if payment amounts are adjusted downward to reflect the efficiencies achieved after the system is in place." | 18.01.1 |
| "One of the unintended consequences of reducing reimbursement surrounding a hospitalization is that it could result in a reduction in both necessary and unnecessary care. To minimize this possibility, we assume that quality of care standards would have to be met as part of the bundled payment system." | 18.02.1 |
| "Conditions or procedures with clear begin and end dates may be more feasible for a bundled payment system. Thus, the number of procedures or conditions that can be included in bundled payment will affect the potential to reduce spending."…"We do not know how a bundled payment system would influence spending for other types of procedures or for conditions with less tangible beginning and end points for services." | 18.03.1 |
| "Bundled payments for an episode of care are typically based on average costs for that episode with a planned reduction in payments for increased efficiency and reduced complications. To ensure that the payment amounts are fair, some adjustment for patient severity must be included in the calculations. If bundled payments do not take severity of disease into account, providers may not want to care for sicker patients because of the risk of financial losses. How well this aspect of the bundled payment system is designed will affect potential savings." | 18.04.1 |
| "The limited prior experience with bundled payment was conducted with hospitals and an integrated delivery system that were at baseline performing as better than average institutions. If the bundled payment system is initially voluntary, it is likely that high performing systems will be the first to sign up and they may have less room for improvement (and thus less potential for reducing spending) than systems that do not volunteer." | 18.05.1 |
| "This particular type of payment system is so new that the possible unintended consequences of implementing the policy are based more on generalizing from other experiences than from direct observations of bundled payment experiences. For example, providers may try to shift care beyond the post-acute period to increase reimbursement (similar to discharging patients more quickly from hospitals after the implementation of Diagnosis Related Groups [DRGs]). Adjustments for case mix severity could lead to the type of upcoding (i.e., patients coded as having more severe conditions in order to increase the reimbursement amount) seen in the DRG system, which could reduce expected savings. Providers may also try to increase the numbers of discrete bundles to maintain their income. Past bundled payment systems have not included post–acute care facilities in the bundle; including such facilities in the bundle would clearly affect decisions about when the patient was ready for transfer from the acute care hospital to another facility and how payments are distributed among providers." | 18.06.1 |
| "If a bundled payment approach for the reimbursement of multiple providers over an episode of care reduces the costs of care, we would expect that a portion of these cost savings might be passed on to the consumers. The savings will depend on the cost sharing features of a given health plan. For example, if patients were paying a fixed proportion of the medical bill, which in total is less, the cost to patients would also be less. If bundled payment is applied to a subset of procedures and conditions, only patients requiring those services would be likely to experience savings. The effect of this mechanism will vary with the cost sharing features of the health plan. Patients without any cost sharing, as with Medicaid, will experience no changes in their financial risk with this policy. Patients who pay a percentage of the contracted amount, on the other hand, will pay less under a bundled payment system." | 18.07.1 |
| "However, some hospitals might have an easier time cutting costs and improving efficiency in patient care than others. For example, the relationship between the hospital and its affiliated staff or the culture of collaboration within the organization could facilitate or impede the ability to make substantive changes in care patterns. In addition, bundled payment approaches may have differential effects in the context of different types of hospitalizations; complex hospitalizations could offer more opportunities for waste reduction than more straightforward ones." | 18.08.1 |
| "Bundled payment approaches are often proposed in tandem with other mechanisms, such as pay for performance, that aim to improve reliability. In Geisinger Health System's ProvenCareSM program, a bundled payment approach for reimbursement of coronary artery bypass graft (CABG) procedures was combined with the implementation of a set of 40 best practices. To improve clinical outcomes and reduce costs, doctors used national guidelines in the care of CABG patients to create checklists of best practices associated with the procedure. Staff had to use these checklists before, during, and after care to ensure that all of the best practice guidelines were being followed. At the beginning of the program, adherence to best practices was 59 percent; after the program began, performance rose steadily, reaching 100 percent at three months but then fell to 86 percent. Performance once again reached 100 percent at six months and stayed there for the remaining six months of the evaluation period (Casale et al., 2007)." | 18.09.1 |
| "Bundled payment to multiple providers could potentially improve patient experience through improved care coordination and simplification of billing procedures for patients. On the other hand, patient experience could decline if doctors have less time to spend with patients or if patients have fewer choices of physicians under a bundled payment approach." | 18.10.1 |
| "However, there is concern that, under a bundled payment approach, providers would cut back on appropriate care as well as on unnecessary care. Quality of care measures could be added to the bundled payment mechanism to minimize this possibility; however, some areas of clinical care do not have well established national guidelines and developing appropriate quality metrics in these areas may be more difficult." | 18.11.1 |
| "The implementation of bundled payment approaches would require a broad set of operational decisions. Bundled payment approaches pose significant operational challenges. First, the policymaker must decide what types of episodes of care are most appropriate for bundling. For example, the duration of an episode of care for a coronary artery bypass graft (CABG) or other surgical procedure is fairly straightforward to determine: A CABG has a fairly clear beginning and end, whereas the length of an episode for a chronic disease such as diabetes is arbitrary. Thus, this type of payment system may be easier to implement for some conditions and procedures than others. Although bundled payment provides an incentive to reduce complications, the payment system must account for differences in the illness severity of different patient populations. Thus, the adequacy of coding systems and case mix adjustment methodologies will pose operational challenges for many conditions and procedures. In the absence of adequate case mix adjustment, providers may not want to care for the sickest patients for fear of being financially liable for their inherently more expensive care. On the other hand, if the bundled payment amount is significantly higher for patients who are sicker or more complex, providers may try to code patients as being sicker. Mechanisms to monitor such practices will likely have to be made part of a new payment system." | 18.12.1 |
|  | 18.12.2 |
| "One challenge involves the stability of health insurance coverage. Whereas the Medicare population is stable in its source of insurance coverage, private insurance can have high turnover rates. If a patient is treated under a bundled payment system and changes insurance during the episode of care, it can be difficult to apportion the payment correctly. If a patient moves to a different geographic area during the period covered by the bundle, there would have to be a mechanism to ensure the fair distribution of payments." | 18.13.1 |
| "Bundled payment approaches may be significantly easier to implement in integrated delivery systems where contractual and collaborative relationships already exist between providers and facilities. In the absence of such preexisting arrangements, significant administrative structural changes would have to occur within participating entities. For example, hospitals are not typically in the position of having to reimburse physicians for services. If hospitals received the bundled payment and had to distribute it, they would have to set up contracting, billing, and reimbursement systems, all of which could require an expansion of current technologic capabilities. Further, mechanisms would have to be in place to guarantee that physicians were not being double paid (via the bundle and separate billing) for their services and that physicians seeing a patient for a problem unrelated to the bundled diagnosis were still paid appropriately. Determining rates of reimbursement would also be difficult. It can be technically challenging to match the appropriate rate with the current resource use at a given hospital. The bundle amount would have to reflect the costs of treatment per episode per patient and evolve to match the changing costs of providing treatment over time (MedPAC, 2008)." | 18.14.1 |
| "Further, mechanisms would have to be in place to guarantee that physicians were not being double paid (via the bundle and separate billing) for their services and that physicians seeing a patient for a problem unrelated to the bundled diagnosis were still paid appropriately." | 18.14.2 |
| "First, providers must be informed of their current resource use and rates of readmission surrounding a hospitalization and shown how these statistics compare with those of their peers. Second, payment changes should be implemented to encourage providers to collaborate on and coordinate care." | 18.15.1 |
| "Such changes might involve reduced reimbursement for hospitals with high readmission rates or mechanisms to reduce reimbursement to physicians who have high rates of referrals or who stint on care (i.e., provide inappropriately low levels of service). There could also be exploration of "virtual bundling," in which the payer (e.g., Centers for Medicare & Medicaid Services [CMS]) would calculate and distribute any gains or losses without actual agreements among providers. Third, a pilot program could be used to test a bundled payment system. In the pilot, providers could choose to receive a single payment for all Medicare Part A and Part B services surrounding a hospitalization (including a post-acute period of 30 days) (MedPAC, 2008)." | 18.16.1 |
|  | 18.16.2 |
| "Note that many providers opposed the demonstration. They raised concerns about the government designating some providers as "high quality," with an associated increase in payment. Despite this concern, a large number of hospitals applied to participate (MedPAC, 2008)." | 18.17.1 |
| "Finally, whereas current hospital care involves separate claims from multiple providers, a bundled payment approach involves only a single payment for an entire episode of care and could potentially lead to a more efficient billing process." | 18.18.1 |

| **ARTICLE 19: Ridgely et al., 2014 ^19^** | |
| --- | --- |
| **Selected Text Excerpt** | **Reference No.** |
| "The IHA also convened technical committees whose members were specialist physicians and representatives of health plans or hospitals. The technical committees were tasked with defining the bundles—that is, specifying which services were included in a bundle and which were excluded. Through the committee consensus process, it was determined that the IHA episode definitions for total knee replacement and total hip replacement would include “facility, professional and medical implant device charges for the inpatient stay; a 90-daypost-surgicalwarrantyforrelatedcomplications and readmissions are [sic] included, but other post-acute care is excluded.”6 The IHA and its consultants developed model contract provisions that could be used in negotiations between the health plans and hospitals. However, because of antitrust concerns, the IHA was not involved in price or contract negotiations. The parties were free to modify any of the model contract provisions except for the bundle definitions. If the parties happened to be negotiating the overall preferred provider organization (PPO) contract at the time, the bundled payment arrangements were folded in. Otherwise, bundled payment negotiations were undertaken separately." | 19.01.1 |
| "Of the eight hospitals that originally indicated an interest, only two eventually signed contracts with PPO plans. Hospitals dropped out because of a perceived lack of need for the pilot in their own institutions and concerns about the time and effort involved." | 19.01.2 |
| "One representative of a physician organization explained: “It definitely went longer than any of us thought. We felt it was important to have consistency…[but] when you peel back the onion, you find things that complicate this, and it can take a longtime. When these things take a long time, people tend to lose interest and start to think it’s never going to happen.” Parties also had difficulty reaching consensus about what exclusions (for example, preexisting comorbidities and other potential risk factors such as high body mass index) would apply to the bundle definitions." | 19.02.1 |
| "Parties also had difficulty reaching consensus about what exclusions (for example, preexisting comorbidities and other potential risk factors such as high body mass index) would apply to the bundle definitions. Some stakeholders felt that negotiation over potential exclusions entailed gamesmanship on the part of early participants. For example, representatives of health plans told us that they were interested in broad inclusion criteria that would expand the number of patients potentially covered under each bundle. They also favored more expansive bundle definitions to lengthen the time of the episode and include more services (such as presurgical care and post acute care). In contrast, hospitals and surgeons generally sought to narrow the inclusion criteria (to include only the lowest-risk patients) and to limit the length of the episode, since including preadmission or post discharge services would increase the need to coordinate care among a greater number of providers." | 19.02.2 |
| "Reflecting these diverging basic interests, the resulting definitions were quite conservative in terms of both patients and services included. In the pilot, the hospitals and surgeons prevailed: For example, the bundle definitions excluded obese patients (those with a body mass index greater than 40) and post acute and rehabilitation services." | 19.03.1 |
| "An additional complication was providers’ concern about the unprotected financial risk that they would incur if they operated on higher risk patients, given the decision not to risk adjust bundled payments (that is, not to adjust payments to account for the fact that some patients will be more expensive to treat), create stop-loss measures (which provide protection against unpredictable and catastrophic loss), or require the purchase of reinsurance in the contract (which involves transferring some of the risk to a third party). In the end, almost all stakeholders agreed that the bundle definitions, which represented a compromise among the parties, proved to be too narrow to capture an adequate number of procedures to make bundled payment viable." | 19.03.2 |
|  | 19.03.3 |
|  | 19.03.4 |
| "An early challenge facing the IHA pilot was the conflict in how health plans and providers viewed pricing for the bundles. Health plans expected to negotiate price reductions compared to what the hospitals were being paid under fee-for service. However, hospitals were concerned about implementation costs and the increased financial risk they would assume under bundled payment (for example, being responsible under a warranty for the cost of treating complications such as a readmission and treatment for a surgical site infection). As a consequence, some sought a higher level of payment than they received under fee-for-service. Differing views persisted in negotiations between the parties and at times rendered the negotiations difficult and slow." | 19.04.1 |
| "To the extent that hospitals and physicians were willing to take risks and offer warranties under bundled payment, the hospitals wanted the health plans to steer higher patient volume to their facilities. They wanted health plans to do this either through benefit design changes such as lower out-of-pocket costs or by designating the facilities as centers of excellence. However, to do so would have required the health plans to file new benefit options for regulatory approval, making these changes impractical within the pilot period. As a result, the IHA project did not include incentives for consumers to use the participating hospitals. This lack of steering of patients greatly reduced hospitals’ enthusiasm to participate." | 19.05.1 |
|  | 19.05.2 |
| "Seeing the low volume, health plans decided that they would have to process claims manually. This made it impossible to test automated processes for paying or denying claims that were submitted to the insurer within the pilot. In addition, at least initially, hospitals lacked the capability to pay physician claims under the bundle. The IHA approached two technology vendors to help. Within a year, one vendor had developed a new software program for hospitals to pay physician claims." | 19.04.2 |
|  | 19.04.3 |
| "In the words of a participant representing the state regulatory agency: “The concern with bundled payment is that if you’re paying an intermediary who is making the payment to each downstream provider, what happens if the intermediary doesn’t pay the provider? …Our concern is if the intermediary doesn’t pay the downstream provider, is the health plan still on the hook for that payment? If [the health plan] is, that risk arrangement isn’t a particular concern for us. If the plan says, ‘It’s the intermediary’s problem,’ then we have a concern. That intermediary would seem to be functioning in some sort of insurer-like capacity.”" | 19.05.3 |
| "Find Acceptable Methods For Managing Risk. Providers and payers understandably have competing interests in the distribution of financial risk. In the IHA pilot, provider risk was mitigated by excluding higher-risk patients, which contributed to lower volumes. Alternative methods such as risk adjustment and stop-loss protection could be used to limit risk without decreasing volume. Ideally, bundled payment programs should incentivize providers based on performance risk (that is, factors that are within their control) and adjust or control for insurance risk (factors that are outside of providers’ control)." | 19.05.4 |
|  | 19.05.5 |
|  | 19.05.6 |
| "In addition, the Department of Managed Health Care was concerned about issues of consumer cost sharing. To preempt regulatory problems, the health plans decided to require patients to pay the lesser of what they would have paid on the fee-for-service bills and the nominal coinsurance rate due under bundled payment. Although the IHA provided model contract templates, each of the health plans had to negotiate approval of its own contracts with the department. The first contract took approximately nine months from submission to approval. The time delay and uncertainty associated with obtaining regulatory approval for contracts added significantly to the delay experienced by the pilot participants." | 19.06.1 |

| **ARTICLE 20: Shih et al., 2015 ^20^** | |
| --- | --- |
| **Selected Text Excerpt** | **Reference No.** |
| "Cardiovascular care is the arena in which implementation of bundled payments is arguably most visible and may be most impactful. Many previous demonstrations of bundled payments have concentrated on cardiovascular conditions, and it is likely that future efforts will continue to do so, with good reason. First, cardiovascular diseases are common, costly, and deadly1 and therefore are important in national discussions for healthcare reform. Second, care for cardiovascular disease involves multiple providers from different disciplines (primary care, cardiology, cardiac surgery, anesthesiology, radiology). Lastly, cardiovascular patients receive care in multiple healthcare settings (hospital, outpatient primary care and subspecialty clinics, skilled nursing facility, etc). Given all these factors, bundled payments have the potential to substantially improve care coordination and to generate savings for cardiovascular care." | 20.01.1 |
| "Previous studies illustrating large variation in healthcare costs associated with index hospitalization, physician services, readmissions, and post acute care have highlighted the potential for cost savings with bundled payments." | 20.02.1 |
| "To set a target price, payers often look at overall variation and mean pricing in historical payments for all facets of an episode of care to establish a case rate. Payers then enter into negotiations with providers to set a target bundled price, sometimes 1% to 2% below the case rate or below projected spending growth. Under this model, a participating provider is incentivized to provide efficient care, reducing the number and cost of services contained in the bundle." | 20.03.1 |
|  | 20.03.2 |
| "The potential for savings for payers lies in upfront discounted payments for episodes of care, as well as shared savings with providers when costs fall below the lump-sum payment. " | 20.04.1 |
| "Bundled payments are a middle ground in the spectrum of healthcare payment models (Figure). On the one hand, they are a considerable shift from the traditional fee-for-service model, in which providers are reimbursed separately for each distinct service provided. Yet, importantly, bundled payments are not representative of global payments, or capitation, in which a healthcare system is paid a lump-sum payment per attributed patient over a distinct time period, regardless of the number of distinct episodes of care." | 20.05.1 |
| "Finally, it also is possible that academic health centers may be disadvantaged by bundled payments. In addition to patient care, these centers prioritize research and teaching. Without consideration of this concern and special payments or other allowances to address it, academic centers may be negatively affected by bundled payment agreements." | 20.06.1 |

| **ARTICLE 21: Sutherland et al., 2012 ^21^** | |
| --- | --- |
| **Selected Text Excerpt** | **Reference No.** |
| "Thirdly, unilateral hip and knee replacement are a relatively uncomplicated patient population well-suited as a test for a bundling approach. Only 1.6% of knee replacement patients and 2.9% of knee replacement patients were long length of stay outliers. In addition, for these surgeries, the morbidity burden is lower than the general population of hospitalized patients; 4.9% of the patients in this analysis had significant co-morbidities, compared to an average 18.9% among the population of acute inpatients in Ontario." | 21.01.1 |
| "There are gaps in the Ontario data: the most relevant to this study includes inconsistently reported outpatient services, unobserved post-hospital drug utilization data (expected to be small for the conditions selected in this analysis) and privately paid health care, such as supplemental therapy services. Due to the length of wait times in Canada – currently around 200 days for elective knee replacement – pre-hospitalization costs, such as preoperative counseling, could not be clearly delineated from utilization records." | 21.02.1 |
| "To develop the hip and knee replacement ‘bundles’, we obtained retrospective access to extensive cross-sections of clinical and administrative episodic datasets for the population of residents in the Province of Ontario for 3 (fiscal) years, 2007 to 2009. Each person in Ontario has a unique individual identifier which moves with them across care setting and facilitates longitudinal analyses of cross-sectional data sources. In our analytic datasets, the linking variable is anonymous and the data are used with permission of the Ontario MOHLTC." | 21.01.2 |
| "While bundled payments may be a sharp contrast to existing funding approaches in Ontario [24], implementation for select CCGs could proceed on a graduated basis. First, existing data could be leveraged to mimic the ‘virtual bundles’ in this analysis. Bundled CCG costs for individual hospitals and LHINs could then be published and compared with provincial reference costs, or with the costs of benchmark providers demonstrating high quality and high efficiency. Importantly, while Ontario physicians are not currently under the funding authority of LHINs, they must be included as a crucial element in this process as their decisions influence a large proportion of hospital-borne costs." | 21.02.2 |
|  | 21.02.3 |
| "While bundled payments may be a sharp contrast to existing funding approaches in Ontario [24], implementation for select CCGs could proceed on a graduated basis. First, existing data could be leveraged to mimic the ‘virtual bundles’ in this analysis. Bundled CCG costs for individual hospitals and LHINs could then be published and compared with provincial reference costs, or with the costs of benchmark providers demonstrating high quality and high efficiency. Importantly, while Ontario physicians are not currently under the funding authority of LHINs, they must be included as a crucial element in this process as their decisions influence a large proportion of hospital-borne costs. In a second stage, widespread collection, reporting and measuring of health and functional status measures could proceed; health and functional status measures are currently being piloted across populations. With validated measures of improvement in health and functional status in hand, funding amounts could be refined to create incentives (or disincentives) for changes in volume or homogeneity of clinical outcomes. For instance, sustained large gains in health should attract additional funding for a particular network of providers." | 21.03.1 |
| "In addition to establishing the methodological parameters for defining and pricing bundled payments, a key implementation challenge concerns the administrative and contractual arrangements required to implement and distribute a single payment across multiple independent provider organizations. The Integrated Healthcare Association of California proposes that one health organization – either a hospital or physician organization – act as a general contractor, negotiating subcontracts with other providers for services included in the episode definition [42]." | 21.04.1 |
| "It should be noted that while this paper describes the construction of CCGs in some detail for unilateral hip and knee replacement, the development of CCGs may not be robust for all types of patients. The criteria for building CCGs may rest on volume, policy relevance, number of providers involved and degree of homogeneity within a type of episode [24]. For example, while normal vaginal delivery may be high volume and represent significant costs, these hospitalizations tend to require little post acute care and consequently do not require incentives to improve coordination between sectors. On the other end of the spectrum, hospitalizations for bipolar disorder may have diffuse trajectories of care or may link to non-reported community-based services. In other settings, complex care needs may require longer follow-up periods beyond 30 days [36] or validated process and outcome measures of quality to monitor ‘skimping’ on care. As described above, unilateral hip and knee replacement represent a relatively uncomplicated population to prototype a bundled payment arrangement in Ontario; the general approach will require adaptation to the unique characteristics of other patient populations." | 21.03.2 |

| **ARTICLE 22: Berenson et al., 2016 ^22^** | |
| --- | --- |
| **Selected Text Excerpt** | **Reference No.** |
| "All payment methods have strengths and weaknesses, and how they affect the behavior of health care providers depends on their operational design features and, crucially, on how they interact with benefit design. Those seeking greater value for their health care dollar are also turning to innovation in benefit design, which also typically involves the implementation of more than one approach at a time—each with its own strengths, weaknesses, and effect on consumer health care behavior. Although payment and benefit design each has received significant attention independently, the intersection between the two has received little if any." | 22.01.1 |
| "Further, too much of the discussion of payment reform has focused on payment models’ theoretical effects rather than on their interactions with other payment methods. We must also consider interaction with an array of benefit designs that either encourage or frustrate the opportunities for payment reform to improve value." | 22.02.1 |
| "Payers, including Medicare, have recently recognized they can create new fee schedule codes to reward evaluation and management activities that had never been specifically paid, including complex chronic care management and activities related to patients’ transitions from hospitals to community-based or other post-acute settings. Paying for some important services (e.g., routine phone calls and email communications) on a fee schedule is challenging, because the transaction costs of billing and receiving might be more costly than the service itself. However, a range of other activities might be amenable for inclusion on a fee schedule." | 22.02.2 |
| "The episode of care has two dimensions: a clinical dimension, which can represent either the set of services or the clinical conditions that compose the episode, and a time dimension that reflects the beginning and the end of the episode." | 22.02.3 |
| "In essence, the approach is designed to transfer financial responsibility for the technical risk (i.e., risk related to care production) that is under the included providers’ control, but not the probability (or insurance) risk that relates to the burden of illness and injury in any large patient population. The bundled providers—clinicians and facilities—have common financial incentives to control the cost of the bundle, because they keep the savings or bear the cost of overruns if costs differ from the fixed payment." | 22.02.4 |
| "Compared to procedure-based episodes, bundled episodes for conditions could affect much more health care spending and could create much stronger incentives for care coordination across health professionals and providers. Condition-based bundled episodes also could counter the volume-inducing incentives of procedure-based episodes, as discussed below. However, particular challenges associated with chronic condition-based episodes must be addressed—particularly for patients with multiple chronic conditions." | 22.03.1 |
|  | 22.03.2 |
| "Bundled episodes can be viewed as partway between volume-based payment and true population-based payment (e.g., global capitation), allowing clinicians and organizations to ease into broad payment reform with increased accountability for quality and costs."..."Bundled episodes are more consistent with the service-line strategies hospitals have adopted over the past decade than with population-based payment approaches. Thus, the approach could be more readily undertaken without major change in business models and complex organizational cultures." | 22.04.1 |
| "Procedure-based bundling remains firmly a volume-based payment method in that it rewards providers for initiating more episodes. The approach might result in high-quality, efficiently produced, but unneeded procedures." | 22.05.1 |
| "Hospitals and other providers have a logical impulse to narrow their referrals to favored post-acute care providers, which might compromise patients’ choice of provider." | 22.06.1 |
|  | 22.06.2 |
| "Where health professionals and other providers, such as hospitals, remain legally independent, sustaining relationships among multiple providers may be difficult. Of particular concern is that a hospital, typically the dominant cost center in a procedure-based bundled episode, may dominate the collaboration and act in its own interests, which may not be congruent with the others’." | 22.07.1 |
|  | 22.07.2 |
| "Even assuming procedure-based bundling is successful, hospitals and physicians in noncompetitive markets may be able to increase volumes and prices for other services to make up for reduced revenues on the bundles. This is more likely if basic payment, except for the bundled episodes, remains volume-based through fee schedules for physicians and either per diems or DRGs for hospitals." | 22.08.1 |
| "Giving an entity funds that otherwise would flow directly to a different provider via a prospective payment raises concerns. One approach is to continue paying individual providers in a bundle separately and retrospectively, using standard payment methods that reconcile payments at the end of the episode (and perhaps withholding a portion of payments to cover overspending, if that occurs). In this case, the collaborative group would only have to agree on how to distribute surpluses or pay back deficits, while a flow of core funding for each entity would be assured. However, the approach of maintaining separate payment streams may to some extent undermine the goal of true collaboration across health professionals and other providers." | 22.09.1 |
|  | 22.09.3 |
| "Many conditions—even common ones—are not well defined, offering providers an incentive to “find” conditions in order to receive a prolonged payment for a condition-specific episode. Current variations in ICD diagnosis coding, even for common conditions such as congestive heart failure, suggests a lack of standardization with the potential for gaming (although definitions of conditions for episodes are improving)." | 22.10.1 |
| "Alternatively, holding a particular provider who has accepted payment for a particular condition-based episode accountable for total health spending as in Medicare’s BPCI initiative can generate conflicts among different physicians caring for different conditions." | 22.11.1 |
| "To address the concern that clinicians will make questionable diagnoses to trigger an episode payment, strict criteria can be required. These criteria can include demonstrating positive test results or providing patients with multiple encounters and documenting diagnoses via claims forms (thus demonstrating a minimum level of persistence or verification of the diagnosis by one or more other clinicians)." | 22.12.1 |
| "A specific concern is that putting hospitals, clinicians, and, perhaps, post-acute care facilities together into a recognized “focused factory” could produce, in effect, a “bargaining unit.” This could raise prices higher than they would be if the parties were negotiating separately, without their ongoing joint participation in providing services." | 22.13.1 |

| **ARTICLE 23: Wojtak & Purbhoo, 2015 ^23^** | |
| --- | --- |
| **Selected Text Excerpt** | **Reference No.** |
| "We considered two different ways to approach bundled payment for patients in the community: 1. Condition-based: Identifying opportunities in which there is a defined group of patients with a simple, clearly defined treatment goal; a single or uncomplicated diagnosis; and an evidence-informed treatment pathway, such as hip and knee replacement surgery followed by rehabilitation or less complicated surgery followed by post-surgical wound care. Under this type of bundled payment model, accountability for quality and cost could rest with a single organization that provides all the care, with a lead “most responsible provider” organization that either partners with or sub-contracts for any supplementary services that it requires from other organizations or sectors, or with shared accountability across two or more organizations. 2. Population-based: For patients with multiple complex and chronic health conditions such as a frail older adult with dementia, congestive heart failure (CHF) and chronic obstructive pulmonary disease (COPD), bundled payments are a more challenging proposition. The challenges stem from the need for integrated care pathways that consider multiple providers involved in care combined with the heterogeneity of complex/chronic client populations in the community. We recommend a two-step process by first “bundling care” across our multi-provider home care model and with other sectors before establishing bundled payments. While there is more evidence for and experience with condition-based bundled payments, we believe that implementing population-based bundled payments shows greater promise for achieving higher quality and cost savings for the system, as long as we are able to overcome the structural challenges posed by the current home and community care delivery model and the rest of our fragmented health system." | 23.01.1 |
|  | 23.01.2 |
| "where cost savings have bee n achieved, the y are related to physician care and higher cost components of care delivery (we found no strong evidence in the literature for cost savings in home care or long-term care); and • jurisdictions that already have a higher degree of integration in their systems, like the Netherlands or in US-based integration models, are more likely to have made progress on bundled care for complex patient populations." | 23.02.1 |
| "Bundled payment cannot be seen as merely a funding or payment approach; it needs to be part of a larger strategy to align care and establish relationships that bridge across different provider organizations." | 23.01.3 |
| "As identified in the literature evidence, the likelihood of success for bundled payment systems increases when providers are centralized, integrated and able to coordinate and deliver seamless care for the full episode across the care continuum." | 23.02.2 |
| "The result of this test also supports the literature evidence that reimbursement based on expected costs for a defined episode(s) of treatment can work well in a situation in which there is a simple, clearly defined treatment goal; a single diagnosis; treatment by a single provider; and an accepted and expected treatment pathway." | 23.03.1 |
| "Although the literature does not specifically state that bundling for a single condition is preferable to multiple conditions, conditions suited to bundled payment tend to meet the following criteria: (1) prevalent across the population served and/or expensive to payers, (2) have limited variation in costs across patients with the same characteristics and (3) “have evidence-based clinical care guidelines” (Dobson et al. 2012: ES-4). Hussey and colleagues (2009) recognized that given the volume of clinically complex patients with co-morbidities, bundling payments for a single condition may not be an ideal approach, as managing their multiple conditions is interrelated (Hussey et al. 2009). Covering multiple conditions would result in more heterogeneity and greater financial risk to providers." | 23.03.2 |
| "While bundled payment for complex, chronic conditions in home care is our desired outcome, we believe a more practical and necessary place to start is with bundling care, in other words, creating a much more integrated team that bridges the multi-faceted service delivery model in home care and across other sectors." | 23.04.1 |
| "In addition, we have introduced a number of innovations into the model to improve care to clients and caregivers, and to support team functioning, including daily 15-minute virtual team huddles, a single phone number, and joint assessments by multiple team members. Although team members come from seven different organizations, the goal is for patients, caregivers and the rest of the system to experience our community-based palliative support as a single team." | 23.05.1 |

| **ARTICLE 24: Joynt Maddox et al., 2017 ^24^** | |
| --- | --- |
| **Selected Text Excerpt** | **Reference No.** |
| "Inherent in these models are choices around program scope (broad vs. narrow); selecting absolute or relative performance targets; rewarding improvement, achievement, or both; and offering penalties, rewards, or both. We examined and classified current Medicare payment models—the Hospital Readmissions Reduction Program (HRRP), Hospital Value-Based Purchasing Program (HVBP), Hospital-Acquired Conditions Reduction Program (HACRP), Medicare Advantage Quality Star Rating program, Physician Value-Based Payment Modifier (VM) and its successor, the Merit-Based Incentive Payment System (MIPS), and the Medicare Shared Savings Program (MSSP) on these elements of program design and reviewed the literature to place findings in context. We found that current Medicare payment models vary significantly across each parameter of program design examined. For example, in terms of scope, the HRRP focuses exclusively on risk standardized excess readmissions and the HACRP on patient safety. In contrast, HVBP includes 21 measures in five domains, including both quality and cost measures. Choices regarding penalties versus bonuses are similarly variable: HRRP and HACRP are penalty-only; HVBP, VM, and MIPS are penalty or bonus; and the MSSP and MA quality star rating programs are largely bonus-only. Each choice has distinct pros and cons that impact program efficacy." | 24.01.3 |
|  | 24.01.4 |
|  | 24.01.5 |
|  | 24.01.6 |
|  | 24.01.7 |
|  | 24.01.8 |
| "We also examined one large voluntary APM, the Medicare Shared Savings Program (MSSP).9 The design choices made for these programs have been widely divergent…" | 24.01.9 |
| "There are likely tradeoffs between a broad versus narrow scope for the performance measures included in a program. Programs that use a broader set of measures may spur providers to undertake more intensive systems-based approaches to overall quality improvement. On the other hand, targeted programs may be less administratively burdensome and could make critical areas for improvement especially salient." | 24.02.1 |
|  | 24.02.2 |
| "Another important issue is that of B teaching to the test,^ namely, whether when only a limited number of outcomes are measured, others—which may be equally important to patients and clinicians—are neglected. In the UK program mentioned above, for example, quality measures that were not specificallyincentedslowedintheirimprovement.15 Interestingly, in the same program, performance remained high for some incentivized quality indicators even after the indicators were retired,16 a pattern that has also been seen in an incentive program in US Veterans Affairs hospitals.17 This suggests that phasing a broad set of measures in and out rather than choosing a small static set may be a useful strategy." | 24.03.1 |
|  | 24.03.2 |
| "The appropriate scope of measures for a given program will depend on its goals. As noted above, the HRRP and HACRP have a specific thematic focus, and thus a narrow set of measures is appropriate. In contrast, HVBP, MSSP, and VM are programs intended to change the way care is delivered across conditions, and thus a broader set is necessary. One strategy might be to have specific, targeted programs for the highest-priority conditions or issues and broad-based, frequently updated programs to improve care more generally." | 24.04.1 |
|  | 24.04.2 |
|  | 24.04.3 |
| "Absolute benchmarks give providers specific targets to meet, which may be more meaningful to clinical leaders and frontline staff and may encourage collaboration across providers. On the other hand, relative benchmarks may feel more abstract and discourage collaboration. One criticism of the HRRP has been that its relative benchmarks mean that even if all hospitals improve their readmission rates, the majority will still receive penalties;" | 24.05.1 |
|  | 24.05.2 |
| "Relative performance assessment allows the payer to prospectively assure budget neutrality by ensuring that the number of B winners,^ or at least their winnings, can balance losses by the B losers,^ while absolute benchmarking has much less financial certainty. Relative benchmarking may also be more easily implemented because it allows the distribution of observed performance to determine rewards and penalties and does not require a significant duration of pre-data with which to set parameters for expected performance." | 24.06.1 |
|  | 24.06.2 |
| "Whether a program rewards improvement or achievement can significantly impact which providers do well and poorly under the program. If providers are evaluated only on achievement, the highest-performing providers at baseline will likely do best.18 For example, under the achievement-only HRRP, the highest-performing hospitals at baseline were the most likely to avoid penalties. The lowest-performing hospitals actually improved more quickly over the first 3 years of the program, but many still received penalties in every program year because they started out far behind the best performers anddidnotfullycatchup.13,14Baselinelowperformers,onthe other hand, may benefit the most from improvement opportunities. The purest form of rewarding improvement, evaluating providers against their own historical performance, may give baseline poor performers the best opportunity, assuming that there is Blow-hanging fruit^ that can be addressed. Early experience from the MSSP as well as the Pioneer ACO program suggests that the most expensive baseline providers were the most likely to save money, supporting this possibility.19,20 However, only rewarding improvement may also mean giving financial rewards to providers who have improved, but are nonetheless delivering suboptimal or even substandard care, or, on the other hand, failing to reward persistently excellent performers whose year-upon-year performance changes little." | 24.07.1 |
|  | 24.07.2 |
|  | 24.07.3 |
| "Another related issue is that of risk adjustment. Improvement-based comparisons depend much less heavily than achievement-based comparisons on accurate risk adjustment to enable fair comparisons between peers since each hospital or clinician serves as its own comparison group. This may be of particular salience to providers that serve medically or socially complex populations, who have been shown previously to perform more poorly on many existing VBP programs, in part because of characteristics of the patients they serve." | 24.08.1 |
| "Transparency for consumers is also a key consideration in the achievement versus improvement debate. If providers are only judged on improvement, a patient viewing a hospital’s rating might not know whether a good score was based on high absolute performance or on poor performance with high improvement over time. Given prior evidence suggesting that public reports may influence consumer choice in meaningful ways,25,26 such considerations are key. Public reporting and financial rewards could be de-coupled to avoid this particular problem." | 24.09.1 |
| "Some combination of rewarding achievement and improvement may be optimal in most cases, which is how many current programs, including HVBP, are constructed. This offers an incentive to organizations to participate even if initial performance is low, while also recognizing high absolute levels of achievement and acknowledging that continued improvement is relatively more difficult at high levels of performance. In addition, including some absolute measures would likely help consumers directly compare provider quality, increasing transparency and promoting consumer-driven care. Some data suggest that providers may prefer this dual approach as well27 and may respond better to mixed-strategy compensation models than single-strategy ones" | 24.10.1 |
| "There are pros and cons to the use of penalties versus bonuses. Prospect theory holds that more value is placed on losses than on equivalent gains (B loss aversion^),30 suggesting that penalties may provide a more powerful behavioral incentive than bonuses. Another related concept is that B willingness to accept^ is often significantly greater than B willingness to pay,^ suggesting that people require much more to give something up than they would be willing to pay for it.31 While there are few data in this area directly related to payment models, one hospital pay-for-performance program applied prospect theory by sending an advance incentive payment to eligible providers based on the expectation that they would be more motivated to avoid losing the payment than achieving a possible gain.32 Penalties may also be more economically efficient than bonuses, since bonus programs require paying additional money to high performers in order to incent change among low performers." | 24.11.1 |
|  | 24.11.2 |
|  | 24.11.3 |
| "A related question is the size of the incentive. Historically, bonus payments to hospitals have been in the range of 1–5% and to physicians 5 to 10%, but we know of no evidence that clearly links size of incentive to behavioral response. A large pay-for-performance program in UK hospitals that offered up to 4% bonuses was associated with improvements in mortality,38 while the Hospital Quality Incentive Demonstration (1– 2% bonus, 1–2% penalty)39 and HVBP programs (1–2% bonus,1–2%penalty)intheUSwerenot.11 This finding might suggest that larger incentives have a larger effect on performance, but again there are other differences between these programs that preclude firm conclusions based on these examples alone." | 24.12.1 |
| " Evaluating program design options therefore requires an understanding of not only the design elements themselves, but also of program genesis and intent." | 24.13.1 |

| **ARTICLE 25: Kline et al., 2017 ^25^** | |
| --- | --- |
| **Selected Text Excerpt** | **Reference No.** |
| "This 6-month episode length is based on analysis of CMS claims data that showed that expenditures for Medicare beneficiaries treated with chemotherapy for cancer peaked in the first 2 months after chemotherapy initiation before stabilizing after 4 to 6 months.7 These observations suggested that 6-month episodes were most likely to capture discrete treatment courses across a range of cancer types. Beneficiaries who receive further chemotherapy after the completion of a 6-month episode are eligible to begin additional OCM episodes. In analyzing spending patterns over time, CMS considered terminating episodes on the basis of observed gaps in chemotherapy receipt but chose not to use this strategy for several reasons. These included the absence of a gap in some patients as well the complexity of developing a comprehensive risk adjustment methodology that allowed for variable episode duration across and within cancer types." | 25.01.1 |
| "CMS considered other events that occur earlier in the cancer treatment process, such as the diagnostic surgery, as potential episode initiators. However, these earlier clinical events introduced greater expenditure variability into the model and offered limited opportunities for quality improvement in the process of care, because they often occurred before the involvement of the medical oncologist. In addition, beginning episodes before the initiation of chemotherapy would also have the disadvantage of dividing primary accountability for the patient’s episode between the surgeon and the oncologist." | 25.02.1 |
| "In addition, CMS anticipates that nearly 200,000 episodes will be initiated at participating PGPs during each performance year of OCM; this volume makes case-by-case determination of related services impractical. For these reasons, CMS decided that the most feasible and appropriate methodology to bundle payment for services included in the OCM episode and to set episode prices was to include expenditures for all Medicare Parts A and B services and, for beneficiaries with Part D, those Part D services not paid on a capitated basis (the Low-Income Subsidy and80%of the Gross Drug Cost Above Catastrophic Threshold). Although some CMS models exclude the cost of certain medical conditions not believed to be related to the primary diagnosis, these exclusions engender their own set of complexities and were not believed to be feasible given the breadth and complexity of cancer care. For this reason, a total cost of care model was selected. Total cost of care means that some acute events out of the oncologist’s control, such as motor vehicle–related injuries or trauma or adverse health events from comorbid conditions, may lead to costs that accrue to an episode’s expenditures. The cost of these services could increase episode expenditures but, to the extent that these rare, high-cost events also randomly occurred in the historical baseline period, they have been incorporated into the risk-adjusted benchmark episode price methodology (see Practice Expenditure Variation, Risk Adjustment, and Benchmark Episode Prices)." | 25.03.1 |
| "Its main disadvantage is its retrospective nature. This prevents practices from knowing which beneficiaries are attributed to them until after the completion of the episode and delays episode-specific feedback to the practices until the care episode is complete." | 25.04.1 |
| "Any model with financial risk for services automatically creates some incentive to shift high-cost beneficiaries to other providers. CMS observed that complex billing arrangements might create the potential for PGPs to shift high-cost beneficiaries to affiliated nonparticipating TINs. As previously stated, to reduce this possibility, CMS decided that any participating PGP is required to include in its OCM participation all practitioners who furnish chemotherapy services and all locations at which such services are furnished." | 25.05.1 |
| "Expenditures for health care in the United States continue to increase. In some clinical areas, such as oncology, expenditures have increased faster than the overall expenditures for health care.11 Retrospective payment analysis of historical episodes used to set disease-specific benchmark episode prices must include a methodology for adjusting these benchmark episode prices over time so that they accurately reflect current expenditures for patient care, while maintaining the incentive to provide high-value care." | 25.06.1 |
| "Because risk adjustment may not fully account for the significant variability in oncology expenditures, OCM participants have the option to minimize financial risk by choosing one-sided risk for the duration of the model. Under the one sided risk arrangement, benchmark episode prices will be subject to a larger CMS discount percentage than under the two-sided risk arrangement (4% compared with 2.75%), but participants will not be required to pay CMS in the event that aggregate actual episode expenditures for all episodes attributed to their practice in a performance period exceed the sum of the target prices for these episodes. Participating PGPs also have the option to elect two-sided risk beginning in 2017. The two-sided risk arrangement of OCM is an Advanced Alternative Payment Model under the Quality Payment Program, and practices selecting the two sided risk arrangement may qualify for incentive payments under the Quality Payment Program for sufficient participation in OCM.12 Under the two-sided arrangement, participants are required to pay CMS if aggregate actual episode expenditures for episodes attributed to their practice in a performance period exceed the sum of the target prices for those episodes. Under the two-sided risk arrangement, a stoploss provision limits the amount that any participant will be required to pay back to CMS within a given performance period to 20% of the sum of the benchmark episode prices for all episodes in the performance period. Regardless of the risk arrangements, CMS will cap a participant’s PBP for reducing expenditures below the target at 20% of the sum of the benchmark episode prices for all episodes in a performance period, which is consistent with stop-loss limits in other CMS EPMs. In addition, participating PGPs in the one-sided risk track that do not achieve a performance-based payment by the initial reconciliation of the fourth performance period must elect to either enter the two-sided risk track or to discontinue participation in OCM." | 25.07.1 |
|  | 25.07.2 |
|  | 25.07.3 |
| "CMS monitors for quality of care and model integrity through a number of data sources, such as claims data, practice-reported data, and site visits to the practices. Administrative and claims data, as well as information from the data registry, provide information on changes in clinical practice patterns, such as the number of episodes triggered for a specific cancer diagnosis; changes in use, such as shifting of services inside or outside of an episode; and clinical outcome measures, such as hospitalizations and progression-free and overall survival. Practice-reported data show how resources are used to implement the model. Site visits provide an opportunity to verify information and monitor activities that cannot be measured through other data sources, such as accuracy of data submission and implementation of OCM Practice Redesign Activities." | 25.08.1 |
| "This is because, for a variety of public policy reasons, certain health care entities are paid in indirect or supplemental ways. These complex cost accounting systems change the direct relationship between the provision of medical care and payment for that care, complicating the financial alignment of payers and providers necessary for the effective implementation of an EPM. For these reasons, practices that are part of or have a formal or written agreement with one of the excluded entities for the routine provision of outpatient chemotherapy to Medicare beneficiaries with cancer are excluded from the model." | 25.09.2 |
| "CMS has launched a number of alternative payment models since the passage of the Affordable Care Act in 2010. Most of these models use reductions in growth in health care expenditures and performance on quality metrics as bases for performance or shared savings payments. When two models both assume responsibility for a beneficiary for an overlapping period of time, savings or excess spending must be allocated between the participants in the overlapping models. Examples include a beneficiary aligned to an Accountable Care Organization (ACO) who receives cancer care at an OCM practice and an OCM beneficiary, either admitted to a hospital participating in the Bundled Payments for Care Improvement Initiative for a clinical condition that resulted in hospital admission, or receiving lower extremity joint replacement surgery in a hospital participating in the Comprehensive Care for Joint Replacement (CJR) model. In the ACO/OCM example, CMS decided that when a beneficiary is aligned to both an ACO and an OCM practice, the savings (or excess spending) that the OCM practice or ACO generates for that beneficiary would accrue exclusively to the OCM participant and that savings allocated to Medicare for the OCM discount are not counted as savings by an ACO. This is analogous to the arrangement between ACOs and other voluntary CMS bundled payment models, including the Bundled Payments for Care Improvement Initiative. In the event that a participating PGP is also part of an ACO and the ACO achieves shared savings in the ACO reconciliation, CMS would recoup from the OCM participant a portion of the OCM Medicare discount amount that was paid to the ACO as shared savings for beneficiaries who are also aligned to the ACO." | 25.10.1 |

# **References Appendix A**

1. Bushnell BD. Bundled payments in orthopedic surgery. *Orthopedics.* 2015;38(2):128-135.

2. Hussey PS, Ridgely MS, Rosenthal MB. The PROMETHEUS bundled payment experiment: slow start shows problems in implementing new payment models. *Health Aff (Millwood).* 2011;30(11):2116-2124.

3. Miller HD. *Ten Barriers to Healthcare Payment Reform And How to Overcome Them.* Center for Healthcare Quality & Payment Reform;2012.

4. Pham H, Ginsburg P, Lake T, Maxfield M. Episode-Based Payments: Charting a Course for Health Care Payment Reform. Washington, DC: National Institute for Health Care Reform. 2010.

5. Mechanic R. *Medicare’s Bundled Payment Initiatives: Considerations for Providers.* Washington, DC: American Hospital Association;2016.

6. Averill RF, Goldfield NI, Hughes JS, Eisenhandler J, Vertrees JC. Developing a prospective payment system based on episodes of care. *J Ambul Care Manage.* 2009;32(3):241-251.

7. Bailit M, Burns M, Houy M. *Bundled Payments One Year Later: An Update on the Status of Implementations and Operational Findings.* Newtown, CT: HCI3;2013.

8. Chee TT, Ryan AM, Wasfy JH, Borden WB. Current State of Value-Based Purchasing Programs. *Circulation.* 2016;133(22):2197-2205.

9. Conrad DA, Grembowski D, Hernandez SE, Lau B, Marcus-Smith M. Emerging lessons from regional and state innovation in value-based payment reform: balancing collaboration and disruptive innovation. *Milbank Q.* 2014;92(3):568-623.

10. Conrad DA. The Theory of Value-Based Payment Incentives and Their Application to Health Care. *Health services research.* 2015;50.

11. Conrad DA, Vaughn M, Grembowski D, Marcus-Smith M. Implementing Value-Based Payment Reform: A Conceptual Framework and Case Examples. *Med Care Res Rev.* 2016;73(4):437-457.

12. George M, Bencic S, Bleiberg S, Alawa N, Sanghavi D. Case study: Delivery and payment reform in congestive heart failure at two large academic centers. *Healthcare.* 2014;2(2):107-112.

13. HFMA. *Value-Based Payment Readiness.* Louisville, KY: Healthcare Financial Management Association;2015.

14. Hussey PS, Mulcahy AW, Schnyer C, Schneider EC. Closing the quality gap: revisiting the state of the science (vol. 1: bundled payment: effects on health care spending and quality). *Evid Rep Technol Assess (Full Rep).* 2012(208.1):1-155.

15. Matchar DB, Nguyen HV, Tian Y. Bundled Payment and Care of Acute Stroke: What Does it Take to Make it Work? *Stroke.* 2015;46(5):1414-1421.

16. Miller HD. From volume to value: better ways to pay for health care. *Health Aff (Millwood).* 2009;28(5):1418-1428.

17. Porter ME, Kaplan RS. How Should We Pay for Health Care? *Harvard Business School Working Paper.* 2015;15-041.

18. RAND. *Analysis of Bundled Payment.* Santa Monica, CA: RAND Corporation;2009.

19. Ridgely MS, de Vries D, Bozic KJ, Hussey PS. Bundled payment fails to gain a foothold In California: the experience of the IHA bundled payment demonstration. *Health Aff (Millwood).* 2014;33(8):1345-1352.

20. Shih T, Chen LM, Nallamothu BK. Will Bundled Payments Change Health Care? Examining the Evidence Thus Far in Cardiovascular Care. *Circulation.* 2015;131(24):2151-2158.

21. Sutherland JM, Hellsten E, Yu K. Bundles: an opportunity to align incentives for continuing care in Canada? *Health Policy.* 2012;107(2-3):209-217.

22. Berenson RA, Upadhyay DK, Delbanco SF, Murray R. *Payment Methods and Benefit Designs: How They Work and How They Work Together to Improve Health Care.* Urban Institute / Catalyst for Payment Reform;2016.

23. Wojtak A, Purbhoo D. Perspectives on Advancing Bundled Payment in Ontario's Home Care System and Beyond. *Healthc Q.* 2015;18(1):18-25.

24. Joynt Maddox KE, Sen AP, Samson LW, Zuckerman RB, DeLew N, Epstein AM. Elements of Program Design in Medicare's Value-based and Alternative Payment Models: a Narrative Review. *J Gen Intern Med.* 2017;32(11):1249-1254.

25. Kline RM, Muldoon LD, Schumacher HK, et al. Design Challenges of an Episode-Based Payment Model in Oncology: The Centers for Medicare & Medicaid Services Oncology Care Model. *J Oncol Pract.* 2017;13(7):e632-e645.
