# Supplementary material for: Choices and options in the care procurement process of bundled payment contracts: A literature-based overview from a payer’s perspective
Source: PLoS One. 2026 Jun 15;21(6):e0346366. doi: 10.1371/journal.pone.0346366 (PMC13268200; doi:10.1371/journal.pone.0346366)
Supplement: S2 Appendix — (DOCX) [file pone.0346366.s002.docx]

| **Appendix B. Literature-based (potential) impact of design options** | | | |  |
| --- | --- | --- | --- | --- |
|  | | | |  |
| **PRELIMINARY PHASE: FUNDAMENTAL CHOICES** | | | |  |
| **DESIGN CHOICE 1: Determine the transition strategy from FFS to full bundled payment** | | | |  |
| **DESIGN CHOICE 1** | **DESIGN OPTIONS** | **IMPACT +** | **IMPACT -** |  |
| **Determine the transition strategy from FFS to full bundled payment** | **A. Stick with just limited changes in the FFS-based DRG model** | G. While sticking with the FFS model, just expanding the scope of a DRG payment for inpatient care is essentially a more limited version of an episode-based payment, but could become a building block for a full-fledged episode-based payment system (+) (4.04a.1) | A. When FFS remains the dominant payment method then the effects of VBP reforms on outcomes may be smaller (-) (11.06.2)  B. The DRG system has serious flaws because it reimburses specific procedures or inpatient stays, not overall care for patient conditions - and they are often only for hospital payments, with separate payments made to other providers, and non of them contingent on patient outcomes (-) (17.01.1a)  D. If the scope of the payment is limited to just minor changes in the FFS-based DRG model, then they fall far short of value-based bundled payment principles and represent only modest, incremental steps beyond traditional procedure based FFS models (-) (17.02.2)  E. Although new fee schedule codes could be created to reward evaluation and management activities that had never been specifically paid (e.g. complex chronic pain management), this is challenging because the transaction costs of billing and receiving might be more costly than the service itself (-) (22.02.2)  F. FFS leads to over-investment in units that perform generously-reimbursed services, creating excess capacity and more pressure for supply induced demand for such services. And, it leads to under-investment in unreimbursed or poorly-reimbursed services, even those that contribute to better patient outcomes and that avoid much higher costs later in the patient's care cycle (-) (17.01.1b)  H. Bundled payments are a considerable shift from the traditional FFS model and often seen as a middle ground in the spectrum of health care payment models, but, importantly, they are not representative of global payments, or capitation, in which a health care system is paid a lump-sum payment per attributed patient over a distinct time period, regardless of the number of distinct episodes of care (-) (20.05.1)  I. A win-win-win approach - lower spending for payers, better care for patients, and better margins for providers - is only feasible with significant payment reforms, not with minor tweaks to the FFS payment system (+) (3.01a.1) |  |
|  |  |  |  |  |
|  | **B. Stepped-model approach based on included care** | A. In the early stages of a bundled payment program it may be better to first focus on a small number of types of episodes that are relatively straightforward and only expand to target more complex episodes and constellations of providers in later program stages (+) (4.05.2)  B. Limiting the episode scope of services to hospital care and any subsequent hospital readmissions that occur within the episode window is the simplest form of a hospital-based episode and relatively easy to implement (+) (6.18.1)  C. The impact of the contract for providers can be phased in by gradually expanding the services included in the episode service scope and expanding the length of the episode window (+) (6.17.2)  D. Starting with knee and hip replacements is less complex because these are often well-defined, high-volume procedures with opportunity for savings through care redesign (+) (7.11.2)  E. Taking time to get buy-in from most stakeholders, widespread perceptions that FFS payment is not working, protected time and resources for reform, and capacity to evaluate its impacts slowly creates system readiness which increases the likelihood of providers to adopt, implement, and sustain the payment reform (+) (11.01.1)  F. Viewing bundled payments as partway between volume-based and true population-based payment could be helpful because they are more consistent with the service-line strategies hospitals have adopted over the past decade than with population-based payments and therefore the bundled approach could be more readily undertaken without major change in business models and complex organizational cultures (+) (22.04.1)  G. Creating a stepped-model approach based on included care can reduce the threshold for providers to participate in the contract and gradually increase their readiness to accept more risk (+) (5.01.1)  H. Creating virtual teams of providers based on claims analytics to identify a patient's providers retrospectively can improve collaboration among providers and can be a first step towards bundled payments (+) (9.10.1)  I. A small scale pilot program could be used to test a bundled payment system (+) (18.16.1)  J. Current bundled payment efforts are most likely a transitional stage toward independent provider-payer combinations of integrated care delivery and value-based payment, but managing such competition will first demand more transparent public pricing, a common public performance measurement of price and quality, and antitrust vigilance (+) (9.18.1)  K. Compared to immediately supplanting the previous payment system, a lengthy phase-in period may be warranted given providers’ reported difficulty adjusting administrative staffing, finances, and provider relationships to new reimbursement regimes (+) (14.06.1) |  |  |
|  |  |  |  |  |
|  |  |  |  |  |
|  | **C. Stepped-model approach based on the distribution of risk** | B. Suggesting a variety of payment method options with different incentive structures to providers, that may be phased in over time, can be a more acceptable and manageable increase of financial risk for providers (+) (2.04.1)  C. Starting out with an 'upside only' payment strategy or models that cap potential losses at a certain amount can help limit risk as the provider develops its capabilities (+) (13.02.2)  E. A first explorative step towards risk sharing could be the use of 'virtual bundling,' in which the payer would calculate and distribute any gains or losses without actual agreements among providers (+) (18.16.2)  F. Bundled payment cannot be seen as merely a funding or payment approach; it needs to be part of a larger strategy to align care and establish relationships that bridge across different provider organizations (+) (23.01.3)  G. In a first graduate step towards bundled payments, existing data could be leveraged to mimic ‘virtual bundles’ so that bundled costs could then be compared with provincial reference costs, or with the costs of benchmark providers demonstrating high quality and high efficiency (+) (21.03.1)  H. Not requiring to make any repayments to the payer in (for example) the first year of the contract, can help reduce the threshold for providers to participate (+) (5.13.1)  I. Example: In years 2 and 3 of the contract, the payer can calculate the providers’ repayment responsibility based on target prices with a 2 percent discount. Providers can reduce this discount based on their quality score. A majority will likely achieve sufficient quality for a 1 percent discount, and it may drop to 0.5 percent for top performers. Then, the quality-linked-repayment-discount rises to 3 percent in years 4 and 5, etc. (+) (5.13.2)  J. Although not firm, there are some studies that might suggest that larger incentives have a larger effect on performance (+) (24.12.1) | A. If a period of dual payment methods (i.e. traditional and bundled) exists, additional staffing and support may be needed temporarily to handle the double-duty volume (-) (1.04a.2)  D. The gradual phase-in of risk may introduce challenges for evaluators because the more variation between contracts the harder it is to properly monitor them (-) (14.06.3) |  |
|  |  |  |  |  |
|  | **D. Immediate transition from FFS to full bundled payment (no stepped-model)** | A. Compared to a lengthy gradual phase-in period, immediately supplanting the previous payment system introduces less challenges for evaluators because monitoring contracts is more difficult if there is more variation (+) (14.06.2a) | A. In contrast with immediately supplanting the previous payment system, a lengthy phase-in period may be warranted given providers’ reported difficulty adjusting administrative staffing, finances, and provider relationships to new reimbursement regimes (-) (14.06.2b) |  |

| **DESIGN CHOICE 2: Determine the strategy to align the new payment method with other payers** | | | |  |
| --- | --- | --- | --- | --- |
| **DESIGN CHOICE 2** | **DESIGN OPTIONS** | **IMPACT +** | **IMPACT -** |  |
| **Determine the strategy to align the new payment method with other payers** | **A. Establish alignment agreements with other payers** | A. If payers implement common payment reforms, there will be less differences between the contracting terms of payers, which will prevent providers to face significantly higher administrative costs (+) (3.08a.1a)  B. If payers implement common payment reforms, there will be less differences between the contracting terms of payers, which prevents conflicting incentives that could impede improvements in care or deter providers from participating at all (+) (3.08a.1b)  F. Payer cost-sharing could be designed to encourage the selection of higher-value provider organizations (+) (9.19.1)  G. Such agreements are needed to ensure the fair distribution of payments among payers if patients change insurer during the bundled episode of care (+) (18.13.1)  H. Making agreements is important because when two models both assume responsibility for a beneficiary for an overlapping period of time, savings or excess spending must be allocated between the participants in the overlapping models (+) (25.10.1) | C. Concerns about antitrust law violations often make it difficult for payers to discuss or reach agreement on a common approach to payment (-) (3.08a.1c)  D. Many payers pay for patients located in multiple geographic markets, and they find it more efficient to use the same payment system in all of their markets, even if that results in lack of alignment with other payers in any particular market (-) (3.08a.1d)  E. Employers and other purchasers often demand that payers compete for their insurance business, and therefore payers may fear that employers will penalize them for not being “innovative” if they simply use the same payment models as other payers (-) (e) (3.08a.1e) |  |
|  | **B. Do not establish alignment agreements with other payers** | D. Alignment agreements between (non-government) payers is undesired if the belief is that only government or a quasi-governmental public–private partnership (underpinned by organizational public commitments) should monitor and regulate provision of “public goods,” such as common investments in information infrastructure, agreements on quality, outcomes, and patient experience measurement (+) (10.08.1) | A. In markets with multiple payers, there is an incentive for each individual payer to be a “free rider,” i.e., to avoid the costs of implementing payment reforms while retaining all of the savings generated by providers in response to payment reforms implemented by other payers (-) (3.13.1)  B. If each payer designs bundled payments on its own, each payer may choose to include different services in an episode, use different measures of quality, use different systems for risk adjustment, etc., and, at best, these differences will cause providers to face significantly higher administrative costs; at worst, they will create conflicting incentives that could impede improvements in care or deter providers from participating at all (-) (3.07a1)  C. The potential impact of payment reform will be limited if each payer uses a somewhat different approach to identifying the most efficient providers and if they remain unable to pool data with each other (-) (4.06.2)  E. It is difficult for a provider to change the way patient care is provided, particularly when new staff or infrastructure are required, if only some patients are paid for under a new payment system (-) (16.03.1)  F. Without alignment agreements between payers there is the risk that, instead of eliminating inefficiencies, providers will shift costs to payers who are still using fee-for-service systems (-) (16.03.2)  G. If payer refuse to implement new payment systems, or if they do so in an unaligned way, clients, employers and purchasers could switch to payers who are willing to align (-) (3.09a.1) |  |
|  |  |  |  |  |

| **DESIGN CHOICE 3: Determine the payment strategy to incentivize providers** | | | |
| --- | --- | --- | --- |
| **DESIGN CHOICE 3** | **DESIGN OPTIONS** | **IMPACT +** | **IMPACT -** |
| **Determine the payment strategy to incentivize providers** | **A. Use a retrospective payment strategy** | A. Start with a retrospective payment strategy and then a prospective payment system can be phased in over time by gradually expanding the services included in the episode service scope and expanding the length of the episode window (+) (6.17.1) | A. Retrospective determination of cost savings causes a delay that weakens shared savings incentives (-) (10.10.1) |
|  | **B. Use a prospective payment strategy** | A. A prospectively ﬁxed payment can be expected to elicit stronger behavioral response than a retrospective payment based on parameters not speciﬁed in advance (+) (10.04.1) |  |
|  | **C. Offer both retrospective and prospective payment strategies** | A. Developing a process that offers both prospective and retrospective bundled payment methodologies can be critical to the success of the contract implementation (+) (7.14.1) |  |

| **DESIGN CHOICE 4: Determine the strategies to acquire trust, collaboration and commitment among all involved stakeholders** | | | |  |
| --- | --- | --- | --- | --- |
| **DESIGN CHOICE 4** | **DESIGN OPTIONS** | **IMPACT +** | **IMPACT -** |  |
| **Determine the strategies to acquire trust, collaboration and commitment among all involved stakeholders** | **A. Acquire strong commitment and clear support from senior management (on payer and provider side)** | A. Strong organizational commitment, including clear support from senior management, is a prerequisite to successful implementation of the contract (+) (2.07.1)  B. Commitment to the initiative by top leaders has commonly been identified as key to success of the contract (+) (7.04.1a)  C. Keep senior executives involved during the course of the contract (also if they delegate the project to other, lower level, authorities in their organizations) because otherwise they may lose their commitment, especially when challenges arise (+) (9.12.1) |  |  |
|  | **B. Develop a clear communication strategy to stimulate providers to participate in the contract** | A. Explaining the differences between bundling and unpopular previous payment methods to providers, can reduce their perception that bundling is designed to cut costs regardless of the quality of care (+) (2.06.1)  B. Open communication between the payer and provider can create a receptive environment for innovation (+) (9.13.1)  C. A communication strategy to convince and commit providers to the positive potential of the contract can help to remove impediments to bunded payments by providers who express reluctance to adopt provider risk-bearing models due to mixed experiences (with capitation models) in the past (+) (11.04.1)  D. Clear communication can help to deal with uncertainty and fatigue among providers caused by the pace and scope of the reform, fear of change and instances of lack of trust (+) (11.06.1)  E. Clear and consistent communication about movement toward bundled payments is vital because replacing volume-based FFS carries great uncertainty for all stakeholders, particularly for those that would bear most of the financial risk under the new payment model (+) (11.07.1)  F. Clear communication can prevent initially interested providers to drop out a bundled payment pilot because of a perceived lack of need for it in their own institutions and concerns about the time and effort involved (+) (19.01.2) |  |  |
|  |  |  |  |  |
|  | **C. Involve care professionals in the design of the contract** | A. Involving care professionals in the contract design can reduce the difficulties professionals may have to translate retrospective analyses of typical and potentially avoidable costs from program data reports into their everyday clinical experience (+) (2.11a.1a)  B. Involving care professionals in the contract design can help prevent semantic discussions and skepticism of care professionals about the contract (+) (2.11a.1b)  D. Meaningful involvement of clinical leaders in the contract and delivery reforms gives them recognition, which might create a broader interest in clinical redesign and payment reform (+) (12.03.1)  E. Including clinical leaders in the creation of the bundle helps to make sure the contract reflects processes, outcomes, and costs that clinicians can embrace and take accountability for (+) (17.07.1a)  F. Involving care professionals promotes trust and understanding between care professionals and staff, enabling them to work collaboratively on value improvement (+) (17.07.1b)  G. Reducing the complexity of the contract reduces difficulty in explaining the program to medical practices (+) (11.04.1)  H. Including individual physicians in the process is a crucial element because their decisions influence a large proportion of hospital-borne costs (+) (21.02.3)  I. Ensure that the contract does not contain its own lexicon of terms that are not commonly understood by all stakeholders because what may seem like a mere semantic concern can contribute greatly to implementation delays (+) (2.11.1)  J. The relationship between the provider and its affiliated staff or the culture of collaboration within the organization could facilitate the ability to make substantive changes in care patterns (+) (18.08.1) | C. A pilot contract is likely to come under increasing scrutiny as the input of care professionals becomes larger and more varied as more pilots are implemented, which may reduce the speed of implementation (-) (2.11a.1c) |  |
|  |  |  |  |  |
|  | **D. Align gain sharing methods for care professionals in the bundle** | A. Compensating care professionals based on factors such as quality, teamwork, and overall cost-effectiveness, rather than primarily basing compensation on “productivity,” can determine the provider organization’s success under the new payment system (+) (3.01a.1)  B. If care professionals would not only be paid on the basis of compliance with meeting quality targets but also be given a bonus if exceeding ambitious high-quality targets, then that may be conductive to successful implementation of the bundled payment (+) (15.02.3)  C. A gain sharing method can provide common financial incentives - for provider organizations and care professionals - to control the cost of the bundle, because they keep the savings or bear the cost of overruns if costs differ from the fixed payment (+) (22.02.4)  D. If regulations prohibit providers to establish financial incentive programs for care professionals such as gainsharing arrangements, providers cannot effectively manage a bundled payment contract because it's effectiveness will in large part be dependent on the participation and cooperation of care professionals (-) (6.11.1) |  |  |
|  | **E. Define clear goals and a definition of success that is aligned among all stakeholders** | A. As part of the organizational process, a clear and aligned definition for success might reduce the risk of providers electing not to move forward with bundled payments (despite some successful results) (+) (7.03.1)  B. Sharing priorities and aligning contract goals between the payer and providers before the contract is signed can reduce the risk of further diverging priorities during the course of the contract (+) (9.12.2)  C. Always take the existence of multiple and potentially competing interests among the multiple stakeholders into account because they will inevitably shape the definition of objectives, strength of collaboration, and the pace and fidelity of implementation (+) (11.02.1)  E. Setting unified contract goals is important because collaboration among stakeholders becomes more difficult when changing payment levels and methodologies inevitably reduces income and adds financial risk for some parties (+) (9.04.1)  H. Explaining providers that participating in bundled payments can be a strategy to increase market share may give them a more positive view on the contract (+) (2.07.2)  I. Evaluating bundled payment program design options requires an understanding of not only the design elements themselves, but also of program genesis and intent (+) (24.13.1) | D. Not taking differing perspectives of stakeholders into account can be a principal barrier and will cause difficulty in implementing a unified bundled payment (-) (11.05.2)  F. Evaluating the (aligned) definition of success, can be complicated because of the tension between producing timely, practical evidence and conducting rigorous evaluations and, also, because the most rigorous study designs (to determine success) are usually only feasible when an evaluation experiment is planned in the course of implementation (-) (14.07.1)  G. Aligning goals can be a big challenge because of market competition and because stakeholders often have a history of competing rather than collaborating in the marketplace (-) (9.15.1) |  |
|  | **F. Allow external investments from third parties** | A. External government forces can be a key facilitator and create a receptive environment for implementing the contract (+) (9.11.1)  B. External funding from certain (for example) foundations can raise local awareness and credibility of the project (+) (9.11.2)  C. A continued infusion of outside investment - financial and human capital, in particular - can catalyze and reinforce payment innovation (+) (9.16.2)  D. Targeted public and private investment in technologies and systems required to integrate information and achieve interoperability among disparate health information systems is important to enable value-based payment incentives to be translated into action along the continuum of patient care (+) (10.11.1)  E. Further investment in competencies around data use ranked highest (among respondents in this study) when it came to factors that would best enable success under value-based payment (+) (13.02.1)  F. Adequate resources for program design, administration and provider contracting has been identified as key to success of the contract (+) (7.04.1b) |  |  |

| **PHASE 1: SPECIFY THE BUNDLE** | | | |  |
| --- | --- | --- | --- | --- |
| **DESIGN CHOICE 5: Determine the type of care for the bundle** | | | |  |
| **DESIGN CHOICE 5** | **DESIGN OPTIONS** | **IMPACT +** | **IMPACT -** |  |
| **Determine the type of care for the bundle** | **A. Procedure based** | A. A bundle for acute or elective care is easier to implement due to a more clearly defined clinical pathway and fewer providers (+) (2.08.1)  C. A bundle for acute or elective care is often recommended in early stages of an bundled payment program (+) (4.05.1)  D. Often bundled payment programs start with acute or elective care like hip replacements because these are often well-defined, high-volume procedures with opportunity for savings through care redesign (+) (7.11.1)  E. A bundle for acute or elective care may be more feasible because it are often conditions or procedures with clear begin and end dates (+) (18.03.1)  F. A bundle for acute or elective care may be easier to implement because the duration, beginning and end are fairly clear (+) (18.12.1) | B. A bundle for acute or elective care has less potential for improvements in care delivery (-) (2.08.1)  G. Procedure based bundles remain firmly a volume-based payment method in that it rewards providers for initiating more episodes (-) (22.05.1) |  |
|  | **B. Condition based** | A. Condition based bundled episodes could affect much more health care spending and could create much stronger incentives for care coordination across health professionals and providers (+) (22.03.1)  B. Condition based bundled episodes could counter the volume-inducing incentives of procedure-based episodes (+) (22.03.1)  F. Under condition based bundles (compared to population based bundles) accountability for quality and cost could rest with a single organization that provides all the care, with a lead “most responsible provider” organization that either partners with or sub-contracts for any supplementary services that it requires from other organizations or sectors, or with shared accountability across two or more organizations (+) (23.01.1)  G. Condition based bundles have more potential if they are (1) prevalent across the population served and/or expensive to payers, (2) have limited variation in costs across patients with the same characteristics and (3) have evidence-based clinical care guidelines (+) (23.03.2)  H. A bundled payment for chronic medical conditions is viewed as offering stronger potential for improvements in care delivery because potentially avoidable complications were found to be much more common and costly (+) (2.08.2a)  J. Chronic medical conditions are considered to have the potential for the greatest savings (+) (7.10.1) | C. Chronic condition based episodes are associated with several challenges that must be addressed, particularly for patients with multiple chronic conditions (-) (22.03.2)  D. Condition based bundles are often harder to define and therefore offer providers an incentive to “find” conditions in order to receive a prolonged payment for a condition-specific episode (-) (22.10.1)  E. Condition based bundles are harder to standardize and therefore have a higher potential for gaming (-) (22.10.1)  I. Bundled payment is viewed as harder to implement for chronic medical conditions than for procedures because procedures follow a more clearly defined clinical pathway and involve fewer providers (-) (2.08.2b) |  |
|  |  |  |  |  |
|  | **C. Population based** | B. If a population is characterized by multiple comorbid conditions, then a population based bundle can reduce difficulty to accurately attribute the prehospitalization and posthospitalization services to the specific disease that was the reason for hospitalization (as would for example be the case in a condition based bundle) (+) (6.02.1) | A. Compared to a condition based bundle, a population based bundle (e.g. frail elderly) is more challenging because of the need for integrated care pathways that consider multiple providers involved in care combined with the heterogeneity of complex/chronic client populations in the community (-) (23.01.2) |  |

| **DESIGN CHOICE 6: Determine the patient group or population for the bundle** | | | |  |
| --- | --- | --- | --- | --- |
| **DESIGN CHOICE 6** | **DESIGN OPTIONS** | **IMPACT +** | **IMPACT -** |  |
| **Determine the patient group or population for the bundle** | **A. Choose a patient group that is relatively homogeneous, high volume and with low comorbidity rates** | A. An uncomplicated patient group prevents the possibility that providers may refuse to offer a bundled pricing deal to patients with various comorbidities or other factors that place them at high risk of complications (+) (1.02.1)  B. An uncomplicated patient group decreases the risk that providers will not try to include high-risk patients (+) (1.02.1)  C. A high volume patient group can be important because providers may change their care processes only if the contract changes financial incentives for a sufficient percentage of their patients (+) (11.01.1)  D. Applying the bundle to a large volume of homogeneous patients is often referred to as contributing to the positive impact of bundled payments (+) (15.02.1)  E. Restricting initial bundles to large cohorts of patients with similar risk profiles can be a way to deal with insufficient data to fully riskadjust for the significant variations in the outcomes and costs for a condition due to patient risk factors and comorbidities (+) (17.08.1)  F. By defining a small and narrow patient group it can become easier to reach consensus among stakeholders with different interests (+) (19.03.4)  H. A relatively uncomplicated patient group like hip replacements (with low length of stay outliers) is well-suited for a test with a bundling approach (+) (21.01.1) | G. If the patient group becomes too narrow, it can become difficult to capture an adequate number of patients to make the contract viable (-) (19.03.4) |  |
|  |  |  |  |  |
|  | **B. Choose a patient group that is relatively heterogeneous, with large cost variations and in which multiple providers are involved** | A. By choosing a patient group that is broad, costly and involves multiple providers in different settings, bundled payments have the potential to substantially improve care coordination and to generate savings (+) (20.01.1) | B. Choosing a more complex and heterogeneous patient group (e.g. bipolar disorder) can become complicated because the development of bundled payments may not be feasible for all types of patients (-) (21.03.2) |  |

| **DESIGN CHOICE 7: Determine the length of the bundle** | | | |
| --- | --- | --- | --- |
| **DESIGN CHOICE 7** | **DESIGN OPTIONS** | **IMPACT +** | **IMPACT -** |
| **Determine the length of the bundle** | **A. Based on a clinically logic pre- and/or post-episode period** | A. Monitoring post-episode spending (e.g. for 30 days) to identify any systematic increase can ensure that providers do not shift services out of the episode time period to reduce episode spending (+) (5.05.1) | B. If the selection includes clinical events that occur earlier in the treatment process than the index hospitalization, it may introduce greater expenditure variability into the model (-) (25.02.1a)  C. If the selection excludes clinical events that occur earlier in the treatment process than the index hospitalization, it may offer limited opportunities for quality improvement in the process of care, because they often occurred before the involvement of the medical specialist (-) (25.02.1b)  D. Beginning episodes before the initiation of (for example) chemotherapy would have the disadvantage of dividing primary accountability for the patient’s episode between the surgeon and the oncologist (-) (25.02.1c)  E. Long length of wait times can result in difficulties to calculate pre-hospitalization costs because they can not always be clearly delineated from utilization records (-) (21.02.1) |
|  | **B. Based on a fixed time period (e.g. a year)** | B. For a chronic medical condition, the bundle should be time based ($ per month or year), and for primary care bundled payments should also be time based, covering the full set of primary and preventive care services required for defined segments of patients with similar needs (+) (17.03.2) | A. Creating episodic bundles that encompass an extended time period (e.g. 6 months) would essentially be a limited form of capitation with all the associated insurance risks for providers (-) (6.10.1) |

| **DESIGN CHOICE 8: Determine the method to select care and costs for in- or exclusion from the bundle** | | | |
| --- | --- | --- | --- |
| **DESIGN CHOICE 8** | **DESIGN OPTIONS** | **IMPACT +** | **IMPACT -** |
| **Determine the method to select care and costs for in- or exclusion from the bundle** | **A. Based on historic claims** | A. Basing the length of the episode on the average treatment length in historic claims is most likely to capture discrete treatment courses (+) (25.01.1) | B. In a 'total cost of care model' (that is based on historic claims) the cost of the unrelated services (e.g. a car accident) could increase episode expenditures, and, to the extent that these rare high-cost events also randomly occurred in the historical baseline period, they have to be incorporated into the risk-adjusted benchmark episode price methodology (-) (25.03.1) |
|  | **B. Based on best practices and/or care pathways** | B. If care is selected based on sound clinical logic then - with the input of experienced clinicians - payers can more confidently link financial incentives to those episodes with the expectation that providers could improve care for those episodes (+) (4.02.1)  C. If providers are able to recognize when an episode has begun and understand and reasonably predict the full range of services that a patient might need during the course of a typical episode of that type then that transparency and predictability would help ensure that they can respond appropriately to episode-based payment incentives by enhancing care coordination (+) (4.03a.1)  D. If the selection is based on best practice guidelines then that may stimulate the creation of checklists that professionals should use before, during and after care to ensure that all of the best practice guidelines were being followed (+) (18.09.1)  E. If the selection is based on best practice guidelines then that may stimulate all kind of small innovations like daily 15-minute virtual team huddles, a single phone number, and joint assessments by multiple team members that can improve (remote) care for patients and caregivers and support team functioning (+) (23.05.1) | A. If the care pathway or protocol is not standardized and its outcome is not predictable, then pricing becomes impossible (-) (1.03.1) |
|  | **C. Based on evidence-based clinical guidelines** | B. Building up the expected costs of recommended services based on accepted clinical practice guidelines for care of a specific condition (by using "episode-groupers") has the substantial advantage of the clinical face validity that comes from defining “good care” and specifying the corresponding services (+) (4.01a.1) | A. Services that are recommended in evidence-based clinical guidelines are often only a portion of the cost of an episode of care which creates an underestimation of the actual cost of the episode (-) (2.01.2)  C. Building up the expected costs of recommended services based on accepted clinical practice guidelines for care of a specific condition (by using "episode-groupers") has the substantial disadvantage of maintenance over time as changing standards of care could require resource-intensive updates to the calculations (-) (4.01b.1)  D. Building up the expected costs of recommended services based on accepted clinical practice guidelines for care of a specific condition (by using "episode-groupers") has the substantial disadvantage that the groupers’ underlying algorithms are often not well understood by policy makers and providers (-) (4.01c.1) |
|  | **D. Based on the provider type or specialism that is willing to collaborate** | A. Not including the services of a certain provider type or specialism if they are not (willing to be) at the table, improves the process of team building and contracting discussions and creates a more open and flexible approach that is essential to successfully negotiating a bundled payment contract (+) (7.05.1) |  |

| **DESIGN CHOICE 9: Determine insurance risk mitigations for providers in the bundle** | | | |
| --- | --- | --- | --- |
| **DESIGN CHOICE 9** | **DESIGN OPTIONS** | **IMPACT +** | **IMPACT -** |
| **Determine insurance risk mitigations for providers in the bundle** | **A. Exclude rare high-costs, high-risk patients and/or complex complications from the bundle** | A. In cases in which it is clear that certain kinds of costs cannot reasonably be controlled by a provider, simply excluding these costs (or the situations that lead to them) from accountability altogether should be preferred over using risk adjustment formulas or other complex calculations to correct for this (+) (3.06.3)  C. Excluding indirect medical education, disproportionate share and other special payments from the bundle (which will continue to be paid outside of the bundle) makes price setting easier and can make additional risk adjustment redundant (+) (5.02.3) | B. Excluding high-risk patients contributes to lower volumes, while alternative methods to mitigate risk such as casemix adjustment and stop-loss protection could be used to limit risk without decreasing volume (-) (19.05.6)  D. If there is insufficient consensus in the negotiation over potential exclusions, then this may entail gamesmanship on the part of early participants (-) (19.02.2) |
|  | **B. Allow providers to select a portfolio of (higher-volume) bundles** | A. Allowing providers to select a portfolio of different patient groups (with separate bundled payment contracts per group) can increase volume and reduce the risk for providers of substantial variation in the severity of patients (and number of cost outliers) over a given period of time (+) (5.07.2)  B. Reduce the risk for providers of substantial variation in the severity of patients (and number of cost outliers) by introducing strategies like: allowing them to select a portfolio of bundles to increase volume, purchase reinsurance or contract with facilitator-conveners that are willing to share risk (+) (5.07.1)  C. Providers can mitigate risk by limiting the number of bundles they choose to join, to prevent that they may not always have a statistically significant number of patients within each bundle, which can lead to wide variation in patient-severity, and therefore cost, which can lead to significant financial risk for providers year-over-year (+) (7.09.1) |  |
|  | **C. Do not mitigate insurance risk for providers** | C. Separating performance risk from insurance risk can be difficult and concerns have been raised that efforts aimed at (for example) decreasing admissions might, in a bundled payment, create financial incentives that are misaligned with the goals of improving patient care. Namely, a shift in volumes to higher-acuity inpatients might over-emphasize insurance risks compared to prior years (+) (12.01.1b) | A. Transferring both performance risk and insurance risk to providers, causes bankruptcies when providers take on care of many sick patients without any increase in payment (-) (3.03.1)  B. Separating performance risk (things providers can manage) from insurance risks (thing outside their control), and transfer the former to providers and the latter to payers is the economic principle behind a bundle (-) (12.01.1a)  D. If the payment amount is the same regardless of how sick or how well a provider’s patients are, then that gives the provider a strong and undesirable incentive to avoid patients who have multiple or expensive-to-treat conditions, and it puts them at risk of financial difficulty (-) (16.01.2.1)  E. Not giving a provider accountability for only the types of services and costs that he can control or significantly influence, but also for other services and costs in the bundle over which he has little or no influence, increases risks for that provider (-) (3.03.2)  F. Only if the selection of bundled care includes all clinically related costs caused by care production and excludes all costs caused on random events then the selection is fully controllable by the provider and responsive to the provider’s clinical skills and the systems of care he or she uses (-) (2.02.1) |

| **DESIGN CHOICE 10: Determine the method to hold providers accountable for the quality of care in the bundle** | | | |
| --- | --- | --- | --- |
| **DESIGN CHOICE 10** | **DESIGN OPTIONS** | **IMPACT +** | **IMPACT -** |
| **Determine the method to hold providers accountable for the quality of care in the bundle** | **A. Payment is not tied to quality indicators** |  | A. Without financial rewards for quality outcomes, providers can build substantial wealth providing loosely indicated and/or poorly performed procedures (-) (1.06.1) |
|  | **B. Payment is tied to quality indicators (that providers can control)** | A. By connecting quality outcomes to payment, providers are incentivized to provide higher quality at lower cost or suffer financial consequences (+) (1.06.2)  B. Connecting quality standards to payment could minimize the possibility for a reduction in both necessary and unnecessary care (+) (18.02.1)  C. Adding quality measures to the contract will minimize the possibility of providers to cut back on appropriate care as well as on unnecessary care (+) (18.11.1)  E. Only holding providers accountable for aspects of quality they can control will likely prevent resistance of physicians and hospitals to participate in the contract (+) (3.06a.2) | D. Developing appropriate quality measure to connect to payment may be more difficult in some areas of clinical care because they do not have well established national guidelines (-) (18.11.1) |
|  | **C. Payment is tied to quality indicators on a multi-provider level** | A. Measuring quality based on all participating providers can help prevent unreliability of the data due to small numbers of patients involved (+) (3.12.1) |  |
|  | **D. Use a neutral third-party, provider advisory counsil, or community organization to define, collect and analyze quality data** | A. Using a neutral third-party or community organization for quality data will lead to patient-reported information that is objective, reliable, and comparable (+) (3.11.1)  B. A provider advisory council can identify and define quality measures that the payer collects and reports back to the providers and the data collected can function as a "toll gate," meaning that it must be reported before any savings that result from the bundle is distributed to the providers (+) (7.07.1) |  |

| **DESIGN CHOICE 11: Determine the set of quality indicators for the bundle** | | | |  |
| --- | --- | --- | --- | --- |
| **DESIGN CHOICE 11** | **DESIGN OPTIONS** | **IMPACT +** | **IMPACT -** |  |
| **Determine the set of quality indicators for the bundle** | **A. Measure and monitor a broad set of quality indicators** | B. Measuring process quality, health outcomes and transaction price measures has the potential to improve the tradeoff between the higher relative measurement costs of paying on the basis of value and the potential patient health benefits of doing so (+) (10.01.3)  C. If there is insufficient valid information on specific patient outcomes, payers can, at best, rely on more generic, easy to measure outcomes, such as patient mortality, readmission rates and potentially avoidable complications (PACs) - and as outcome measurement and reporting begins to grow rapidly, this constraint will be released (+) (17.06.1)  D. Using a broad set of measures may spur providers to undertake more intensive system-based approaches to overall quality improvement (+) (24.02.1)  E. A broader set of measures can prevent "teaching to the test," meaning that when only a limited number of outcomes are measured, others - which may be equally important to patients and clinicians - are neglected (+) (24.03.1)  F. Phasing a broad set of measures in and out (rather than choosing a small static set) may be a useful strategy (because studies show that performance remains high on phased out measures) (+) (24.03.2)  G. If the bundle is intended to change the way care is delivered across conditions, then a broader set of measures is necessary (+) (24.04.2)  H. The appropriate scope of measures for a given bundle will depend on its goals, therefore one strategy may be to have specific, targeted programs for the highest-priority conditions or issues and broad-based, frequently updated programs to improve care more generally (+) (24.04.3)  I. By using a number of different data sources (such as claims data, provider-reported data, and provider site visits) a broad set of different aspects of the quality of care is measured (+) (25.08.1)  J. Adding patient experience measures into the quality assessment allows both the provider and the payer to monitor how well the patients perceive their care (+) (7.07.2) | A. Requiring providers to measure, report, and improve on a large number of quality measures can be a disincentive to provider participation, particularly if the quality measures demand changes that go far beyond the resources and flexibility provided in the payment system (-) (3.06a.1) |  |
|  |  |  |  |  |
|  | **B. Measure and monitor a narrow set of quality indicators** | B. A narrow, more targeted, set of measures may be less administratively burdensome and could make critical areas for improvement especially salient (+) (24.02.2)  C. If the bundle has a specific thematic focus, then a narrow (targeted) set of measures is most appropriate (+) (24.04.1) | A. Compensating providers on outcomes only is potentially unreliable and unfair due to a time lag between ultimate health outcomes (e.g. percentage of elderly that returns home within 1 year) and multiple influences (e.g., care from different providers, patient response, and comorbidities) beyond provider control (-) (10.01.2) |  |

| **DESIGN CHOICE 12: Determine the type of targets to measure performance of providers in the bundle** | | | |  |
| --- | --- | --- | --- | --- |
| **DESIGN CHOICE 12** | **DESIGN OPTIONS** | **IMPACT +** | **IMPACT -** |  |
| **Determine the type of targets to measure performance of providers in the bundle** | **A. Absolute performance targets** | B. Absolute benchmarks give providers specific targets to meet, which may be more meaningful to clinical leaders and frontline staff and may encourage collaboration across providers (+) (24.05.1) | A. Only measuring absolute performance targets, only rewards providers for achievement (not improvement) (-) (24.01.3)  C. Absolute benchmarking has much less financial certainty for payers because it does not allow them to prospectively assure budget neutrality by ensuring that the number of "winners", or at least their winnings, can balance losses by the "losers" (as is the case with relative benchmarking) (-) (24.06.2)  D. If providers are evaluated only on absolute achievement (and not on relative improvement), then the highest-performing providers at baseline will likely do best (-) (24.07.1) |  |
|  | **B. Relative performance targets** | D. Relative performance assessment allows the payer to prospectively assure budget neutrality by ensuring that the number of "winners", or at least their winnings, can balance losses by the "losers" (+) (24.06.1a)  E. Relative benchmarking may also be more easily implemented because it allows the distribution of observed performance to determine rewards and penalties and does not require a significant duration of pre-data with which to set parameters for expected performance (+) (24.06.1b)  I. Improvement-based comparisons depend much less heavily than achievement-based comparisons on accurate risk adjustment to enable fair comparisons between peers since each hospital or clinician serves as its own comparison group (+) (24.08.1) | A. Since the performance of peers is not controllable, whereas the provider’s own behavior is directly under his or her inﬂuence, relative performance targets cause a lack of control for providers that weakens incentive effects (-) (10.05.1)  B. Only measuring relative performance targets, only rewards providers for improvement (not achievement) (-) (24.01.4)  C. Relative benchmarks may feel more abstract to providers and discourage their collaboration (e.g. because even if all hospitals improve their readmission rates, the majority will still receive penalties) (-) (24.05.2)  F. If providers are evaluated only on relative improvement (and not on absolute achievement), then the lowest-performing providers at baseline will likely do best (-) (24.07.2)  G. Only rewarding relative improvement may mean giving financial rewards to providers who have improved, but are nonetheless delivering suboptimal or even substandard care, or, on the other hand, failing to reward persistently excellent performers whose year-upon-year performance changes little (-) (24.07.3)  H. If providers are only judged on relative improvement, a patient viewing a hospital’s rating might not know whether a good score was based on high absolute performance or on poor performance with high improvement over time (-) (24.09.1) |  |
|  |  |  |  |  |
|  | **C. Both absolute and relative performance targets** | A. Measuring both absolute and relative performance targets, rewards providers for both improvement and achievement (instead of just one of the two) (+) (24.01.5)  B. Using some combination of rewarding achievement and improvement may be optimal in most cases because this offers an incentive to organizations to participate even if initial performance is low, while also recognizing high absolute levels of achievement and acknowledging that continued improvement is relatively more difficult at high levels of performance (+) (24.10.1a)  C. Using some combination of rewarding achievement and improvement would likely help consumers directly compare provider quality, increasing transparency and promoting consumer-driven care (+) (24.10.1b) |  |  |

| **PHASE 2: SELECT PROVIDER(S)** | | | |
| --- | --- | --- | --- |
| **DESIGN CHOICE 13: Determine the mandatory or voluntary nature of participating in the contract** | | | |
| **DESIGN CHOICE 13** | **DESIGN OPTIONS** | **IMPACT +** | **IMPACT -** |
| **Determine the mandatory or voluntary nature of participating in the contract** | **A. Mandatory participation** | A. Mandatory participation would provide payers with more comprehensive data on per-episode costs to support the development of bundled payments for later phases (+) (4.05a.1) | B. Mandatory participation suggests that payers will identify the responsible provider(s) and tell them either prospectively or retrospectively which patients and episodes they are responsible for, since it would be far less feasible to ask all providers to identify their own patients in a broad program (-) (4.05b.1)  C. The main disadvantage of mandatory participation is that it essentially limits payers to applying fee for-service payments, because most providers currently do not work in organizations capable of accepting the financial risk associated with bundled payments. Moreover, the fragmented and competitive nature of current provider markets poses substantial barriers to effective collaboration for many providers (-) (4.05c.1) |
|  | **B. Voluntary participation** | A. Do not mandate providers because many of them will voluntarily accept a payment system that gives them the flexibility to deliver the best care to their patients and rewards them for high-quality care at an affordable cost without putting them at risk for costs they cannot control (+) (3.02.1) | B. In a voluntary program payers could allow the smaller number of participating providers to identify their own patients prospectively, which may increase provider buy-in but also increase the risks of “cream-skimming” and “lemon dropping” if providers try to assume responsibility for less-costly patients and avoid more costly ones (-) (4.05a.2)  C. Under voluntary participation payers could also face some fiscal risk, since providers that volunteer would probably already have lower than average costs per episode than non-volunteers (-) (4.05a.3)  D. If the contract is initially voluntary it is likely that high performing systems will be the first to sign up and they may have less room for improvement (and thus less potential for reducing spending) than systems that do not volunteer (-) (18.05.1) |

| **DESIGN CHOICE 14: Determine the completeness of the selected providers in relation to the full cycle of care/patient journey** | | | |
| --- | --- | --- | --- |
| **DESIGN CHOICE 14** | **DESIGN OPTIONS** | **IMPACT +** | **IMPACT -** |
| **Determine the completeness of the selected providers in relation to the full cycle of care/patient journey** | **A. Bundle services of only one provider** | B. Designing a contract with multiple providers can result in decision processes taking a long time, and then people may tend to lose interest and may start to think it’s never going to happen (+) (19.02.1) | A. Bundling payments applied to just one provider (e.g. hospital-based services only) would result in limited savings opportunities (-) (15.01.1) |
|  | **B. Bundle services of more than one providers (but not the full patient journey)** | B. Especially in the case of a chronic condition, including providers in the post-acute period (e.g. secondary prevention and rehabilitation) to an hospital-based bundle creates real opportunity for improving patient outcomes and care efficiency (+) (15.04.1)  C. Bundling services of more than one provider could potentially improve patient experience through improved care coordination and simplification of billing procedures for patients (+) (18.10.1)  E. A bundle that is quite conservative in terms of services included can reduce the amount of diverging basic interests of providers (+) (19.03.1)  F. A bundle that is quite conservative in terms of services included can stimulate providers to participate in the contract (+) (19.03.1) | A. In the case of a hospital bundle, the hospital will be responsible for the payment of pre-hospitalization services but will not be aware of that responsibility until the patient is actually admitted (-) (6.12.1)  D. When bundling services of more than one providers, patient experience could decline if doctors have less time to spend with patients or if patients have fewer choices of physicians (-) (18.10.1)  G. In episodes with high provider variation and low predictability, it may be difficult to assign clear responsibility for the episode to a small enough number of providers to keep payment approaches simple and transparent (-) (4.03.1) |
|  | **C. Bundle the full cycle of care (full patient journey)** | A. When all relevant providers in the patient journey are bundled, the risk is shifted from the payer to the providers which forces them to become much more aware of ways to decrease costs and improve efficiency of the overall service (+) (1.05.1)  B. When the full care cycle is bundled, a therapist will care about medication prices, and a brace shop owner will pay attention to average length of hospital stay (+) (1.05.1)  C. The identification of pre- and posthospitalization services for a relatively healthy patient can be done with a reasonable degree of accuracy (e.g. a pregnancy episode) (+) (6.01.1) | D. Episodes of care in which pre- and posthospitalization services can be identified accurately, constitute just a small proportion of health care expenditures (-) (6.01.1)  E. Bundling the full cycle of care is complex and can result in several setbacks and significant delay in implementation (e.g. if parties can not agree on care to include in the bundle) (-) (15.01.2) |

| **DESIGN CHOICE 15: Determine the governance model to contract participating providers** | | | |
| --- | --- | --- | --- |
| **DESIGN CHOICE 15** | **DESIGN OPTIONS** | **IMPACT +** | **IMPACT -** |
| **Determine the governance model to contract participating providers** | **A. Contract with one provider who can provide all care included in the bundle (without subcontracting)** | A. One provider who can provide all care included in the bundle could control every aspect and therefore effectively manage costs and maximize profit (+) (1.01.1a)  C. Where providers remain legally independent, sustaining relationships may be difficult for example when a hospital, typically the dominant cost center in a procedure-based bundled episode, may dominate the collaboration and act in its own interests, which may not be congruent with the others (+) (22.07.1) | B. One provider who can provide all care included in the bundle often does not exist (-) (1.01.1b) |
|  | **B. Contract with one provider as main contracting party who subcontracts with other providers** | D. The main provider often knows better than the payer which other providers are a good fit for local context (+) (7.05.2)  E. One provider as main contracting party can reduce administrative and contractual challenges (+) (21.04.1) | A. Negotiations between the main contractor and subcontractors on who does what and who gets paid what can become complicated (-) (1.01.2)  B. Most providers have limited infrastructure for paying for services delivered by subcontracted providers (-) (6.13.1)  C. Most providers have limited experience in negotiating prices for services delivered by subcontractors (-) (6.14.1)  F. The main contractor may dominate the collaboration and act in its own interests, which may not be congruent with the subcontracted providers (-) (22.07.2)  G. Contracting with one provider raises concerns because the main contractor (usually a big provider) may dominate the collaboration (with smaller providers) too much (-) (22.09.1)  H. Holding a particular provider who has accepted payment for a particular condition-based episode (as the main contractor) accountable for total health spending (of all providers included in the bundle), can generate conflicts among different physicians caring for different conditions (-) (22.11.1) |
|  | **C. Contract with multiple providers separately** | A. Contracting with multiple providers separately can be the preferred governance model if local context requires it (+) (7.05.3)  B. By contracting included providers separately they would only need to agree on how to distribute surpluses an deficits while a flow of core funding for each entity would be assured (+) (22.09.3a) | C. Contracting included providers separately may to some extent undermine the goal of the contract (-) (22.09.3b) |
|  | **D. Contract with multiple providers separately using a virtual bundle** | A. Paying a virtual provider team can be a way to improve the quality and efficiency of health care in rural areas (+) (11.05.2)  B. Virtual contracts may be better than waiting until an organizational structure is in place that can accept and distribute a bundled payment (+) (16.02.1)  C. Virtual contracts provide an incentive for providers to control costs of care included in the bundle, but without being fully financially responsible for paying all providers (+) (16.02.3) |  |
|  | **E. Contract with a third party intermediate / convening organization** | A. A third party intermediate organization can provide analytic and risk management services, and contract with payers on behalf of the providers (+) (5.06.1) |  |

| **DESIGN CHOICE 16: Determine the criteria to select eligible providers for the contract** | | | |  |
| --- | --- | --- | --- | --- |
| **DESIGN CHOICE 16** | **DESIGN OPTIONS** | **IMPACT +** | **IMPACT -** |  |
| **Determine the criteria to select eligible providers for the contract** | **A. Select providers based on formal characteristics (e.g. patient volume, interoperability and/or quality profile)** | A. Selecting providers with higher patient volume will substantially mitigate random variation in the severity of patients (and number of cost outliers) over a given period of time for the provider (+) (5.07.3a)  B. Selecting smaller providers can work if they are able to select a portfolio of bundles to increase volume, purchase reinsurance or contract with facilitator-conveners that are willing to share risk (+) (5.07.3b)  C. Selecting providers with well-defined, high-volume procedures with opportunity for savings through care redesign can reduce complexity in the beginning (+) (7.11.3)  D. Selecting providers based on interoperability, BI, care standardization, etc., are key provider capabilities for a successful bundle (+) (13.01.1)  E. Setting a minimum patient volume for providers to participate in the contract prevents them from performing an insufficient number of cases to absorb financial risks like unforeseen high cost outlier cases (+)(17.04.2) |  |  |
|  | **B. Select providers based on informal characteristics (e.g. motivation, commitment and/or experience)** | A. Selecting providers who are looking to the future, understand that they must transform their practices to be successful, have physician champions to lead the change, are willing to think outside the box, have leadership that understands the importance of practice transformation to reducing costs, and are early adopters of innovation can reduce implementation complexity (+) (7.12.1)  B. Selecting providers who have already been engaged with innovations in payment models can help to minimize the associated risk (+) (12.02.1) |  |  |
|  | **C. Select providers based on cost-saving opportunities** | A. While selecting providers with high levels of potentially avoidable costs indicate higher potential for savings, providers with a low level of potentially avoidable costs can be good partners too because they believe there may be cost savings opportunity in the typical portion of the bundles as well(+) (7.12.2) |  |  |
|  | **D. Select providers based on the degree of integration in their (local) system** | A. Selecting providers with a high degree of integration are more likely to have made progress on bundled care for complex patient populations (+) (23.02.1)  B. Selecting providers who are centralized and integrated increases the likelihood of success for bundled payment systems (+) (23.02.2) |  |  |
|  | **E. Select (patients of) providers with retrospective statistical attribution rules** |  | A. Using retrospective statistical attribution rules to assign patients to providers means that neither the provider nor the patient knows they are part of the new payment system until after the care is delivered (-) (3.09.1)  B. If providers and payers only find out retrospectively that they are in a new payment system, it will be difficult for them to work together prospectively to change care and prevent unnecessary costs from occurring (-) (3.09.1)  C. If retrospective attribution of patients is not well-administered then a high percentage of patients will not be attributed to a provider, especially in a multi-payer commercial system where patients switch insurer, which results in major problems in the payment of providers (-) (3.04a.1)  D. A disadvantage of retrospectively identifying patients is that responsibility for improving coordination and efficiency could be diffused among many providers that are not completely aware of who else is providing care to the patient and, thus, would likely result in only modest care delivery improvements (-) (4.03a.6)  E. A main disadvantage of retrospective attribution is that it prevents providers from knowing which patients are attributed to them until after the completion of the episode (-) (25.04.1)  F. Retrospective attribution delays episode-specific feedback to the providers until the care episode is complete (-) (25.04.1) |  |
|  |  |  |  |  |

| **PHASE 3: NEGOTIATE, ADJUST AND SIGN CONTRACT** | | | |
| --- | --- | --- | --- |
| **DESIGN CHOICE 17: Determine the level of flexibility of the contract terms** | | | |
| **DESIGN CHOICE 17** | **DESIGN OPTIONS** | **IMPACT +** | **IMPACT -** |
| **Determine the level of flexibility of the contract terms** | **A. Develop model contracts in which terms can be negotiated postcontractual if unexpected events occur** | A. To deal with misaligned stakeholder interests in postcontratual contract negotiations, the contract could provide for having a neutral arbitrator resolve any disagreements (+) (3.07.3a) | B. In postcontractual contract negotiations, the party which is disadvantaged by the unexpected event will be more interested in making an adjustment than the party which benefits from it (-) (3.07.3b) |
|  | **B. Develop model contracts with (pre-contractual) context-dependent terms based on market conditions, organizational form and/or ownership** | A. Developing context-dependent terms opens the possibility to create a different mix of incentive size and structure for a different market context, which will make it a more optimal payment model (+) (10.02.1)  C. Developing context-dependent terms based on organizational form takes the capacity of providers to accept insurance risk into account and induces them to minimize performance risk (because small independent medical practices are poorly equipped to bear signiﬁcant insurance risk for random variation in health status) (+) (10.07.2)  D. Context-dependent terms based on ownership are useful because in general for-profit providers experience larger declines in utilization under bundled payment than their not-for-profit counterparts (+) (14.01.2) | B. The implication of not basing terms on provider ownership for payers is that not-for-proﬁt providers will have weaker incentives than for proﬁts to hold out for above-competitive prices, which will affect the level of contracted prices (-) (10.07.1)  E. Context-dependent terms based on the providers' organizational form, can prevent academic health centers (who prioritize research and teaching in addition to patient care) to be negatively affected by bundled payment (-) (20.06.1) |
|  | **C. Develop model contracts with (pre-contractual) context-dependent terms based on the providers' patient population** |  | A. Context-dependent terms based on the providers' patient population are useful because a bundled payment can have a different effect on average length of stay in high-SES hospitals compared to low-SES hospitals (-) (14.05.1) |
|  | **D. Develop model contracts in which all terms are (pre-contractual) negotiable except for the bundle definition** | A. Creating a model contract in which the payer and provider(s) in negotiations are free to modify any of the provisions (except for the bundle definitions) is time-consuming, but can results in more valuable provider-customized contracts (+) (19.01.1)  B. By using an open and flexible approach, discussions provide an opportunity for providers to see situations differently, and an opportunity for the payer to see what the providers view as challenges (+) (7.05.4a)  C. An open and flexible approach is believed to be essential to successfully negotiate a bundled payment contract (+) (7.05.4b) |  |

| **DESIGN CHOICE 18: Determine the duration of the contract** | | | |
| --- | --- | --- | --- |
| **DESIGN CHOICE 18** | **DESIGN OPTIONS** | **IMPACT +** | **IMPACT -** |
| **Determine the duration of the contract** | **A. Yearly contract** |  | A. The annual reductions in price or increases in outcome targets will work agains provider acceptance of value-based bundles and lead to resistance in negotiations (-) (17.09.2) |
|  | **B. Multi-year contract** | A. With multi-year contracts value-based provider payment can be based on long term changes in outcome over time (+) (3.05.2)  B. Multi-year contracts provide a better opportunity for providers to make changes in care delivery that take time to implement (+) (3.07.1a)  C. Multi-year contracts provide a better opportunity for providers to to reap returns on investments in preventive care and infrastructure (+) (3.07.1b)  D. Multi-year contracts give payers greater ability to control the trend in health-care costs (+) (3.07.1c)  H. Multi-year contracts create incentives for providers to benefit from innovations that improve outcomes and lower costs (+) (17.09.1)  I. Multi-year contracts provide stakeholders with greater ability to support multi-year transformation efforts (+) (3.16.1) | E. The longer the contract, the greater the potential for unexpected events to occur (-) (3.07.2a)  F. The longer the contract the greater the difficulty of building appropriate protections to deal with unexpected events (-) (3.07.2b)  G. The longer the contract the greater the reluctance providers and payers will have to sign (-) (3.07.2c) |

| **DESIGN CHOICE 19: Determine the method for calculating the price of the contract** | | | |
| --- | --- | --- | --- |
| **DESIGN CHOICE 19** | **DESIGN OPTIONS** | **IMPACT +** | **IMPACT -** |
| **Determine the method for calculating the price of the contract** | **A. Based on historic service use and cost patterns (no benchmark with other providers)** | A. A huge advantage of using historical costs is operational ease and the potential for payers to address a broad range of episode types (+) (4.03a.2) | B. A disadvantage of using historical costs is that these type of relative benchmarks do not reflect the ideal patient care for a given episode and, therefore, lack clinical face validity with providers (-) (4.03a.2.1) |
|  | **B. Based on internal cost benchmarks between providers (i.e. data of one payer)** | A. Payment rates based on internal cost benchmarks have the advantage of reflecting the particular health care needs of the population and local medical standards (+) (4.03a.3) |  |
|  | **C. Based on external cost benchmarks between providers (i.e. data of all payers)** | A. Payment rates based on external cost benchmarks avoids grading providers on the curve by holding them to higher standards (+)  B. Payment rates based on external cost benchmarks (e.g. with an all payer database) provides the same fixed standard rather than comparing providers to one another, thereby minimizing the chances that payments would fall to ever-lower levels over time as the average efficiency performance of participating providers improve (+) (4.03a.4.1) |  |
|  | **D. Based on a blend of the provider's historic spending and regional/national average spending** | A. A payer can calculate target prices as a blend of each provider’s historical spending per episode and the regional average spending in the provider’s census division - and then initially base target prices on two-thirds provider-specific spending and one-third regional spending. Then in year 3 prices could be one-third provider-specific and two-thirds regional and in years 4 and 5 target prices will be set at regional average episode spending levels. This way regional (or national) price differences can be gradually removed (+) (5.02.1) |  |
|  | **E. Based on the care pathway and/or guideline-based standards** | A. Payment rates based on guideline-based standards set a high and clinically valid standard for efficiency performance, which would maximize face validity from providers’ perspectives and reassure patients that efficiency improvements would not jeopardize their receiving appropriate care (+) (4.03a.5)  B. Literature evidence shows that in a situation in which there is an accepted and expected treatment pathway, reimbursement based on expected costs for a defined episode of treatment can work well (especially if there is a simple, clearly defined treatment goal; a single diagnosis; and treatment by a single provider) (+) (23.03.1) |  |
|  | **F. Based on Time driven activity‐based costing (TDABC)** | B. The TDABC calculation provides the accuracy and transparency required to allow providers to be confident about their costs and, consequently, the margins they can earn from a bundled payment contract (+) (17.05.1) | A. To be able to better measure the production costs of different services requires serious investments in cost accounting for providers (-) (10.09.2) |

| **DESIGN CHOICE 20: Determine the price negotiation method** | | | |
| --- | --- | --- | --- |
| **DESIGN CHOICE 20** | **DESIGN OPTIONS** | **IMPACT +** | **IMPACT -** |
| **Determine the price negotiation method** | **A. Negotiate a price based on the individual bundle items** | A. In some cases, the total bundled price may not beat the total prices of the individual items, at least in the early days of bundling (+) (1.07.2) | B. Only negotiating total bundle costs (not the individual items) will decrease the line-item transparency that currently exists for payers under FFS, but this will no longer matter once cost-containment responsibilities are shifted to the provider (-) (1.09.4) |
|  | **B. Only negotiate on the total bundle price** | A. Only negotiating on the price of the total bundle changes the traditional search of payers for the best price for each individual product to a search for the best total price which can create savings from alterations of internal logistics and administrative costs (+) (1.07.1a)  B. Only negotiating on the price of the total bundle can create savings in hidden costs and downstream costs (such as the time and human resource costs as result of multiple vs single negotiation) (+) (1.07.1b)  C. Negotiating total bundle costs (instead of individual items) will likely result in increased cost transparency for the patient (+) (1.09.1a)  D. By negotiating total bundle costs it will be relatively easy for a patient to obtain information about the single bundled price for his or her care, which, especially if linked somehow to the patient’s share of that cost, has the potential to significantly change patient behavior (+) (1.09.1b) |  |

| **DESIGN CHOICE 21: Determine the price level for the bundled payment contract** | | | |
| --- | --- | --- | --- |
| **DESIGN CHOICE 21** | **DESIGN OPTIONS** | **IMPACT +** | **IMPACT -** |
| **Determine the price level for the bundled payment contract** | **A. Set a relatively low price (compared to the sum of historic FFS prices)** | B. Bundled payment contracts can only yield savings for payers if a discounted rate is negotiated at the outset or if payment amounts are adjusted downward to reflect the efficiencies achieved after the contract is in place (+) (18.01.1)  D. By setting a target bundle price that is (e.g. 1 to 2%) below what providers were paid under FFS, a participating provider is incentivized to provide efficient care, reducing the number and cost of services contained in the bundle (+) (20.03.2)  F. A target price is often set just slightly (1 to 2%) below the case rate or below projected spending growth - so that a participating provider is incentivized to provide efficient care, while reducing the number and cost of services contained in the bundle (+) (20.03.1) | A. If the payment amount for the bundle is too low, then providers will be unable to deliver quality care (-) (3.03a.1)  C. Expecting to negotiate a price reduction compared to what providers were paid under FFS, may raise conflict with providers since they are concerned about implementation costs and the increased financial risk they would assume, which may render the negotiations difficult and slow (-) (19.04.1)  E. If the payment is not right then, particularly when facing the costs associated with system redesign, providers might perceive the expected benefits of the new contract as insufficient (-) (9.01.3) |
|  | **B. Set a relatively high price (compared to the sum of historic FFS prices)** | B. If the expected net income under the bundled payment exceeds the income under FFS payment, then that helps to induce (predominantly risk-averse) providers to participate in risk-based contracts because it compensates them for bearing insurance risk (+) (10.04.2) | A. If the payment amount for the bundle is too high, then there will be no savings for purchasers/payers and little incentive for providers to reduce costs (-) (3.03a.2) |
|  | **C. Develop multiple price categories within the same bundle (e.g. based on the severity of the patient's condition)** | A. While creating different prices for the same bundle based on the fact that a providers’ unit (fixed) costs will be higher with lower volumes, total spending can still be reduced, even with higher payments per admission, if admissions are being reduced (+) (3.14.2)  B. Taking the acuity of the patient at the time of the episode trigger into account (e.g. by creating different bundle prices) can result in more accurate prices for dramatically different service utilization within the same intervention (e.g. acute vs elective cardiac bypass)(+) (6.04.1)  C. Taking both the patient’s acuity at the time of the episode trigger and the chronic disease burden of the patient at the beginning of the episode window into account simultaneously will help to understand and predict the expected utilization of services and costs incurred during the episode (+) (6.05.1)  D. If the process of allowing every combination of an episode trigger, episode window, and episode service scope to define a unique type of episode would be automated (by computing the historical average resource use of patients) to establish different episode payment amounts, the diversity will remain manageable (+) (6.06.1)   E. The different payment weights are essentially predictive, risk-adjusted payments expressed in terms of clinically meaningful prospective payment categories (+) (6.07.1) |  |

| **DESIGN CHOICE 22: Determine the method for case mix adjustment** | | | |
| --- | --- | --- | --- |
| **DESIGN CHOICE 22** | **DESIGN OPTIONS** | **IMPACT +** | **IMPACT -** |
| **Determine the method for case mix adjustment** | **A. No risk adjustment** | E. A patient’s baseline risk score tends to increase as soon as they become part of a risk-adjusted payment system (because the provider now has an incentive to do complete coding of diagnoses), and this can cause overall spending to increase rather than decrease (+) (3.04.1) | A. Without case-mix adjustment providers will likely not try to include high-risk patients (i.e. gaming) (-) (1.02.1)  B. In the absence of robust risk adjustment, providers may select low-risk patients and avoid those with higher risks (and costs) (-) (14.02.1)  C. In the absence of robust risk adjustment, providers may game the system by changing coding practices to maximize reimbursement for the bundle (upcoding) or by moving services in time or location to qualify for separate reimbursement (unbundling) (-) (14.03.1)  D. If the amount of the payment is the same regardless of how sick or how well patients are, providers get a strong and undesirable incentive to avoid patients who have multiple or expensive-to-treat conditions, and it puts providers at risk of financial difficulty or bankruptcy if they take on large numbers of such patients (-) (16.01.2)  F. Without risk-adjusting the bundled payment, providers may have concerns about the unprotected financial risk that they would incur if they operated on higher risk patients (-) (19.03.2) |
|  | **B. Use relatively simple risk adjustment systems** | A. Including some adjustment for patient severity reduces the risk of financial losses for the providers when they are taking care of sicker patients (+) (18.04.1)  D. Just adjusting the prices for geographic wage index differences, removing indirect medical education, disproportionate shares and other special payments (which will continue to be paid outside of the bundle), and use no additional risk adjustment, can be a relatively simple way to create standardized payment amounts (+) (5.02.2) | B. In the absence of more adequate case mix adjustment, providers may not want to care for the sickest patients for fear of being financially liable for their inherently more expensive care (-) (18.12.2)  C. If the bundled payment amount is significantly higher for patients who are sicker or more complex (i.e. inaccurate), providers may try to code patients as being sicker (-) (18.12.2) |
|  | **C. Use relatively complex risk adjustment systems** | A. Risk adjustment based on complete clinical data on the patient’s past and current patient health conditions (not just on data recorded to support recent claims for payment to a particular health plan), can decrease the effect of an artificial increase in risk scores (+) (3.04.2)  B. Risk adjustment systems that can capture changes in health and outcome over time and take them in to account can prevent penalizing providers for keeping their patients well (e.g. otherwise if a physician helps a patient lose weight or stop smoking, the patient’s risk score would decrease, and as a result, under a risk-adjusted payment system, the physician would receive a lower payment than if the patient had remained unhealthy) (+) (3.05.1)  C. The use of accurate adjustment systems can ensure that sicker patients can receive more services (+) (16.02.6)  D. More accurate risk adjustment could be used to limit risk without decreasing volume (as excluding higher-risk patients from the bundled payment would) (+) (19.05.5)  E. A broader use of EHR's can enable risk adjustment to be based on complete clinical data on the patient’s past and current patient health conditions, not just on data recorded to support recent claims for payment to a particular payer (+) (3.04.3) |  |

| **DESIGN CHOICE 23: Determine the type of stop-loss provisions** | | | |
| --- | --- | --- | --- |
| **DESIGN CHOICE 23** | **DESIGN OPTIONS** | **IMPACT +** | **IMPACT -** |
| **Determine the type of stop-loss provisions** | **A. Stop-loss provisions at the patient level** | A. By including the rare but real risk for major complications of specific patients into a stop loss provision, the risk of high cost for the provider as a consequence of such complications is reduced (+) (1.04.1) |  |
|  | **B. Stop-loss provisions at the level of a single provider** | A. Sizes and cost-sharing parameters for outlier payments could vary between bundled providers, since larger providers will be better able to manage variation in costs (+) (3.06.3)  B. Because risk adjustment may not always fully account for the significant variability in expenditures, stop-loss provisions can limit that risk by capping the amount that any provider in the bundle will be required to pay back to the payer within a given performance period to (for example) 20% of the sum of the benchmark episode prices for all episodes in the performance period (+) (25.07.3) |  |
|  | **C. Stop-loss provisions at the level of multiple providers** | A. This prevents high risk for providers by requiring payers to provide additional payments to providers when the total cost of treating a group of patients significantly exceeds the agreed-to payment level (+) (3.06.2)  B. To financially protect both the providers and the payer use both stop-loss and stop-gain thresholds, for example by capping provider gains and/or losses at an aggregate level (for all bundles they are involved in) and start low (e.g. 5%) and increase the cap yearly (e.g. up to 20%) (+) (5.03.1) |  |
|  | **D. No stop-loss provisions** |  | A. Not including risk limits (e.g. in the case of outliers) increases the disadvantages of case mix adjustment as a suboptimal and insufficiently accurate solution to predict variations in costs (because of the myriad factors that can affect patient costs and outcomes) (-) (3.06.1)  B. Not creating stop-loss measures may raise concern among providers about the unprotected financial risk that they would incur if they operated on higher risk patients (-) (19.03.3) |

| **DESIGN CHOICE 24: Determine the type of risk sharing** | | | |
| --- | --- | --- | --- |
| **DESIGN CHOICE 24** | **DESIGN OPTIONS** | **IMPACT +** | **IMPACT -** |
| **Determine the type of risk sharing** | **A. Only upside risk** | B. A one-sided (upside) risk arrangement minimizes financial risk for providers because they will not be required to pay for losses (+) (25.07.1b) | A. A one-sided risk arrangement, will usually be subject to a larger discount percentage on the bundle price for providers (compared to two-sided risk arrangements) (-) (25.07.1a)  C. The financial impact of upside risk might be limited if quality targets are linked to an absolute reward instead of a relatively increasing reward (-) (24.01.7) |
|  | **B. Only downside risk** | A. Using a penalty for failing to advance health benefit rather than an equally reward for advancing health benefit will more strongly encourage providers to deliver improved health (+) (10.03.1)  B. Including downside risk may provide a more powerful behavioral incentive than bonuses because prospect theory holds that more value is placed on losses than on equivalent gains (i.e. loss aversion) (+) (24.11.1)  C. Including downside risk may provide a more powerful behavioral incentive than bonuses because in general the willingness to accept is often significantly greater than the willingness to pay, suggesting that people require much more to give something up than they would be willing to pay for it (+) (24.11.2)  D. Penalties may also be more economically efficient than bonuses, since bonus programs require paying additional money to high performers in order to incent change among low performers (+) (24.11.3) | E. The financial impact of downside risk might be limited if quality targets are linked to an absolute penalty instead of a relatively increasing penalty (-) (24.01.6)  F. Having two or more providers participating in a shared savings arrangement creates a version of the prisoner’s dilemma: if provider #1 makes a good faith effort to reduce unnecessary services but provider #2 does not, provider #2 would “win” by maintaining its own fee revenues while also potentially receiving part of the savings generated by provider #1. If provider #2 increases its volume of services, it would receive more revenue and also thwart the opportunity for provider #1 to receive any shared savings to offset the revenue it lost (-) (3.01.1) |
|  | **C. Upside and downside risk** | A. Payment according to value would tend to drive patient health beneﬁt and provider net income in the same direction and make them complementary objectives (instead of substitutes) (+) (10.06.2)  B. A two-sided risk arrangement, will usually be subject to a smaller discount percentage on the bundle price for providers (compared to one-sided risk arrangements) (+) (25.07.2) | C. A two-sided risk arrangement increases financial risk for providers because they will be required to pay for losses if the aggregate actual bundle expenditures exceed the target price (-) (25.07.2)  D. A shared savings construction (in general) might turn out to be more difficult to implement than expected (e.g. because providers nor payers may be eager to set aside funds from which to make the bonus payments) (-) (2.12.1)  E. The financial impact of both upside and downside risk might be limited if quality targets are linked to an absolute penalty or reward instead of a relatively increasing penalty or reward (-) (24.01.8) |

| **PHASE 4: IDENTIFY AND INCLUDE PATIENTS** | | | |  |
| --- | --- | --- | --- | --- |
| **DESIGN CHOICE 25: Determine the incentives for patients to use bundled care** | | | |  |
| **DESIGN CHOICE 25** | **DESIGN OPTIONS** | **IMPACT +** | **IMPACT -** |  |
| **Determine the incentives for patients to use bundled care** | **A. Create benefit design changes for patients who use bundled providers** | A. Offering discounted premiums or lower deductibles for patients who establish medical relationships with participating providers can incentivize them to use bundled care (+) (1.08.1)  B. With incentives for clients to favor more efficient and higher quality providers the potential impact of payment reforms is no longer limited by making them invisible to patients (+) (4.06.1)  C. Constructively engaging clients with benefit design incentives might prevent those clients from actually turning against payment reforms by defending the inefficiency of their favorite providers (+) (4.06.3)  D. Value-based benefit design will encourage clients to demand value by increasing their economic stake in health care decisions (+) (9.21.1)  E. If clients who receive bundled care are paying a fixed proportion of the medical bill (cost-sharing), which in total is less than under FFS payment, then the cost to clients would also be less (+) (18.07.1)  F. Developing benefit design changes for patients who use bundled providers (such as lower out-of-pocket costs) prevents a lack of steering patients to bundled providers which may greatly reduce providers’ enthusiasm to participate (+) (19.05.1)  G. How bundled payments affect the behavior of providers crucially depends on how they interact with benefit design - and innovation in benefit design is needed to increase the gained value per health care dollar (+) (22.01.1)  H. Instead of focusing on the theoretical effects of bundled payments, it is important to also consider interaction with a broad array of benefit designs because they can either encourage or frustrate the opportunities for payment reform to improve value (+) (22.02.1) | I. Client cost sharing might cause regulatory problems that require the payer to negotiate for governmental approval of such a scheme, which may result in a significant time delay and uncertainty in the implementation of the bundled payment contract (-) (19.06.1) |  |
|  |  |  |  |  |
|  | **B. Provide transparency in total bundle costs for patients** | A. Presenting the total bundle costs to clients increases transparency and usability for decision making (+) (1.09.2)  B. Increased transparency of the total cost of care for clients has the potential to significantly change patient behavior (+) (1.09.3)  C. “Last-dollar” cost-sharing with patients for expensive services offered by multiple providers will provide a financial incentive to patients to choose the less expensive providers of a high-cost service, which in turn gives providers the incentive to lower their prices (+) (3.10.1)  D. Making the price for an entire episode of care or for an entire year of care transparent will make price comparisons for consumers much easier (+) (16.02.5)  E. As with the current bundled payments for treatments that patients directly pay for (such as IVF and plastic surgery), clients may also demand a payment approach similar to how they pay for almost all other services they purchase (+) (17.02.1) |  |  |
|  | **C. Engage patients with patient satisfaction information** | A. Patient agreement (through collecting and reporting patient satisfaction information) to use the providers who participate in the bundle can assure more coordinated and efficient care (+) (7.15.1) |  |  |
|  | **D. Designate the bundled providers as centers of excellence** | A. Designating bundled providers as centers of excellence can steer higher patient volumes to these providers which improves their enthusiasm to participate in the contract (+) (19.05.2)  B. High-performing providers would not only get higher payments, but they would get additional patients as well - and loss of patients could be an even more powerful stimulus to low performers than payment penalties (+) (4.06.2) |  |  |
|  | **E. Educate and create awareness by referring providers (e.g. GP's) on the advantages of bundled care for their patients** |  | A. Allowing providers to follow their logical impulse to narrow their referrals to favored care providers might compromise patients’ choice of provider (-) (22.06.1) |  |
|  | **F. Accept that not all patients can be incentivized to receive care from bundled providers** |  | A. Although most high-risk patients want care management (e.g. as part of a chronic bundle), not all patients want to participate. In addition, despite being defined as being high risk by their health plans and medical groups, some patients believe they are healthy (-) (9.14.1) |  |

| **DESIGN CHOICE 26: Determine the criteria for patient inclusion and the process for providers to be able to identify them in the data** | | | |
| --- | --- | --- | --- |
| **DESIGN CHOICE 26** | **DESIGN OPTIONS** | **IMPACT +** | **IMPACT -** |
| **Determine the criteria for patient inclusion and the process for providers to be able to identify them in the data** | **A. Attribute patients prospectively (on the provider side) by using clinical identification criteria** | A. Gathering additional data to attribute patients, such as providers identifying which patients they treat, or patients identifying which provider they perceive as the responsible provider may be particularly appropriate if attribution is done prospectively (+) (4.03a.8) | B. Inconsistencies in provider and payer data can lead to differences of 20 percent or more in the number of patients the provider identifies internally and the number identified in the payer data (-) (5.10.1)  C. Allowing providers to do the attribution (i.e. demonstrating positive test results or providing patients with multiple encounters and documenting diagnoses via claims forms) increases the concern that clinicians will make questionable diagnoses to trigger an episode payment (-) (22.12.1) |
|  | **B. Attribute patients retrospectively (on the payer side) by using historic claims data** | A. Retrospectively attributing patients based on claims data would be easier for payers to implement (+) (4.03a.7a)  B. Retrospectively attributing patients based on claims data lowers the risk that providers would engage in favorable selection of less-costly patients (+) (4.03a.7b) | C. Retrospectively attributing patients based on claims data would offer less flexibility for payers to tailor attribution to specific clinical scenarios (-) (4.03a.7.1a)  D. Retrospectively attributing patients based on claims data would offer less flexibility for payers to pay providers prospectively (-) (4.03a.7.1b)  E. Relying on claims data for attribution may not reflect actual care relationships accurately, which might result in the contract having less face validity with patients and providers (-) (4.03a.7.2) |

| **PHASE 5: DELIVER AND MONITOR SPECIFIED BUNDLE** | | | |
| --- | --- | --- | --- |
| **DESIGN CHOICE 27: Determine the payer claims-processing procedure for care related to the bundle** | | | |
| **DESIGN CHOICE 27** | **DESIGN OPTIONS** | **IMPACT +** | **IMPACT -** |
| **Determine the payer claims-processing procedure for care related to the bundle** | **A. Use a manual claims reconciliation process based on the existing FFS infrastructure** | B. Administering bundles manually can help in the first stages to demonstrate that a bundled payment is a win/win strategy for the payer and the providers before moving to automate the process (+) (7.08.1b) | A. Using a manual reconciliation process is quite time consuming (-) (7.08.1a)  C. Processing claims manually makes it impossible to test automated processes for paying or denying claims that were submitted to the payer (e.g. within a pilot) (-) (19.04.2) |
|  | **B. Use an automatic claims reconciliation process based on the existing FFS infrastructure** | A. A process that is build on the existing ffs claims infrastructure does provide the essential ability to identify the services that are part of the bundle (+) (2.09.1a)  E. An accounting tool that analyzes each claim submitted; determines whether it is part of the bundle; and, if it is, adds the payment amount to a running total budget for the case rate creates the ability to identify services that will be subject to bundled payment rather than fee-for-service payment and to modify payments to providers accordingly (+) (2.03.1) | B. A process that is build on the existing ffs claims infrastructure adds to the complexity of existing payment systems (-) (2.09.1b)  C. To develop decision rules that determine whether specific services are part of a bundle and, if so, whether they constitute typical care or potentially avoidable complications is complex and depends on the quality of the information that providers include on claims, which are not designed with the needs of a bundled payment system in mind (-) (2.09.2)  D. The FFS-based billing and transaction systems that remain if the bundled payment claims process is based on the existing FFS infrastructure are incompatible with newer modes of payment (e.g. reference pricing) and impede the advance of value-based payment (-) (9.17.2) |
|  | **C. Adopt a completely new bundled payments claims-processing procedure** | A. A new claims-processing procedure can deal with the key technical issue of claims that are often modified or rejected during the identification procedure for services that are part of the bundle and then need to be resubmitted (+) (2.10.1) | B. Adopting a new operating platform is a big hurdle because most CFOs of payers will look at the cost of adopting these new platforms as an incremental cost when they should, instead, look at it simply as a cost of doing business in the new world of value based health care (-) (7.01.1) |
|  | **D. Use unique identification codes for patients which moves with them (and links the provided care) across different providers** | A. A unique individual identifier which moves with patients across providers facilitates longitudinal analyses of cross-sectional data sources (+) (21.01.2)  B. Unique identification of patients in the data can reduce the incentive for providers to shift high-cost patients to other providers or to affiliated locations who are not participating in the contact (+) (25.05.1) |  |

| **DESIGN CHOICE 28: Determine the moment of bundle price adjustment** | | | |
| --- | --- | --- | --- |
| **DESIGN CHOICE 28** | **DESIGN OPTIONS** | **IMPACT +** | **IMPACT -** |
| **Determine the moment of bundle price adjustment** | **A. Update bundle prices quarterly** |  | A. By updating bundle prices quarterly neither the payer nor the providers know what the update factors will be until six months after the end of each quarter which can be a source of substantial uncertainty for providers (-) (5.04.1)  B. Updating bundle prices quarterly reduces the provider's confidence that gain or loss calculations are accurate and erodes confidence in the program in general (-) (5.04.1) |
|  | **B. Update bundle prices annually** | A. By updating bundle prices annually providers will know their target prices before the start of each performance period (+) (5.04.2) |  |
|  | **C. No update of bundle prices** |  | A. Without a methodology for adjusting episode prices, over time prices may inaccurately reflect actual expenditures for patient care, and have a negative impact on the incentive for providers to provide high-value care (-) (25.06.1) |

| **DESIGN CHOICE 29: Determine the digital infrastructure to share and analyze data** | | | |
| --- | --- | --- | --- |
| **DESIGN CHOICE 29** | **DESIGN OPTIONS** | **IMPACT +** | **IMPACT -** |
| **Determine the digital infrastructure to share and analyze data** | **A. Create a new central multi-stakeholder database** | A. The best solution for all payers might be to contribute their data to a multi-payer database managed by a multi-stakeholder collaborative that can help providers analyze the data while protecting patient privacy (+) (3.08.1)  B. In order to do timely risk adjustment, providers need to be able to determine the (chronic) illness burden of patients at the beginning of the episode (reported from all bundled provider sites) and providing them access to the complete claims history of payers makes that possible (+) (6.16.1)  C. The existence of a national database and robust capacity for managing, encrypting, and opening access to data are important enablers (+) (9.03.1)  D. Developing a common information system (in a multi-stakeholder committee) may accelerate the development of a community-wide data warehouse and electronic medical record (+) (11.05.1)  E. An extensive patient database allows an accurate assessment of profiling the population to be covered in the bundle and tracking actual costs (+) (15.02.2)  F. The development of an all-payer claims database can enable stakeholders to perform claims-based analyses and track performance metrics across organizations and over time (+) (9.01.1) | G. Even if providers have access to claims data, most would not have the analytic capacity to assemble and analyze large claims databases, particularly if the data come from multiple payers (-) (3.08.3)  H. There could be privacy concerns about giving providers patient-identifiable data about all services from other providers in order to find and combine multiple claims records for their own patients (-) (3.08.2) |
|  | **B. Integrate the existing digital infrastructure of bundled providers** | C. Establishing (real-time) data analytics and information sharing capabilities for providers helps providers to analyze the data quickly, transmit information to their physician and post-acute care partners and track patients across the continuum of care (+) (5.08.1)  D. Transparent data sharing is important to obtain provider buy-in, build trust, and motivate transformation (+) (7.06.1a)  E. Transparent data sharing is essential during the contracting process to demonstrate to the providers the logic and fairness of the bundle definition, the appropriateness of the budget and the opportunities for cost savings (+) (7.06.1b)  F. Making data infrastructure interoperable and adaptable facilitates the advance of value-based payment and real-time data supports the delivery systems’ efforts to manage utilization and costs (+) (9.17.1)  B. Views on interoperability are of particular significance, as that is not only where many organizations currently rank weakest but also where nearly 70 percent of financial executives anticipate their organizations will need to be extremely capable in the near future (+) (13.01.2) | A. As a period of dual payment methods (i.e. traditional and bundled) may exist for several years before bundled payments are fully integrated, additional staffing and support for providers may be needed temporarily to handle the double-duty volume (-) (1.04a.1)  G. Overcoming legal and privacy issues in data sharing is challenging but needed in order to (for example) put together third-party nondisclosure agreements (especially for smaller providers) (+) (9.06.1)  H. Sharing clinical and claims data among employers, payers and providers can raise considerations of privacy, confidentiality, control over the data and antitrust law (-) (9.05.1) |

| **DESIGN CHOICE 30: Determine the type of involvement in the redesign of care delivery** | | | |
| --- | --- | --- | --- |
| **DESIGN CHOICE 30** | **DESIGN OPTIONS** | **IMPACT +** | **IMPACT -** |
| **Determine the type of involvement in the redesign of care delivery** | **A. Support the exchange of patient data between providers to improve care coordination** | A. Properly functioning electronic health records are a crucial component in care redesign strategies because they can enable comprehensive data exchange between outpatient and inpatient settings (+) (2.13.1) |  |
|  | **B. Support providers in developing standardized care protocols and processes** | A. Supporting care professionals to review published guidelines and translate them into verifiable, actionable care processes with clear definitions can become the foundation for care process changes that are practical, measurable, and accountable to specific individuals (+) (15.03.1)  B. Supporting providers to develop standard care protocols and longitudinal care plans, establish new staff roles such as nurse navigators, develop systems where physicians or advance-practice clinicians round regularly in post-acute facilities, and establish processes to ensure smooth transitions between care settings that include “warm-handoffs” between providers (+) (5.09.1) |  |
|  | **C. Support providers by facilitating the involvement of neutral third-party coordinating organizations** | A. Third party coordinating organizations can work closely with the staff of providers and ensure that patients make a smooth transition from the hospital to the home or to an appropriate post-acute provider (+) (5.11.1)  B. Such organizations can facilitate the process of banding non-integrated providers together so they can accept financial risk and improve their collective performance (+) (7.02.1)  C. The presence of a neutral convening organization that functions as an honest broker of collaboration among parties with different interests can be a powerful facilitator of payment innovation and can be fundamental to finding common ground among competitors, managing stakeholders’ expectations, and reconciling competing priorities (+) (9.16.1) |  |
|  | **D. No support for providers to develop integrated care networks** |  | A. Without an integrated network of post-acute care providers or sophisticated coordination among providers hospitals are at substantial risk for the care provided-post discharge and could face losses under the bundled payment's financial arrangements (-) (7.16.1)  B. Supporting providers to make changes in laws and policies (e.g. antitrust laws) can help reduce difficulties for (small) providers to work and contract together without the risk of antitrust violations (-) (3.15.1) |

| **DESIGN CHOICE 31: Determine the type of involvement in the distribution of payment among bundled providers** | | | |
| --- | --- | --- | --- |
| **DESIGN CHOICE 31** | **DESIGN OPTIONS** | **IMPACT +** | **IMPACT -** |
| **Determine the type of involvement in the distribution of payment among bundled providers** | **A. Support providers with the distribution of payment** | A. Support from the payer may be beneficial because methods are needed to identify patients who have begun an episode subject to bundled payment and to “attribute” responsibility for their episode of care to a provider organization (+) (2.05.1)  B. Supporting providers with the significant administrative structural changes and technological capabilities that may have to occur for contractual and collaborative relationships between providers can make a bundled payment approach significantly easier to implement (+) (18.14.1)  C. Payers could help providers with mechanisms to guarantee that physicians are not being double paid (via the bundle and separate billing) for their services and that physicians seeing a patient for a problem unrelated to the bundled diagnosis are still paid appropriately (+) (18.14.2) |  |
|  | **B. Involve a third party claims adjudicator to distribute payment** | A. The third party claims adjudicator is able to re-price claims and assign them to the bundle using definitions set forth by the payer(s). In addition, it can implement bundled payments that are either prospectively paid or retrospectively reconciled (+) (7.08.2)  B. Approaching technology vendors to help providers with their lack in capability to pay participating providers in the bundle can result in the development of a new software program that can pay these providers (+) (19.04.3) | C. Using an intermediary who distributes the payment of the main contracting party to each downstream provider can make it unclear what happens and who is responsible if the intermediary doesn't pay the downstream provider(s) (-) (19.05.3) |
|  | **C. No support: providers make their own arrangements to distribute payment** |  | A. For these type of arrangements significant negotiations between providers must occur, which presents a major opportunity for conflict (and collaboration) (-) (1.01.3)  B. Concerns have been raised about the administrative feasibility of establishing accountability and a mechanism for distributing payment (-) (14.04.1) |

| **DESIGN CHOICE 32: Determine the potential side effects to evaluate the contract** | | | |
| --- | --- | --- | --- |
| **DESIGN CHOICE 32** | **DESIGN OPTIONS** | **IMPACT +** | **IMPACT -** |
| **Determine the potential side effects to evaluate the contract** | **A. Evaluate the impact on provider market conditions** | A. Because larger providers will likely be able to bundle care more effectively, monitoring market shares of providers could help to reduce the risk for monopolies that can harm consumers (+) (1.10.1a)  B. Preventing market shares of certain providers to become too large is important, because it can prevent that smaller entities are deprived of the volume needed to maintain economy of scale and it may deter new competitors from even entering the market in the first place (+) (1.10.1b)  C. Evaluate the increase in bargaining power of bundled providers because it could raise prices higher than they would be if those providers were negotiating separately, without their ongoing joint participation in providing services (+) (22.13.1)  D. Evaluating market conditions is important because a lack of significant competition for providers can discourage them from experimenting with different models of payment innovation (+) (9.02.2)  E. Evaluating market conditions is important because the development of large, vertically linked organizations could increase the participating providers’ market shares and pricing power relative to that of private insurers and self-insured employers (+) (9.20.1) |  |
|  | **B. Evaluate the impact on payer market conditions** | A. Evaluating payer market conditions is important because a lack of significant competition for payers can dampen private insurers’ incentive for payment innovation (+) (9.02.1) |  |
|  | **C. Evaluate undesired "gaming" effects** | A. Monitoring potential undesired effects can prevent gaming; including upcoding, the underuse of effective services within the bundle, avoidance of high-risk patients, and an increase in the number of bundles reimbursed (+) (14.01.1)  B. Referrals between providers should also be evaluated because providers have a logical impulse to narrow their referrals to favored other care providers, which might compromise patients’ choice of provider (+) (22.06.2)  C. Evaluate volumes outside the bundle to prevent that providers (in noncompetitive markets) may increase volumes and prices for other services to make up for reduced revenues on the bundles (+) (22.08.1) |  |
|  | **D. Evaluate potential 'crowd-out' effects of financial incentives** | A. Financial incentives (as extrinsic motivators) tend to "crowd-out" intrinsic motivation and it may reduce providers’ commitment to professional norms and their inherent motivation to act ﬁrst and foremost in patients’ interest (which is a risk that can be reduced by also considering to include nonfinancial incentives (e.g. reputation or brand, intrinsic motivation, and altruism)) (+) (10.06.1) |  |

| **DESIGN CHOICE 33: Determine the effect of the contract on care and costs and how to proceed with the contract** | | | |
| --- | --- | --- | --- |
| **DESIGN CHOICE 33** | **DESIGN OPTIONS** | **IMPACT +** | **IMPACT -** |
| **Determine the effect of the contract on care and costs and how to proceed with the contract** | **A. Do not extend the contract** |  | A. Despite some non-financial successes, if there are no statistically significant financial gains for providers after conclusion of the contract they may elect not to move forward with bundled payments (-) (7.03.2) |
|  | **B. Extend the contract (with adjustments if needed)** | A. The longer the contract the better the opportunity for providers to make changes in care delivery that take time to implement and to reap returns on investments in preventive care and infrastructure (+) (3.07.5)  B. If there are competing interests between parties in the suggested contract adjustments, a neutral arbitrator could resolve any disagreements (+) (3.07.4) |  |
