# Supplementary material for: Choices and options in the care procurement process of bundled payment contracts: A literature-based overview from a payer’s perspective
Source: PLoS One. 2026 Jun 15;21(6):e0346366. doi: 10.1371/journal.pone.0346366 (PMC13268200; doi:10.1371/journal.pone.0346366)
Supplement: S3 Appendix — (DOCX) [file pone.0346366.s003.docx]

# **Appendix C. Setup of the expert panel session**

During the (iterative) process described in the paper, the identified relevant text excerpts from the final study sample were collated into comprehensively defined design choices and options. An important part of this process was the expert panel session held on 16 October 2022. More information about this session is set out below.

**The session**

The expert panel session took place in a digital conference room on Microsoft Teams and lasted two hours. It was not possible for the panel to meet in person due to the national Covid restrictions.

**The participants**

The nineteen health care procurement experts who participated in the panel session were all experienced professionals working at four different payer organizations. Five of the nineteen participants had several years of experience in designing and implementing bundled payment contracts. The other fourteen did not have specific experience with bundled payment contracts but were interested in the concept.

**The goals of the session**

The goals of this two-hour session were to externally assess the recognizability and completeness of:

1. The identified design options and choices (tables 2a-g in the results section), and

2. Their positioning in the framework (table 1 in the results section).

**The procedure**

The experts were asked to prepare for a discussion on the operational choices and options that are relevant in the design and implementation of a bundled payment contract from a payer’s perspective. The first part of the session was an introduction, followed by a presentation on the framework and the design choices and design options identified in the literature. The second part of the session was a group discussion. For each procurement phase in the framework the experts were asked three questions:

1. Based on your experience, are all the design choices and options that we identified in the literature recognizable to you? If not, which choices or options are not recognizable to you (and why)?
2. Based on your experience, do you believe any design choices or options are missing? If so, which choices or options do you miss (and why)?
3. Based on your experience, are the design choices currently positioned in the most appropriate or “best-fitting” procurement phase? If not, which choices or options are not optimally positioned (and why)?

**The result**

In general, the experts found the framework, design choices and options (and their positioning in the framework) recognizable and (almost) complete. One new design choice (DC4) was added to the framework as a cluster of (non-MECE) design options that were initially part of other design choices. The experts advocated that ‘trust, collaboration and commitment’ is such a fundamental aspect of bundled payment contracting that it ‘deserves’ its own design choice. We agreed and, accordingly, rearranged some of the design options from other choices into a new design choice. The expert panel session also resulted in several other minor amendments to a small number of design options (in tables 2a-g) and the repositioning of a small number of design choices to a different procurement phase in the framework (table 1).

**Considerations**

Such expert panel sessions involve an inherent risk of potential bias. For example, experts who are already using bundled payment contracts might be inclined to “over-simplify” the design and implementation process, while experts who are not using bundled payment contracts yet might be more inclined to “over-complicate” the design and implementation process, each in order to justify his/her own policy decision. To limit the risk of potential bias, all participants were explicitly asked during the discussions whether their input was based on actual practical experience or more on their own personal opinion.

Please contact the corresponding author if you wish to receive a list of names of expert panel participants.
