# Supplementary material for: Choices and options in the care procurement process of bundled payment contracts: A literature-based overview from a payer’s perspective
Source: PLoS One. 2026 Jun 15;21(6):e0346366. doi: 10.1371/journal.pone.0346366 (PMC13268200; doi:10.1371/journal.pone.0346366)
Supplement: Attachment — Submitted filename: Response to reviewers - Bundled payment design choices.docx [file pone.0346366.s005.docx]

**POINT-BY-POINT RESPONSE TO THE EDITOR AND REVIEWERS**

Submission: PONE-D-25-40522

Title: Choices and options in the care procurement process of bundled payment contracts: a literature-based overview from a payer’s perspective.

1. **Editor comments**

**1.1 I agree with the reviewers. It´s a great article but requires minor revisions, particularly concerning the abstract, a more complete introduction, and some questions regarding the methodological choices.**

Thank you for your positive assessment of our manuscript and for highlighting the areas that required further clarification. In response to the editor’s comments and the detailed feedback from the reviewers, we have made a number of targeted revisions. Specifically, we (1) rewrote the abstract to more clearly state the study objectives and methodological approach, (2) substantially clarified and expanded the description and justification of our methodological choices, including the selective sampling strategy, the saturation process, and the qualitative content analysis, and (3) revised and expanded the discussion by explicitly reflecting on the limitations of this study. We believe these revisions have improved the clarity, transparency, and robustness of the manuscript.

2. **Comments Reviewer 1**

**2.1 The study adopts a qualitative analysis and describes the methodology clearly.**

We thank the reviewer for this positive assessment of our methodological approach and appreciate the time taken to review our manuscript. The comments provided have helped us further improve the clarity and rigor of the paper.

**2.2 The selection through the use of selective sampling until saturation is justified; however, the explanation for the limit of 24 articles from an initial set of 147 is not clear and has not been empirically justified, which requires more details. The decision-making process that leads to saturation is insufficient and does not describe the processes adopted that led to the cut-off in the identification of new options. The omission leads to uncertainty about the robustness of the study sample (n=24). It is suggested that the saturation process be described and justified, what criteria were adopted, how the sample was validated, and how the coding process was carried out. It is also suggested that a flow diagram be included to increase robustness and transparency.**

We thank the reviewer for this important comment and fully agree that the explanation of how the final study sample of 24 articles was reached required further clarification.

Although the manuscript already described the selective sampling strategy and the qualitative content analysis, what was insufficiently explicit is at which analytical level saturation was reached. Saturation was not reached at the level of included articles or individual text excerpts extracted from those articles (step 1 of the qualitative content analysis). Instead, saturation was reached at step 4 of the analysis, in which merged design options were interpreted and categorized into broader, higher-order design choices. At that stage, newly analyzed articles no longer resulted in additional design choices, nor did they meaningfully alter the categorization of existing ones.

To address this concern, we have revised the Methods section to explicitly explain how saturation was operationalized at the level of higher-order design choices rather than raw excerpts (page 8). We also revised the Results section to explicitly describe that saturation was reached because additional articles did not yield new design choices in step 4 of the qualitative content analysis (page 11).

In addition, as the sample of 24 articles was not a random sample from published articles, we now clarify more explicitly that the analysis started with the articles that contained the highest number of previously identified key elements from the initial scoping review, under the assumption that articles rich in key elements were more likely to yield a higher number of relevant text excerpts on design options (page 7 and 8).

**2.3 The results present 33 design choices and 110 unique options, structured in six acquisition phases. This framework is comprehensive and logically organised. But the interpretation of the results, in some parts of the text, mixes the evidence based on the literature and the authors' interpretive inferences. Although the authors mention when they apply their own reasoning, the line between the evidence from the literature and the authors' interpretations is not sufficiently clear. It is suggested that a clear separation be made between the evidence supported by the literature and personal interpretations.**

We appreciate this careful observation and agree that the distinction between literature-based evidence and authors’ interpretive synthesis should be made more explicit.

The aim of this study was not to perform a purely descriptive or linguistic analysis of contract terms, nor to assess the effectiveness of specific bundled payment designs. Rather, the objective was to identify, structure, and interpret design choices and options described in a fragmented body of literature, and to organize them into a coherent procurement framework from a payer’s perspective. Given the lack of standardized contract templates and the confidentiality of many real-world contracts, interpretive synthesis was unavoidable and integral to achieving this objective.

To address the reviewer’s concern, we have revised the Methods section to more clearly explain that interpretation was applied primarily when merging similar options, positioning design choices within procurement phases and deducing logical implications where impacts were not explicitly stated (page 8 and 9). We have revised the Results section by adding that the positioning of design choices within procurement phases was based on the literature where explicitly described, and on the researchers’ interpretation where the impact on subsequent phases could be logically deduced but was not explicitly stated (page 11). We have revised the Discussion section by adding the explicit limitation of interpretative synthesis as part of the methodology.

We also emphasize that this interpretive approach is consistent with the exploratory and framework-building nature of the study and with established qualitative content analysis methodology. These clarifications help delineate more clearly where findings are directly grounded in the literature and where analytical interpretation was applied.

**2.4 The data availability statement indicates that all relevant data are in the study and supporting materials, available upon request. Despite the situation being in compliance with the journal's policy, the supporting data (409 text excerpts and their impact descriptions) are central to the validation of the study. The omission corresponds to a limitation, which considerably limits the replication of the study. It is suggested that the situation be mentioned in the study's limitations.**

We clarify that Appendices A and B together provide the complete set of extracted excerpts and their associated impact descriptions in a structured and traceable manner. The Excel file that was referred to in the manuscript does not contain additional information than what is presented in Appendices A and B. We believe the information contained in the appendices is sufficient to understand, assess, and replicate the analytical steps taken in this study.

To further clarify this in the manuscript we have removed the sentence about the Excel file and now explicitly explain that Appendix A contains the original text excerpts extracted from the included articles. Each excerpt is assigned a unique reference number. These reference numbers are retained throughout the analysis and are used consistently in Appendix B to indicate the textual basis for each reported impact statement. As such, every impact description in Appendix B can be directly traced back to one or more original excerpts in Appendix A via the corresponding reference number(s), enabling transparency and reproducibility of the coding and synthesis process (page 8).

**2.5 The discussion mentions that design choices are context-dependent, interchangeable, and interrelated. The study would benefit from a deeper reflection on the limitations of qualitative synthesis. For example, the validation by the panel of experts provides an important external confirmation, but the composition of the panel (19 experts from four organisations) may introduce contextual biases that affect the generalisation of the study beyond the Dutch or European context. It is suggested that a limitations section be included in which the following are described: i) the possible contextual biases of the expert panel, ii) the fact that grey literature and relevant details of contracts that have not been published may not be fully captured, and iii) the limits of transferring the findings to other contexts outside of Europe.**

We thank the reviewer for this valuable suggestion and agree that a more explicit reflection on contextual limitations strengthens the manuscript. In response, we have expanded the Limitations section to address: 1. the potential contextual bias introduced by the expert panel, which consisted of professionals from multiple organizations operating within the Dutch multi-payer health system (page 28 and 29), 2. the likelihood that relevant unpublished contractual details are not fully captured in the available literature (page 29), and 3. the limits of transferring the findings to contexts with fundamentally different institutional arrangements, such as single-payer systems or more centrally standardized contracting environments (page 29).

We also clarify that the study intentionally adopts a multi-payer perspective, in which payers typically develop contracts in a bottom-up manner and deliberately differentiate contract elements. This context influenced our focus on variation rather than standardization and should be taken into account when interpreting the framework’s applicability to other health systems. These additions provide a more balanced discussion of the strengths and boundaries of the study’s qualitative synthesis.

3. **Comments Reviewer 2**

**3.1 The paper presents an interesting and relevant topic—the care procurement process of bundled payment contracts: a literature-based overview from a payer’s perspective. However, it suffers from several drawbacks that need to be addressed before it can be considered for acceptance.**

We thank the reviewer for her careful reading of the manuscript and for the constructive feedback provided. We have addressed each of the points raised in detail and believe that the revisions have substantially improved the clarity, methodological transparency, and positioning of the study.

**3.2 The abstract needs to be rewritten, especially by clearly indicating the objectives after the background.**

We agree with this comment and have rewritten the abstract accordingly. The revised abstract now explicitly states the objective of the study immediately following the background, clearly articulating the aim to identify, structure, and interpret design choices and options in the care procurement process of bundled payment contracts from a payer’s perspective. In addition, the Methods section of the abstract has been sharpened in line with the revisions in the main text.

**3.3 I advise the authors to have their manuscript professionally proofread prior to resubmission.**

We appreciate this suggestion. The manuscript has been carefully proofread and revised to improve language, consistency in terminology, and overall readability.

**3.4 The introduction should be revised. I do not see the need to focus so heavily on the effects of bundled payments; instead, it would be more important to emphasize the supporting literature on the topic, previous studies, and other empirical work that can serve as reference points.**

We agree with the reviewer that the introduction should more clearly position the study within the existing literature. Accordingly, we reduced the emphasis on the effects of bundled payments by removing the sentence on the empirical evidence for bundled payment effectiveness, and we now situate the study more explicitly within prior empirical and conceptual work on bundled payment procurement (pages 5–6). The revised introduction now foregrounds the complexity of bundled payment contracts in greater depth, clarifying how the present study builds on and extends this body of work by offering a structured framework from a payer perspective.

**3.5 The method is based on a content analysis, which is appropriate, but it relies on only 24 articles. Although saturation was indeed reached, is the inclusion of only 24 articles fully justified?**

We thank the reviewer for raising this point. As also addressed in response to Reviewer 1 (point 2.2 above), we have substantially clarified the sampling strategy and saturation process in the Methods section.

We now explicitly explain that saturation was reached at the level of higher-order design choices (step 4 of the qualitative content analysis), rather than at the level of individual text excerpts. At that stage, additional articles did not yield new design choices nor materially alter the categorization of existing ones. This analytical saturation justified the final sample size of 24 articles. We believe these clarifications sufficiently address concerns regarding the robustness and justification of the study sample.

**3.6 I suggest adding a table summarizing the characteristics/options identified as well as the characteristics of the participants.**

We thank the reviewer for this helpful suggestion. The characteristics and options identified are already comprehensively summarized in Tables 1 and 2a–g, which together provide a structured overview of all design choices and their associated options across the procurement phases.

Regarding the expert panel, we have added a (minor) clarification in the Methods section describing the composition, background, and role of the panel participants (page 10). Given the exploratory and validation-oriented role of the expert panel, we believe that this level of detail is sufficient and appropriate for the scope of the study.

**3.7 The results are very well presented. More than 30 choices and 110 options are identified, organized around the six phases of the procurement process.**

We thank the reviewer for this positive evaluation of the Results section.

**3.8 The discussion is well developed, with clear identification of the advantages and disadvantages of each option. However, although the authors acknowledge this, there is some overlap among categories.**

We agree with the reviewer that some overlap among design choices and options exists and appreciate the opportunity to clarify this point further.

This overlap is largely inherent to the subject matter and reflects the complexity and interrelated nature of bundled payment contract design. Several design choices intentionally address similar underlying challenges (such as provider risk mitigation) but from different analytical or procedural perspectives within the procurement process. We deliberately chose to preserve this richness and variation rather than enforce artificial separation, as doing so would reduce the framework’s practical relevance for real-world contracting. This rationale is now more clearly articulated in the Results sections (page 13).

**3.9 The conclusion is well structured, but I believe it could place greater emphasis on the study’s limitations and suggestions for future research.**

We thank the reviewer for this suggestion and agree on the importance of clearly reflecting on the study’s limitations and directions for future research. In line with the journal’s structure and common academic practice, we have chosen to address limitations primarily in the Discussion section, where they can be contextualized and discussed in relation to the study’s findings.

Accordingly, we have expanded the Discussion section to more explicitly reflect on the limitations of the qualitative synthesis, the contextual nature of the expert panel, and the transferability of the framework to other health system settings (pages 28 and 29). We believe this placement allows for a more substantive and nuanced discussion of limitations than would be possible in the conclusion.

In response to the reviewer’s comment, we have expanded the Discussion section to more explicitly articulate directions for future research, including empirical application and validation of the framework using contract-level data and exploration of procurement strategies across different health system contexts (page 35). In this way, both the study’s limitations and avenues for future research are addressed in the Discussion section, while the conclusion remains focused on providing a concise and direct answer to the research question and summarizing the study’s main contribution.

4. **Journal Requirements**

**When submitting your revision, we need you to address these additional requirements.**

**4.1 Please ensure that your manuscript meets PLOS ONE's style requirements, including those for file naming. The PLOS ONE style templates can be found at https://journals.plos.org/plosone/s/file?id=wjVg/PLOSOne_formatting_sample_main_body.pdf and https://journals.plos.org/plosone/s/file?id=ba62/PLOSOne_formatting_sample_title_authors_affiliations.pdf**

We have reviewed the manuscript and accompanying files to ensure full compliance with PLOS ONE’s formatting and style requirements, including file naming conventions and table formatting.

**4.2. We note that the grant information you provided in the ‘Funding Information’ and ‘Financial Disclosure’ sections do not match. When you resubmit, please ensure that you provide the correct grant numbers for the awards you received for your study in the ‘Funding Information’ section.**

The Conflict of Interest section on the second page of the manuscript now also describes the association of Mr. van der Hijden with Dutch insurance company Achmea, to ensure consistency with the Funding Information section.

**4.3. Thank you for stating the following financial disclosure: Mr. Steenhuis and mr. van der Hijden report a grant from Zorgverzekeraars Nederland (the umbrella organization of eleven health insurance companies in the Netherlands) during the conduct of the study. Please state what role the funders took in the study. If the funders had no role, please state: "The funders had no role in study design, data collection and analysis, decision to publish, or preparation of the manuscript." If this statement is not correct you must amend it as needed. Please include this amended Role of Funder statement in your cover letter; we will change the online submission form on your behalf.**

We have clarified the role of the funder in the cover letter and in the Conflict of interest section of the manuscript by confirming that the funder had no role in the study design, data collection and analysis, decision to publish, or preparation of the manuscript.

**4.4. Please amend the manuscript submission data (via Edit Submission) to include author Dr. Zilveren Kruis.**

We did not amend the manuscript submission data because Dr. Zilveren Kruis is not an existing author / person. Zilveren Kruis is the name of the insurance company author Dr. van der Hijden is affiliated with.

**4.5. Please include a new copy of Table 1 in your manuscript; the current table is difficult to read. Please follow the link for more information: https://journals.plos.org/plosone/s/tables.**

We have split Table 1 into two tables (Tables 1a and 1b) to fit the page and improve readability.

**4.6. If the reviewer comments include a recommendation to cite specific previously published works, please review and evaluate these publications to determine whether they are relevant and should be cited. There is no requirement to cite these works unless the editor has indicated otherwise.**

There were no reviewer comments that recommended to cite specific previously published works.

**4.7. Please review your reference list to ensure that it is complete and correct. If you have cited papers that have been retracted, please include the rationale for doing so in the manuscript text, or remove these references and replace them with relevant current references. Any changes to the reference list should be mentioned in the rebuttal letter that accompanies your revised manuscript. If you need to cite a retracted article, indicate the article’s retracted status in the References list and also include a citation and full reference for the retraction notice.**

We have reviewed the reference list for completeness and accuracy. All changes to references have been checked and, where applicable, reflected in the revised manuscript and mentioned in this rebuttal.
