# Supplementary material for: Choices and options in the care procurement process of bundled payment contracts: A literature-based overview from a payer’s perspective
Source: PLoS One. 2026 Jun 15;21(6):e0346366. doi: 10.1371/journal.pone.0346366 (PMC13268200; doi:10.1371/journal.pone.0346366)
Supplement: Peer review history

# Peer review: Decision Letter 0

**DOI:** 10.1371/journal.pone.0346366.r001

**Authors:** Alexandre Nunes

16 Dec 2025

PONE-D-25-40522

Choices and options in the care procurement process of bundled payment contracts: a literature-based overview from a payer’s perspective

PLOS One

Dear Dr. Steenhuis,

Thank you for submitting your manuscript to PLOS ONE. After careful consideration, we feel that it has merit but does not fully meet PLOS ONE’s publication criteria as it currently stands. Therefore, we invite you to submit a revised version of the manuscript that addresses the points raised during the review process.

- **I agree with the reviewers. It´s a great article but requires minor revisions, particularly concerning the abstract, a more complete introduction, and some questions regarding the methodological choices.**

-->Please submit your revised manuscript by Jan 30 2026 11:59PM. If you will need more time than this to complete your revisions, please reply to this message or contact the journal office at plosone@plos.org. When you’re ready to submit your revision, log on to https://www.editorialmanager.com/pone/ and select the ‘Submissions Needing Revision’ folder to locate your manuscript file.

Please include the following items when submitting your revised manuscript:

- A rebuttal letter that responds to each point raised by the academic editor and reviewer(s). You should upload this letter as a separate file labeled ‘Response to Reviewers’.
- A marked-up copy of your manuscript that highlights changes made to the original version. You should upload this as a separate file labeled ‘Revised Manuscript with Track Changes’.
- An unmarked version of your revised paper without tracked changes. You should upload this as a separate file labeled ‘Manuscript’.

-->If you would like to make changes to your financial disclosure, please include your updated statement in your cover letter. Guidelines for resubmitting your figure files are available below the reviewer comments at the end of this letter.

If applicable, we recommend that you deposit your laboratory protocols in protocols.io to enhance the reproducibility of your results. Protocols.io assigns your protocol its own identifier (DOI) so that it can be cited independently in the future. For instructions see: https://journals.plos.org/plosone/s/submission-guidelines#loc-laboratory-protocols. Additionally, PLOS ONE offers an option for publishing peer-reviewed Lab Protocol articles, which describe protocols hosted on protocols.io. Read more information on sharing protocols at https://plos.org/protocols?utm_medium=editorial-email&utm_source=authorletters&utm_campaign=protocols.

We look forward to receiving your revised manuscript.

Kind regards,

Alexandre Morais Nunes, Ph.D.

Academic Editor

PLOS One

**Journal Requirements:**

1. When submitting your revision, we need you to address these additional requirements.

Please ensure that your manuscript meets PLOS ONE’s style requirements, including those for file naming. The PLOS ONE style templates can be found at

https://journals.plos.org/plosone/s/file?id=wjVg/PLOSOne_formatting_sample_main_body.pdf and

https://journals.plos.org/plosone/s/file?id=ba62/PLOSOne_formatting_sample_title_authors_affiliations.pdf

2. We note that the grant information you provided in the ‘Funding Information’ and ‘Financial Disclosure’ sections do not match.

When you resubmit, please ensure that you provide the correct grant numbers for the awards you received for your study in the ‘Funding Information’ section.

3. Thank you for stating the following financial disclosure:

Mr. Steenhuis and mr. van der Hijden report a grant from Zorgverzekeraars Nederland (the umbrella organization of eleven health insurance companies in the Netherlands) during the conduct of the study.

Please state what role the funders took in the study.  If the funders had no role, please state: "The funders had no role in study design, data collection and analysis, decision to publish, or preparation of the manuscript."

If this statement is not correct you must amend it as needed.

Please include this amended Role of Funder statement in your cover letter; we will change the online submission form on your behalf.

4. Please amend the manuscript submission data (via Edit Submission) to include author Dr. Zilveren Kruis.

5. Please include a new copy of Table 1 in your manuscript; the current table is difficult to read. Please follow the link for more information: https://journals.plos.org/plosone/s/tables.

6. If the reviewer comments include a recommendation to cite specific previously published works, please review and evaluate these publications to determine whether they are relevant and should be cited. There is no requirement to cite these works unless the editor has indicated otherwise.

7. Please review your reference list to ensure that it is complete and correct. If you have cited papers that have been retracted, please include the rationale for doing so in the manuscript text, or remove these references and replace them with relevant current references. Any changes to the reference list should be mentioned in the rebuttal letter that accompanies your revised manuscript. If you need to cite a retracted article, indicate the article’s retracted status in the References list and also include a citation and full reference for the retraction notice.

[Note: HTML markup is below. Please do not edit.]

Reviewers’ comments:

Reviewer’s Responses to Questions

**Comments to the Author**

1. Is the manuscript technically sound, and do the data support the conclusions?

The manuscript must describe a technically sound piece of scientific research with data that supports the conclusions. Experiments must have been conducted rigorously, with appropriate controls, replication, and sample sizes. The conclusions must be drawn appropriately based on the data presented.

Reviewer #1: Yes

Reviewer #2: Yes

**********

2. Has the statistical analysis been performed appropriately and rigorously?

Reviewer #1: Yes

Reviewer #2: N/A

**********

3. Have the authors made all data underlying the findings in their manuscript fully available?

The PLOS Data policy requires authors to make all data underlying the findings described in their manuscript fully available without restriction, with rare exception (please refer to the Data Availability Statement in the manuscript PDF file). The data should be provided as part of the manuscript or its supporting information, or deposited to a public repository. For example, in addition to summary statistics, the data points behind means, medians and variance measures should be available. If there are restrictions on publicly sharing data—e.g. participant privacy or use of data from a third party—those must be specified.

Reviewer #1: Yes

Reviewer #2: Yes

**********

4. Is the manuscript presented in an intelligible fashion and written in standard English?

PLOS ONE does not copyedit accepted manuscripts, so the language in submitted articles must be clear, correct, and unambiguous. Any typographical or grammatical errors should be corrected at revision, so please note any specific errors here.

Reviewer #1: Yes

Reviewer #2: Yes

**********

5. Review Comments to the Author

Please use the space provided to explain your answers to the questions above. You may also include additional comments for the author, including concerns about dual publication, research ethics, or publication ethics. (Please upload your review as an attachment if it exceeds 20,000 characters)

**Reviewer #1:**  The study adopts a qualitative analysis and describes the methodology clearly. The selection through the use of selective sampling until saturation is justified; however, the explanation for the limit of 24 articles from an initial set of 147 is not clear and has not been empirically justified, which requires more details. The decision-making process that leads to saturation is insufficient and does not describe the processes adopted that led to the cut-off in the identification of new options. The omission leads to uncertainty about the robustness of the study sample (n=24). It is suggested that the saturation process be described and justified, what criteria were adopted, how the sample was validated, and how the coding process was carried out. It is also suggested that a flow diagram be included to increase robustness and transparency.

The results present 33 design choices and 110 unique options, structured in six acquisition phases. This framework is comprehensive and logically organised. But the interpretation of the results, in some parts of the text, mixes the evidence based on the literature and the authors’ interpretive inferences. Although the authors mention when they apply their own reasoning, the line between the evidence from the literature and the authors’ interpretations is not sufficiently clear. It is suggested that a clear separation be made between the evidence supported by the literature and personal interpretations.

The data availability statement indicates that all relevant data are in the study and supporting materials, available upon request. Despite the situation being in compliance with the journal’s policy, the supporting data (409 text excerpts and their impact descriptions) are central to the validation of the study. The omission corresponds to a limitation, which considerably limits the replication of the study. It is suggested that the situation be mentioned in the study’s limitations.

The discussion mentions that design choices are context-dependent, interchangeable, and interrelated. The study would benefit from a deeper reflection on the limitations of qualitative synthesis. For example, the validation by the panel of experts provides an important external confirmation, but the composition of the panel (19 experts from four organisations) may introduce contextual biases that affect the generalisation of the study beyond the Dutch or European context. It is suggested that a limitations section be included in which the following are described: i) the possible contextual biases of the expert panel, ii) the fact that grey literature and relevant details of contracts that have not been published may not be fully captured, and iii) the limits of transferring the findings to other contexts outside of Europe.

**Reviewer #2:**  The paper presents an interesting and relevant topic—the care procurement process of bundled payment contracts: a literature-based overview from a payer’s perspective. However, it suffers from several drawbacks that need to be addressed before it can be considered for acceptance.

The abstract needs to be rewritten, especially by clearly indicating the objectives after the background.

I advise the authors to have their manuscript professionally proofread prior to resubmission.

The introduction should be revised. I do not see the need to focus so heavily on the effects of bundled payments; instead, it would be more important to emphasize the supporting literature on the topic, previous studies, and other empirical work that can serve as reference points.

The method is based on a content analysis, which is appropriate, but it relies on only 24 articles. Although saturation was indeed reached, is the inclusion of only 24 articles fully justified?

I suggest adding a table summarizing the characteristics/options identified as well as the characteristics of the participants.

The results are very well presented. More than 30 choices and 110 options are identified, organized around the six phases of the procurement process.

The discussion is well developed, with clear identification of the advantages and disadvantages of each option. However, although the authors acknowledge this, there is some overlap among categories.

The conclusion is well structured, but I believe it could place greater emphasis on the study’s limitations and suggestions for future research.

**********

6. PLOS authors have the option to publish the peer review history of their article (what does this mean?). If published, this will include your full peer review and any attached files.

If you choose “no”, your identity will remain anonymous but your review may still be made public.

**Do you want your identity to be public for this peer review?**  For information about this choice, including consent withdrawal, please see our Privacy Policy.

Reviewer #1: **Yes:**  Ricardo de Moraes e Soares

Reviewer #2: **Yes:**  Andreia Matos

**********

[NOTE: If reviewer comments were submitted as an attachment file, they will be attached to this email and accessible via the submission site. Please log into your account, locate the manuscript record, and check for the action link "View Attachments". If this link does not appear, there are no attachment files.]

To ensure your figures meet our technical requirements, please review our figure guidelines: https://journals.plos.org/plosone/s/figures

You may also use PLOS’s free figure tool, NAAS, to help you prepare publication quality figures: https://journals.plos.org/plosone/s/figures#loc-tools-for-figure-preparation.

NAAS will assess whether your figures meet our technical requirements by comparing each figure against our figure specifications.

# Author comment: Author response to Decision Letter 1

**DOI:** 10.1371/journal.pone.0346366.r002

14 Mar 2026

See ‘Response to reviewers’ document.

Attachment Submitted filename: Response to reviewers - Bundled payment design choices.docx

# Editor report: Decision Letter 1

**DOI:** 10.1371/journal.pone.0346366.r003

**Authors:** Alexandre Nunes

18 Mar 2026

Choices and options in the care procurement process of bundled payment contracts: a literature-based overview from a payer’s perspective

PONE-D-25-40522R1

Dear Dr. Steenhuis

We’re pleased to inform you that your manuscript has been judged scientifically suitable for publication and will be formally accepted for publication once it meets all outstanding technical requirements.

Within one week, you’ll receive an e-mail detailing the required amendments. When these have been addressed, you’ll receive a formal acceptance letter and your manuscript will be scheduled for publication.

An invoice will be generated when your article is formally accepted. Please note, if your institution has a publishing partnership with PLOS and your article meets the relevant criteria, all or part of your publication costs will be covered. Please make sure your user information is up-to-date by logging into Editorial Manager at Editorial Manager® and clicking the ‘Update My Information’ link at the top of the page. For questions related to billing, please contact billing support.

If your institution or institutions have a press office, please notify them about your upcoming paper to help maximize its impact. If they’ll be preparing press materials, please inform our press team as soon as possible -- no later than 48 hours after receiving the formal acceptance. Your manuscript will remain under strict press embargo until 2 pm Eastern Time on the date of publication. For more information, please contact onepress@plos.org.

Kind regards,

Alexandre Morais Nunes, Ph.D.

Academic Editor

PLOS One

Additional Editor Comments (optional):

The authors have made all the revisions required by the reviewers, so the article has the necessary quality to be published.

Reviewers’ comments:

The authors have made all the revisions required by the reviewers, so the article has the necessary quality to be published.

# Editor report: Acceptance letter

**DOI:** 10.1371/journal.pone.0346366.r004

**Authors:** Alexandre Nunes

PONE-D-25-40522R1

PLOS One

Dear Dr. Steenhuis,

I’m pleased to inform you that your manuscript has been deemed suitable for publication in PLOS One. Congratulations! Your manuscript is now being handed over to our production team.

At this stage, our production department will prepare your paper for publication. This includes ensuring the following:

* All references, tables, and figures are properly cited

* All relevant supporting information is included in the manuscript submission,

* There are no issues that prevent the paper from being properly typeset

You will receive further instructions from the production team, including instructions on how to review your proof when it is ready. Please keep in mind that we are working through a large volume of accepted articles, so please give us a few days to review your paper and let you know the next and final steps.

Lastly, if your institution or institutions have a press office, please let them know about your upcoming paper now to help maximize its impact. If they’ll be preparing press materials, please inform our press team within the next 48 hours. Your manuscript will remain under strict press embargo until 2 pm Eastern Time on the date of publication. For more information, please contact onepress@plos.org.

You will receive an invoice from PLOS for your publication fee after your manuscript has reached the completed accept phase. If you receive an email requesting payment before acceptance or for any other service, this may be a phishing scheme. Learn how to identify phishing emails and protect your accounts at https://explore.plos.org/phishing.

If we can help with anything else, please email us at customercare@plos.org.

Thank you for submitting your work to PLOS ONE and supporting open access.

Kind regards,

PLOS ONE Editorial Office Staff

on behalf of

Professor Alexandre Morais Nunes

Academic Editor

PLOS One
